# SORLA up-regulation suppresses pathological effects in aged tauopathy mouse brain

**DOI:** 10.1126/sciadv.aed6825

**Published:** 2026-07-17

**Authors:** Huijie Huang, Christina Huan Shi, Wenqi Yang, Juan C. Piña-Crespo, Jay Bhatnagar, Julian Curatolo, Rabi Murad, Palak Shah, Alex Campos, Alexandra Houser, Rebecca A. Porritt, Giau Van Vo, Qiang Xiao, Tongmei Zhang, Shengjie Feng, Kevin Y. Yip, Timothy Y. Huang

**Affiliations:** ^1^ Center for Neurologic Diseases, Sanford Burnham Prebys Medical Discovery Institute, La Jolla, CA 92037, USA.; ^2^ Center for Data Sciences, Sanford Burnham Prebys Medical Discovery Institute, La Jolla, CA 92037, USA.; ^3^ Bioinformatics Shared Resource, NCI-Designated Cancer Center, Sanford Burnham Prebys Medical Discovery Institute, La Jolla, CA 92037, USA.; ^4^ Proteomics Core Facility, Sanford Burnham Prebys Medical Discovery Institute, La Jolla, CA 92037, USA.; ^5^ NCI-designated Cancer Center, Sanford Burnham Prebys Medical Discovery Institute, La Jolla, CA 92037, USA.; ^6^ Department of Chemistry, The Scripps Research Institute, La Jolla, CA 92037, USA.; ^7^ The Skaggs Institute for Chemical Biology, The Scripps Research Institute, La Jolla, CA 92037, USA.; ^8^ Cancer Genome and Epigenetics Program, NCI-Designated Cancer Center, Sanford Burnham Prebys Medical Discovery Institute, La Jolla, CA 92037, USA.

## Abstract

A role for the trafficking receptor SORLA (Sortilin-related receptor containing LDLR class A repeats) in reducing Aβ levels has been well established; however, relatively little is known with respect to whether and how SORLA can potentially affect tau pathology in vivo. Here, we show that transgenic SORLA up-regulation (SORLA TG) can attenuate pathological effects in aged PS19 (P301S tau) mouse brain, including tau phosphorylation and seeding, ventricle dilation, synapse loss, long-term potentiation (LTP) impairment, and glial hyperactivation. Proteomic analysis indicates attenuation of PS19 profiles in PS19/SORLA TG hippocampus, including pathological changes in synapse-related proteins and key drivers of synaptic dysfunction such as ApoE and C1q. Single-nucleus RNA sequencing analysis reveals suppression of PS19 signatures with SORLA up-regulation and identifies a previously unrecognized involvement of Sema4D-PlexinB1/B2 signaling in glial pathology. In contrast, SORLA deletion exacerbates tau seeding and aggregation, glial hyperactivation, and PlxnB1/B2 induction in PS19 hippocampus. These results indicate that SORLA confers neuroprotection against tau toxicity in the PS19 mouse brain.

## Teaser

The AD risk gene *SORL1* suppresses degeneration and key transcriptomic changes in a tauopathy mouse model.

## INTRODUCTION

Alzheimer’s disease (AD) is an age-related dementia disorder affecting the elderly. In AD, amyloid-β (Aβ) plaques and neurofibrillary tangles (NFTs) comprising hyperphosphorylated tau accumulate in brain. While most AD cases occur sporadically, a minority of cases (<5%) (*1*) represent an inherited familial form of AD with early-onset symptoms in people under 65 years old. Individuals carrying genetic variants of the amyloid precursor protein (APP), as well as genes encoding APP processing machinery within the γ-secretase complex (PS1 and PS2), manifest early-onset AD at near-complete levels of penetrance (*2*). Whether and how other causal genetic factors can influence sporadic AD remains less clear.

Recent genome-wide association studies have linked multiple gene variants with altered AD risk, including the class I membrane receptor endosomal trafficking factor, SORLA, or “Sortilin-related receptor containing LDLR class A repeats” (encoded by the *SORL1* gene, also known as LR11) (*3*, *4*). SORLA is a type I transmembrane trafficking component comprising N-terminal VPS10, EGF-like/YWTD, LDLR, and FN3 repeats within the ectodomain, as well as a short cytoplasmic tail (*5*). *SORL1* variants were initially identified in association with late-onset AD, and siRNA-mediated SORLA down-regulation enhanced amyloidogenic APP trafficking and processing (*6*). Subsequent studies have further demonstrated the association of *SORL1* coding variants with late-onset AD (*7*–*10*). SORLA variants have also been identified in inherited late-onset AD (*11*), and familial SORLA variants have also been linked to early-onset AD in European populations (*12*–*14*). Large-scale exome-sequencing studies implicate a significant correlation between loss-of-function SORLA variants and AD onset (*15*), and growing evidence suggests that some SORLA variants such as the dimerization-deficient Y1816C (*16*) or R953C (*17*) may be linked to dominant causality in AD (*18*).

SORLA is a component of the retromer endosomal trafficking complex (*19*–*21*). Expression of retromer components have been shown to be down-regulated in AD (*21*–*24*) and reduced SORLA expression was also linked to AD (*25*). Deletion of murine SORLA (*Sorl1*) showed enhanced Aβ accumulation in cortex (*26*). Moreover, transgenic expression of human SORLA reduces Aβ accumulation and pathology in APP/PS1 AD mice (*27*). Neuroprotective effects of SORLA on Aβ accumulation and generation are well documented; SORLA can reduce Aβ levels through various mechanisms, including trafficking of APP from the endosome to the Golgi (*19*) or cell surface (*20*), thus attenuating amyloidogenic APP cleavage by BACE1 at late endosomes. In addition, SORLA reduces APP and BACE1 (*28*) interactions while directly binding and facilitating Aβ internalization and lysosomal degradation (*27*, *29*). SORLA haploinsufficiency in minipigs results in elevated Aβ and tau CSF levels with little change in brain Aβ/tau pathology (*30*) and SORLA deletion increases phosphorylated tau levels in iPSC-derived neurons (*31*). This suggests that in addition to trafficking and internalizing Aβ (*27*, *29*), SORLA may also potentially affect tau accumulation.

SORLA is expressed in both neurons and glia in mouse and human brain (*32*, *33*). To explore the specific role of SORLA in tau pathology and its concurrent effects on various brain cell types in vivo, we crossed a mouse line overexpressing human SORLA from the murine *Rosa26* locus (*27*) with the PS19 tauopathy mouse line expressing the P301S tau variant linked to FTDP-17 (*34*). Using complementary approaches, we show that SORLA overexpression attenuates ventricular enlargement, tau phosphorylation and seeding, synaptic loss, impaired synaptic plasticity, and glial hyperactivation in the PS19 mouse brains. These findings reveal a protective role for SORLA in multiple aspects of tauopathy pathogenesis and highlight its potential as a therapeutic target.

## RESULTS

### SORLA up-regulation attenuates pathological features in aged PS19 hippocampus

**Fig. 1.**
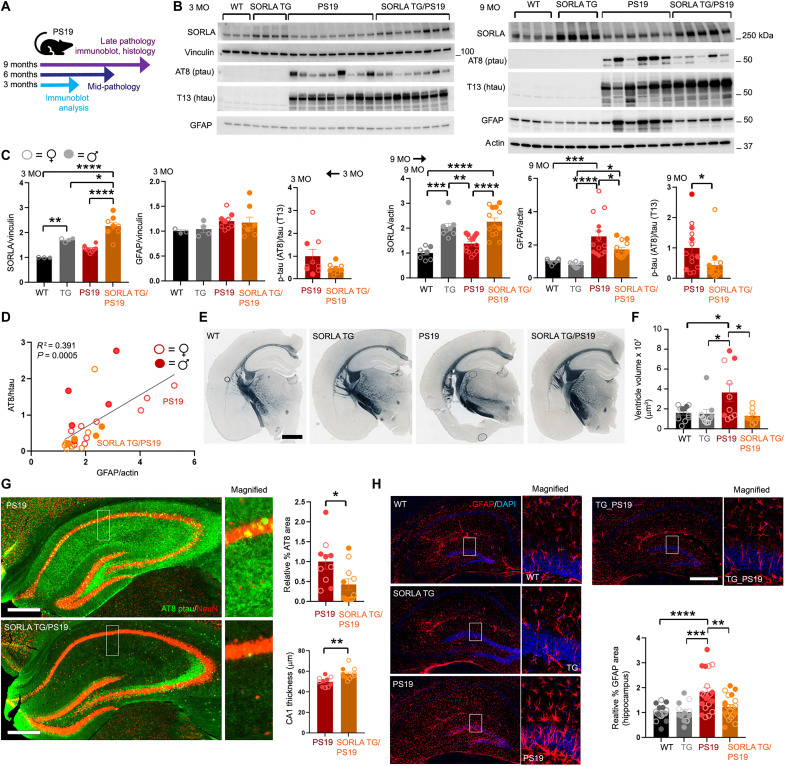
SORLA up-regulation attenuates pathological changes in PS19 hippocampus. (**A**) Experimental workflow and pathological stages in PS19 mouse brain. (**B** and **C**) Representative immunoblots of hippocampal RIPA-SDS lysates from 3-month-old (MO) and 9 MO wild type (WT), SORLA TG (TG), PS19, and SORLA TG/PS19 animals for SORLA, vinculin/actin, pS202/T205 tau (AT8), total human tau (T13), or GFAP. Relative band intensity values were normalized to WT (SORLA, GFAP) or PS19 (AT8/T13 tau) animals (WT or PS19 levels were set to 1.0). Individual plots represent individual animals. (**D**) Correlation between AT8 ptau and GFAP levels in 9 MO hippocampus from relative band intensity values quantified in (C). (**E**) Representative images of Sudan black-stained coronal sections from 9 MO animals; scale bar, 1 mm. (**F**) Ventricle volume was quantified from matched coronal sections in 30-μm slices and is shown for individual animals. (**G**) Representative images from histological hippocampal sections from 9 MO animals stained for AT8 ptau (green) and NeuN (red); scale bar, 0.4 mm. Adjacent graphs show quantification of AT8 ptau area and the CA1 NeuN+ granule cell layer thickness from stained sections. (**H**) Representative images from 9 MO hippocampal sections stained for GFAP (red) or nuclei (DAPI, blue); scale bar, 500 μm. Adjacent graph shows quantification of relative percentage GFAP staining area. Note: In addition to animals included in (E) and (F), GFAP staining was performed on additional animals in (H), and sections that were damaged or did not match coronal regions were excluded from ventricle and hippocampal volume analysis in (F). All graphs represent mean ± SE. Statistical analysis was determined by two-way ANOVA with Tukey’s multiple comparisons in (C), (F), and (H). Unpaired Student’s *t* test was used in (C) and (G); simple linear regression was used in (D), *P < 0.05, **P < 0.01, ***P < 0.001, ****P < 0.0001. Empty and filled plots represent female and male animals as indicated.

The PS19 (P301S tau) mouse, a widely used model of tau pathology, manifests pathological features by 8 to 9 months of age (Fig. 1A) (*34*). We crossed PS19 animals with a mouse line expressing human SORLA under the regulation of a CAG promoter at the *Rosa26* locus (SORLA TG or TG) (*27*). Comparing AT8-reactive (pS202/T205) ptau levels in soluble hippocampal lysates from 3- and 9-month-old (MO) animals, we observed variability in AT8 levels in PS19 hippocampus (Fig. 1, B and C, and fig. S1A). Similar variations have been observed in other studies characterizing PS19 (*35*, *36*) or PS19 mice combined with human APOE4 knock-in (KI) alleles (*37*–*40*). We observed low AT8 ptau levels in 3 MO PS19 hippocampus and SORLA TG/PS19 animals (Fig. 1, B and C, and fig. S1A). At 9 MO, however, AT8 ptau levels were significantly elevated in PS19 animals compared to wild type (WT), and significantly reduced in SORLA TG/PS19 hippocampus compared to PS19 (Fig. 1, B and C, and fig. S1A), with similar trending reductions in AT8 ptau in both SORLA TG/PS19 males and females (fig. S1A). At 3 MO, we observed little or no change in GFAP levels, an astrogliosis marker, in P301S tau animals; however, GFAP levels were significantly higher in 9 MO PS19 hippocampus and reduced in SORLA TG/PS19 animals (Fig. 1, B and C, and fig. S1A). Increased GFAP levels tightly correlated with AT8 ptau induction in 9 MO PS19 hippocampus (Fig. 1D and fig. S1B).

Next, we examined effects of SORLA up-regulation on histopathological changes in P301S tau brain. As reported by others (*34*), we observed ventricle enlargement in coronal sections in 9 MO PS19 animals, whereas SORLA up-regulation in PS19 animals largely suppressed ventricle dilation (Fig. 1, E and F, and fig. S1C). Hippocampal volume was also reduced in PS19 animals, and a trending increase in hippocampal volume was observed in SORLA TG/PS19 mice (fig. S1D). Moreover, SORLA up-regulation suppressed accumulation of AT8 ptau in PS19 hippocampus and featured increased thickness in the CA1 granule layer (Fig. 1G and fig. S1F). Consistent with the Western blot results, SORLA up-regulation also suppressed indicators of astrogliosis including increased GFAP staining and astrocyte hypertrophy in PS19 hippocampus (Fig. 1H). Together, these results indicate that SORLA up-regulation can suppress pathological changes in aged PS19 mouse brain.

### SORLA deletion exacerbates pathogenic changes in PS19 brain

**Fig. 2.**
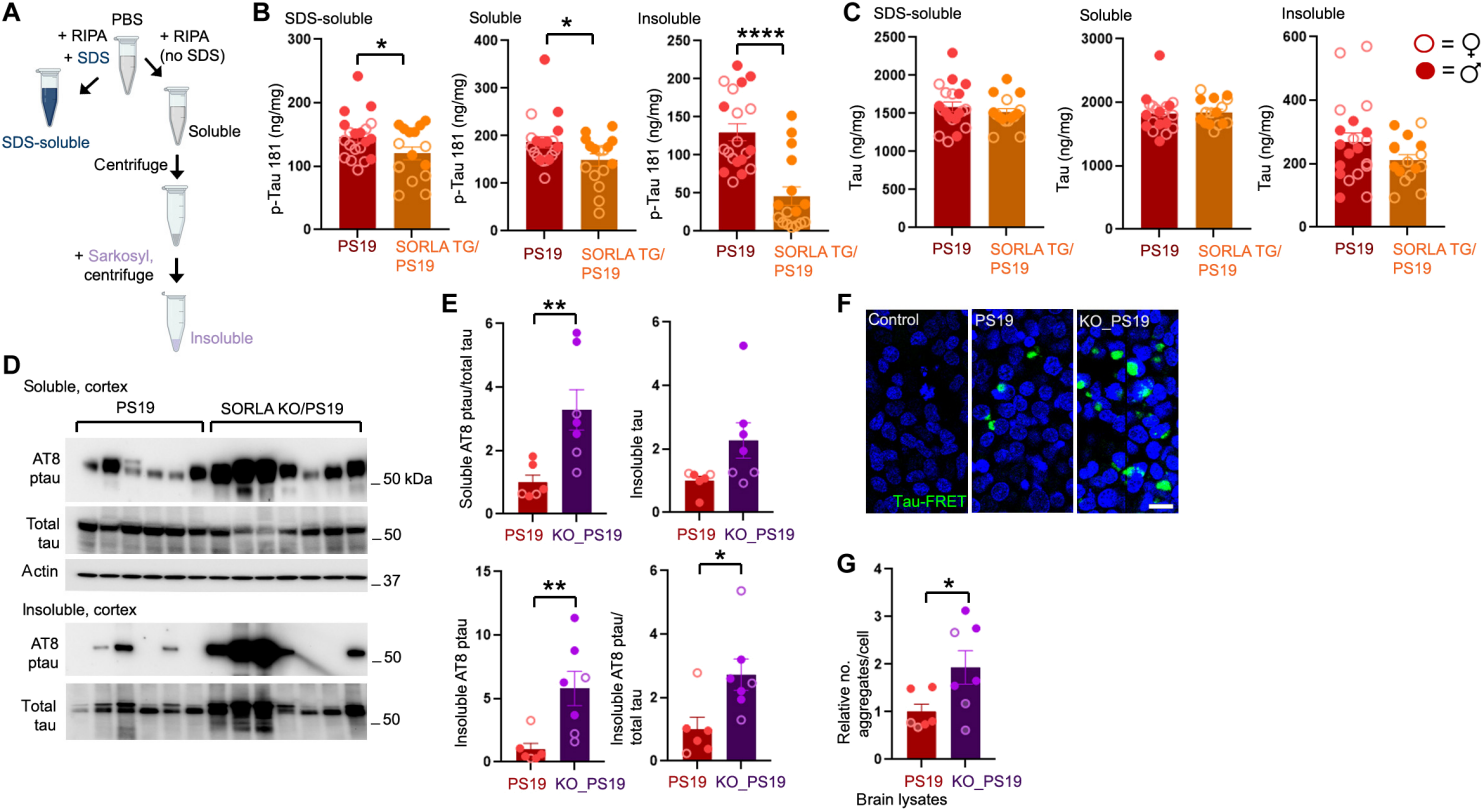
Comparing effects of SORLA up-regulation and deletion on tau solubility and seeding in PS19 brain. (**A**) Workflow schematic for extracting RIPA-SDS–soluble, RIPA-soluble, and Sarkosyl-insoluble tau. (**B** and **C**) Quantification of ptau 181 (B) and total tau (C) in RIPA-SDS–soluble, RIPA-soluble, and Sarkosyl-insoluble brain fractions as measured by ELISAs in 9 MO PS19 mice with or without SORLA overexpression. (**D**) Representative immunoblots to detect AT8 ptau and total tau in soluble and insoluble fractions from 7 MO PS19 and SORLA KO/PS19 (KO_PS19) cortex. (**E**) Graphs depict insoluble tau or AT8 ptau or soluble/insoluble AT8 ptau/total tau ratios normalized to PS19 (set to 1.0). (**F** and **G**) Cortical brain extracts were applied to HEK293 tau-RD cells and assayed for tau-FRET activity. Representative tau-FRET images (green) or DAPI (blue) are shown; scale bar, 20 μm. (G) Graph depicts relative number of tau-FRET aggregates from 7 MO PS19 and KO_PS19 extracts, normalized to PS19 (set to 1.0). Graphs depict mean ± SE, statistical significance was determined by unpaired Student’s *t* test [(B), (E), and (G)], *P < 0.05, **P < 0.01, ****P < 0.0001. Empty and filled plots represent female and male animals as indicated.

We first compared effects of SORLA modulation on soluble/insoluble tau and AT8 ptau levels in PS19 brain. We observed significant reduction of soluble AT8 ptau tau levels normalized over total tau in 11 MO SORLA TG/PS19 compared to PS19 cortex (fig. S2, A and B); however, trending decreases in insoluble tau and AT8 ptau and AT8 ptau/tau levels were observed in SORLA TG/PS19 over PS19 animals (fig. S2, A and B). Given that differences may be reduced between PS19 and SORLA TG/PS19 pathology at 11 months, we characterized ptau 181 (*41*) and total tau levels (*42*) in 9 MO PS19 mouse brain in soluble and insoluble extracts (Fig. 2, A to C, and fig. S2, C and D). We observed reduced ptau levels in soluble, SDS-soluble, and insoluble fractions, with no significant differences detected in total tau levels in PS19 compared to SORLA TG/PS19 extracts (Fig. 2, B and C).

Reduced SORLA levels have been reported in lymphoblasts (*25*) and the brain (*26*) of patients with AD, indicating that SORLA loss of function may be linked to AD pathogenesis. We generated homozygous SORLA KO mice (*Sorl1* deletion) in a PS19 background (“KO_PS19”) and compared effects of *Sorl1* deletion (SORLA KO) and SORLA up-regulation (SORLA TG) on tau pathology in PS19 mouse brain. In contrast to SORLA up-regulation, we found that both soluble and insoluble AT8 ptau/total tau levels were increased in 7 MO SORLA KO/PS19 (KO_PS19) cortex compared to PS19 alone (Fig. 2, D and E, and fig. S2E). We also compared effects of SORLA up-regulation and deletion on tau seeding activity using a human embryonic kidney (HEK) 293 tau-RD biosensor assay system (*43*). Using crude cortical lysates from 11 MO PS19 and SORLA TG/PS19 brain, we observed reduced tau seed–dependent Förster resonance energy transfer (FRET) activity in SORLA TG/PS19 compared to PS19 lysates in HEK tau-RD cells (fig. S2, F and G). In contrast to effects observed with SORLA TG/PS19 lysates, 7 MO SORLA KO/PS19 lysates showed enhanced tau seeding capacity compared to aged-matched PS19 cortex (Fig. 2, F and G, and fig. S2H).

SORLA has been previously shown to be an important regulator of endolysosomal trafficking, and down-regulation of endosomal retromer components has been observed in AD (*44*). SORLA deletion as well as AD-associated Y1816C, G511R, E270K, and Y141C variants can promote endosomal enlargement (*16*, *45*–*47*) and dysregulated trafficking of targets such as APP and GluA1 in iPSC-derived neurons (*48*). Given that extracellular tau can be internalized and trafficked to endosomes and lysosomes (*49*) and SORLA has been previously shown to be an important regulator of endolysosomal trafficking, we determined whether SORLA modulation can alter tau trafficking and uptake in cultured neurons. We observed significant enhancement of tau internalization in SORLA TG neurons compared to WT following 2 hours of exposure to tau oligomers (fig. S3, A and B) and a slight increase in lysosome size in SORLA TG neurons with exposure to tau oligomers (fig. S3, C and D). Combined with observations that SORLA can attenuate tau seeding, these results suggest that SORLA is likely enhancing extracellular tau uptake and delivery to lysosomes to limit cytosolic escape or seeding.

Together, our results indicate that SORLA up-regulation and deletion feature contrasting effects on insoluble tau and seed-competent tau in PS19 brain, and SORLA may enhance extracellular tau uptake and trafficking to lysosomes in neurons.

### SORLA up-regulation suppresses synaptic proteomic changes and drivers of pathological dysfunction in PS19 hippocampus

**Fig. 3.**
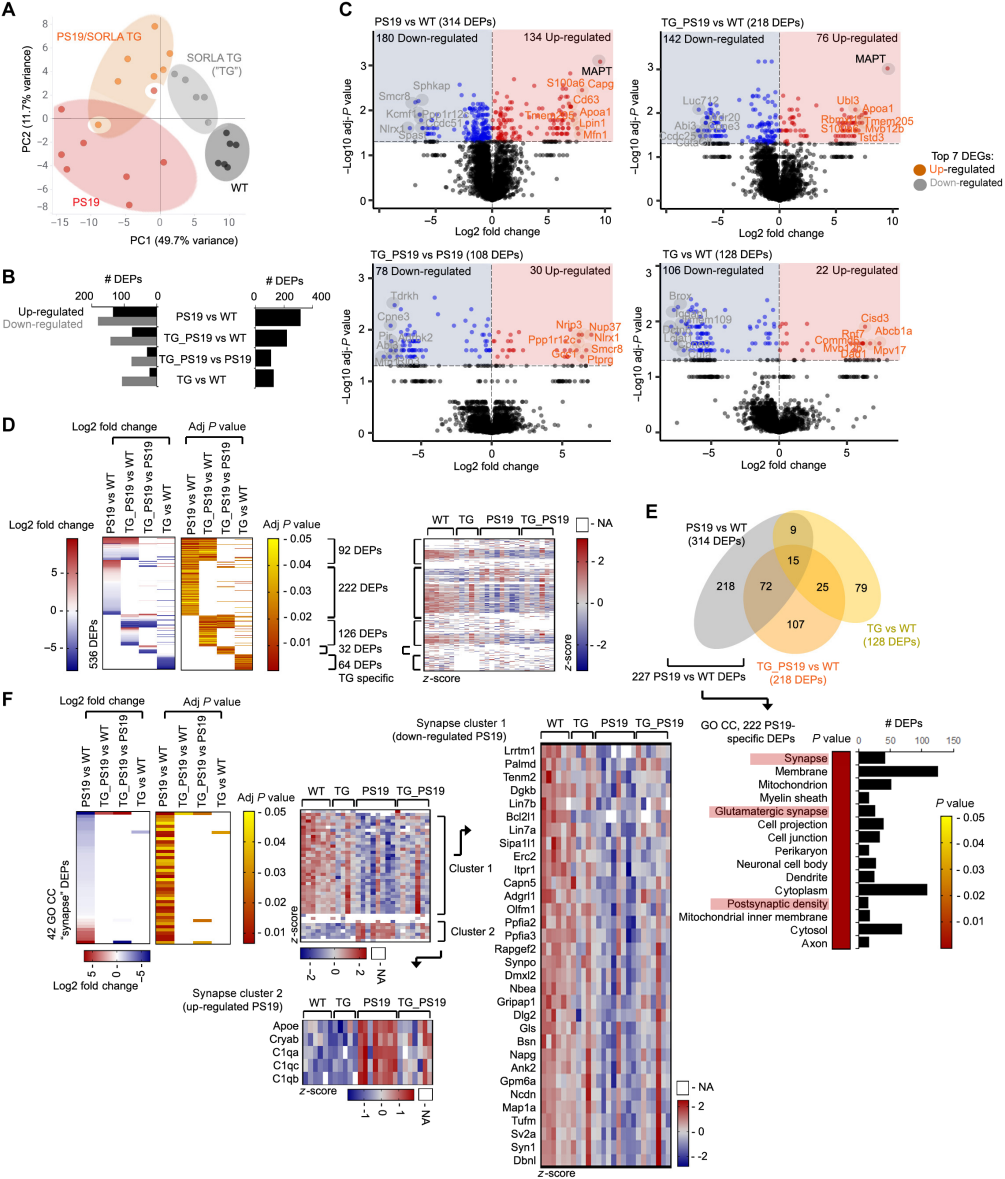
Proteomic changes in PS19 hippocampus are attenuated with SORLA up-regulation. (**A** to **C**) Label-free proteomic analysis of 9 MO mouse hippocampus. Protein lysates from WT (black), SORLA TG (“TG,” gray), PS19 (red), and SORLA TG/PS19 (“TG_PS19”) animals were processed for label-free proteomic analysis, and DEPs (adj *P* < 0.05) were subjected to PC analysis in (A). PCA clusters are highlighted in colored ovals. (B) Total number (right graph) and number of up-regulated (black bars) and down-regulated (gray bars) DEPs (left graph), and (C) volcano plots depicting Log2 fold change and adj *P* values in “PS19 versus WT,” “TG_PS19 versus WT,” “TG_PS19 versus PS19,” and “TG versus WT” hippocampus. The MAPT (human P301S tau) transgene (black) and top seven up-regulated (orange) or down-regulated (gray) DEPs are indicated. (**D**) Log2 fold change and adj *P* value in DEPs in comparisons indicated (left heatmaps), and *z*-score distribution for individual animals of the genotypes indicated (right heatmaps); “NA” values from missing peptide values are indicated in *z*-score heatmaps in white. (**E**) Venn diagram depicting overlap between “PS19 versus WT,” “TG_PS19 versus WT,” and “TG versus WT” DEPs. A total of 227 DEPs detected in PS19 versus WT (and absent in TG_PS19 versus WT) were subjected to GO analysis; *P* value (heatmap) and number of DEPs of top 15 GO CC categories (categories related to synaptic and neuronal structures highlighted) shown in the graph below. DEPs with opposing up- or down-regulation trends will count as unique DEPs in more than one comparison group; total DEPs may not match the sum of all Venn subgroups. (**F**) Log2 fold change and adj *P* value (left heatmaps) and *z*-score (right heatmaps) distribution of 42 GO CC “synapse”-related DEPs specific to PS19 versus WT comparisons. *z*-score distribution of GO CC “synapse” DEPs from (E) that feature down-regulation (cluster 1) or up-regulation in PS19 (cluster 2) is indicated.

We next determined whether SORLA up-regulation in PS19 animals could affect bulk proteomic alterations in PS19 mouse hippocampus. To this end, we performed label-free mass spectrometry analysis of protein lysates from 9 MO WT, SORLA TG (“TG”), PS19, and PS19/SORLA TG (“TG_PS19”) mouse hippocampus. Principal components analysis (PCA) revealed good clustering of replicate animals from each genotype (Fig. 3A), with the highest number of differentially expressed proteins (DEPs) identified when comparing PS19 versus WT hippocampus (Fig. 3, B and C; fig. S4, A to C; and table S1). A total of 227 DEPs (numbers adjusted for opposing DEP expression) were altered specifically in PS19 versus WT, and not in TG_PS19 versus WT comparisons (Fig. 3, D and E). TG_PS19 versus PS19 produced intermediate changes between PS19 versus WT and TG_PS19 versus WT comparisons, and shares a subset of DEPs with TG_PS19 versus WT, but not PS19 versus WT groups (Fig. 3D). We also observed few overlapping DEPs in TG_PS19 versus WT, TG_PS19 versus PS19, and TG versus WT comparisons (fig. S4, B to E), with some overlap between PS19 versus WT and TG_PS19 versus WT comparisons (fig. S4, C to E). These results indicate that DEPs induced by SORLA up-regulation are predominantly context specific to PS19 or WT mice, with partial overlap in the PS19 and SORLA TG/PS19 hippocampus.

We performed additional analysis on PS19 versus WT DEPs, in agreement with attenuated astrogliosis in SORLA TG/PS19 compared to PS19 hippocampus, and we observed induction of disease-associated astrocyte (DAA) DEPs such as Vim, Gfap, Gsn, and Aqp4 (*39*, *50*) in PS19, which was attenuated in SORLA TG/PS19 animals indicated by the *z*-score results (fig. S4F). Gene Ontology (GO) analysis of PS19 versus WT DEPs revealed enrichment of Kyoto Encyclopedia of Genes and Genomes (KEGG) “metabolic pathways” and “Alzheimer’s disease” categories (fig. S4, G to I), as well as GO CC categories related to “synapse” and “mitochondrion” (Fig. 3, E and F, and fig. S4J), with GO CC “synapse” (GO:0045202) as the top CC category enriched in PS19-specific DEPs (Fig. 3E and table S2). Of the 42 GO CC “synapse”-related DEPs, DEPs were down-regulated in PS19 compared to other genotypes in one cluster (cluster 1, Fig. 3F), whereas one cluster featured DEPs induced in PS19 hippocampus compared to other groups (cluster 2, Fig. 3F). We also performed GO analysis on PS19 versus WT or TG_PS19 versus WT DEPs and searched for enrichment in synaptic DEPs using SYNGO (syngoportal.org) (*51*). PS19 versus WT featured more synaptic DEPs compared to TG_PS19 versus WT (fig. S5A), and numerous GO Biological Process (BP) categories were identified and enriched in PS19 versus WT hippocampus related to the structure and function of synapses, including presynaptic and postsynaptic elements (fig. S5, A and B). These results suggest that proteins related to synaptic maintenance and function were altered in PS19 hippocampus, and potentially attenuated in SORLA TG/PS19 animals.

**Fig. 4.**
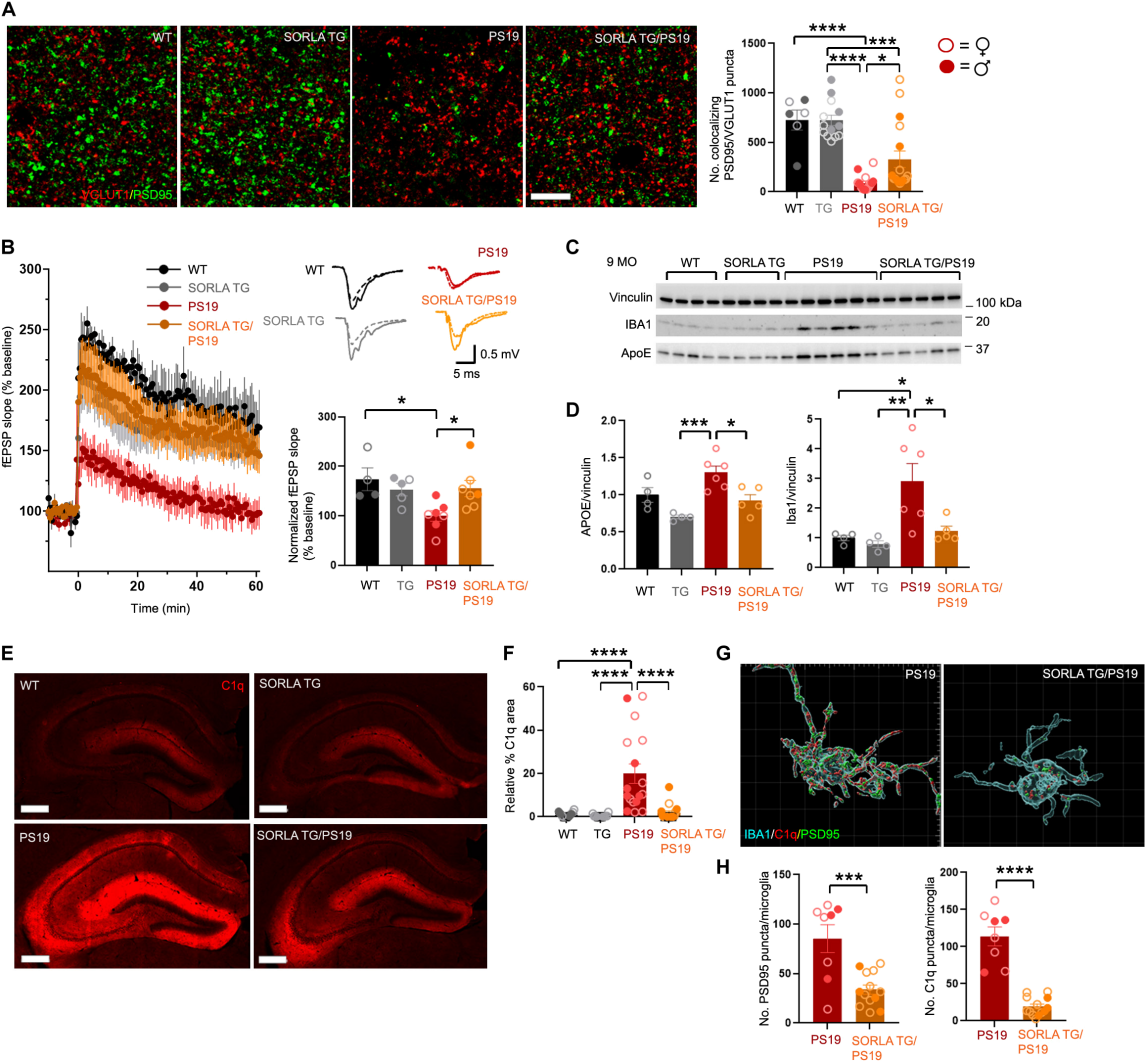
SORLA up-regulation attenuates synaptic impairment in PS19 hippocampus. (**A**) Representative staining images for VGLUT1 (red)/PSD95 (green) puncta in hippocampal sections from 9 MO animals; scale bar, 10 μm. Adjacent graph depicts the number of colocalizing PSD95/VGLUT1 puncta quantified using IMARIS. (**B**) SORLA up-regulation rescues LTP impairment in PS19 hippocampus. Acute slices from 6.5 to 7.5 MO animals were subjected to LTP induction (time 0) in the presence of 100 μM picrotoxin, and mean fEPSP slopes over baseline were quantified from recordings in the dentate gyrus (DG) region. Baseline measurements were recorded 20 min before and 60 min after stimulation. Adjacent fEPSP trace recordings (mean ± SE, averaged for each animal) before (solid lines) and after (dotted lines) LTP induction for each experimental genotype. Adjacent graph depicts cumulative fEPSP slopes during the last 10 min of recording (50 to 60 min after induction) averaged from individual animals of each genotype. (**C** and **D**) Representative ApoE, IBA1 immunoblots from hippocampal lysates of 9 MO animals and relative quantified band intensities were normalized to WT (set to 1.0). Only female mice were available for analyses. (**E**) Representative hippocampal sections from 9 MO animals stained for C1q (red); scale bar, 400 μm. (**F**) Graph depicts quantified percentage of C1q staining area normalized to WT (set to 1.0). (**G**) Representative reconstructed 3D confocal images of C1q (red) and PSD95 (green) puncta localized within microglia (blue) from hippocampal region of 9 MO animals. (**H**) Graphs depict quantification of PSD95 and C1q puncta internalized per microglia in PS19 or SORLA TG/PS19 hippocampus. All graphs depict mean ± SE. Statistical analysis was determined by two-way ANOVA then Tukey’s multiple comparisons in (A), (B), (D), and (F). Statistical significance was determined by unpaired Student’s *t* test in (H). *P < 0.05, **P < 0.01, ***P < 0.001, ****P < 0.0001. Empty and filled plots represent female and male animals as indicated.

Given that SORLA up-regulation can attenuate changes in expression of synaptic components in PS19 brain, we examined whether pre- and postsynaptic junctions were altered in PS19 and SORLA TG/PS19 hippocampus. In agreement with previous reports in PS19 animals (*34*, *52*), we observed decreased colocalization of PSD95 (postsynaptic)/VGLUT1 (presynaptic) puncta in 9 MO PS19 hippocampus compared to WT or SORLA TG, where SORLA TG/PS19 hippocampus featured partial restoration of colocalizing PSD95/VGLUT1 particles (Fig. 4A and fig. S5C). To further investigate effects of SORLA up-regulation on synaptic function, we compared LTP profiles in acute hippocampal slices from WT, SORLA TG, PS19, and SORLA TG/PS19 animals. Our results show that impaired LTP response in PS19 DG was partly restored in SORLA TG/PS19 animals (Fig. 4B and fig. S5D), demonstrating that SORLA up-regulation can partly restore functional synaptic impairment in PS19 hippocampus.

On the basis of two synapse-related DEP clusters (Fig. 3F), cluster 1 includes synaptic proteins, whereas in cluster 2, we observed robust up-regulation of synapse-related DEPs such as ApoE, Cryab, C1qa, C1qb, and C1qc in PS19, which was suppressed to levels comparable to WT or TG hippocampus in TG_PS19 animals (Fig. 3F). Given that deletion of *Apoe* (*38*) or *C1q* (*53*) can suppress degenerative features such as ventricular dilation in PS19 mouse brain, we validated effects of SORLA up-regulation on ApoE and C1q in PS19 animals by immunoblot and histological analysis. We found that ApoE (Fig. 4, C and D) and C1q (Fig. 4, E and F, and fig. S5E) were induced in 9 MO hippocampus and consequently normalized to near-WT levels in SORLA TG/PS19 animals (Fig. 4, D and F). Effects of SORLA up-regulation on ApoE appeared to be PS19 dependent; ApoE levels did not appear to differ significantly between SORLA TG and WT hippocampus in a non-PS19 background (Fig. 4D). We also observed tight correlation between AT8 ptau levels and ApoE in PS19 and SORLA TG/PS19 hippocampus (fig. S5F). Both ApoE and C1q are also biologically associated with synaptic function; C1q is a component of the complement pathway that accumulates at synapses to promote selective synaptic pruning during brain development (*54*–*56*) and mediates pathological elimination of synapses where microglia engulf or “prune” synapses (*57*). ApoE has been implicated in synaptic removal by colocalizing and enhancing Aβ accumulation at synapses (*58*), and APOE4 depletion in astrocytes suppresses microglial synaptic removal in an APOE4 KI model of mouse tauopathy (*59*). This indicates that SORLA modulates C1q and ApoE production in PS19 hippocampus, which in turn may directly or indirectly affect synaptic function. Because C1q is mainly produced in microglia, and elevations in PS19 microglial IBA1 expression are reduced in SORLA TG/PS19 brain (Fig. 4, C and D), we also explored whether microglial PSD95 and/or C1q uptake was altered with SORLA transgene expression (Fig. 4, G and H, and fig. S5G). Internalized C1q and PSD95 puncta in PS19 microglia was largely attenuated in SORLA TG/PS19 animals, further indicating that SORLA up-regulation can reverse pathological C1q and PSD95 uptake in microglia (Fig. 4, G and H, and fig. S5G).

It is reported that pharmacological retromer enhancement up-regulates SORLA and reduces Aβ and tau pathology in a 3xTg AD mouse model (*60*), and similarly decreases Aβ and ptau in iPSC-derived neurons with AD-associated SORLA variants (G511R, E270K, Y141C) or haploinsufficiency (*46*). As a component of the retromer complex, interactions between SORLA and the retromer complex may potentially affect tau pathology/pathogenesis in neurons and other cell types. Although down-regulation of retromer components such as VPS35, VPS26, and VPS29 has been observed in combined Aβ/tau (3xTg) secondary mouse models of tauopathy (*60*) as well as an in vitro tau seeding model (*61*), whether the retromer complex is dysregulated in primary tauopathy mouse models remain unclear. Our proteomic analysis of PS19 versus WT hippocampus failed to detect significant dysregulation of the core retromer components (table S1 and fig. S6A). Furthermore, we did not observe alterations in retromer components in our single-nucleus RNA sequencing (snRNA-seq) dataset in neurons or glia (table S3). Proteomics analyses from previous reports indicate that SORLA deletion in iPSC-derived astrocytes, neurons, or microglia had little effect on retromer components (*31*) (fig. S6B); similarly, we find little difference in retromer components (VPS35 and VPS26a) in WT compared to SORLA KO, PS19, or SORLA KO/PS19 brain (fig. S6, C and D). We characterized potential changes in the retromer complex and observed SORLA in high–molecular weight complexes (~660 kDa) by size exclusion chromatography fractionation in mouse brain, which appear to be unchanged in SORLA TG, PS19, and SORLA TG/PS19 mice, and VPS35/VPS26a in a complex of ~450 kDa in WT mouse brain with little to no variation in SORLA TG, KO, PS19, TG_PS19, or KO_PS19 backgrounds (fig. S6, E and F). We also observe no significant variation in SORLA or retromer levels (VPS35, VPS26a, and VPS26b) in cultured WT versus PS19 neurons (fig. S6, G and H). These results support the notion that SORLA and retromer may act independently in regulating tau pathology in vivo.

### Multi-omic analysis links proteomic changes in PS19 hippocampus to glial cells

**Fig. 5.**
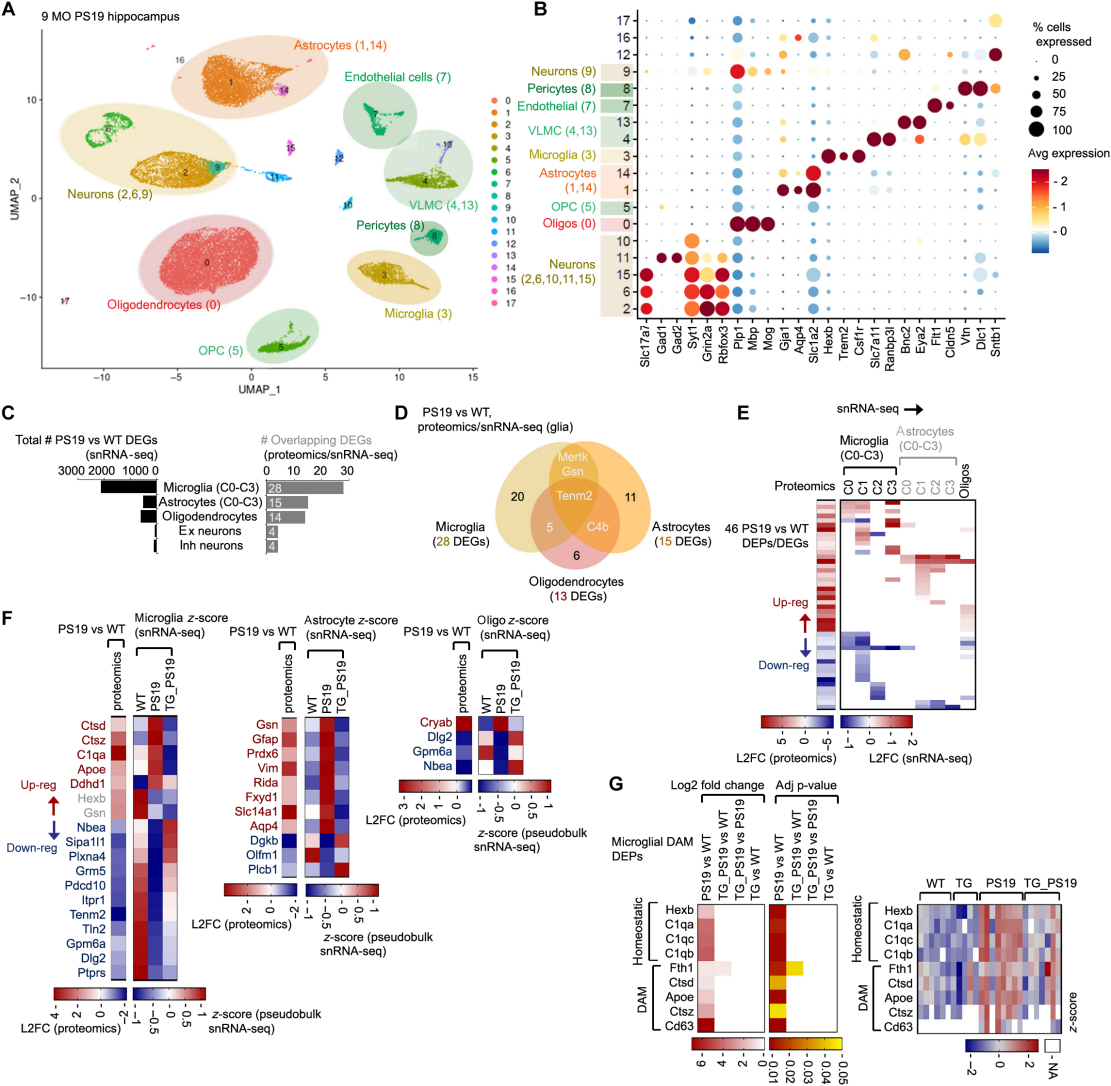
snRNA-seq and proteomic overlap analysis. (**A**) UMAP distribution of 29,438 nuclei clustered from 9 MO WT, PS19, and SORLA TG/PS19 hippocampus. Cell types identified according to alignment with murine reference profiles from the Allen Brain Atlas are indicated. (**B**) Dot plot depicting expression profiles of key identifying transcriptomic signatures from each cluster from (A), heat scales for expression levels, as well as % expression (dot size) are shown. (**C** to **F**) Mapping bulk proteomic expression profiles to cell-specific changes in transcript expression. (C) Graph showing total number of PS19 versus WT DEGs identified in all glia/neuron clusters (black bars), and number of PS19 versus WT DEGs overlapping with PS19 versus WT DEPs for each cell type (gray bars). (D) Venn diagram indicating shared PS19 versus WT DEPs/DEGs in glia. (E) Heatmap depicting 46 up-regulated/down-regulated PS19 versus WT DEPs with matching PS19 versus WT DEG profiles in glia cell clusters indicated. (F) Attenuation of PS19 versus WT DEP and DEG profiles with SORLA up-regulation in glia. Heatmaps comparing PS19 versus WT DEP profiles (left heatmaps) and pseudobulk *z*-score expression profiles (right heatmaps) in microglia, astrocytes, and oligodendrocytes as indicated. mRNA expression for Hexb/Gsn in microglia (gray) did not match PS19 versus WT up-regulation by proteomic analysis; rather, expression of astrocyte Gsn transcripts matched proteomic Gsn PS19 versus WT profiles. (**G**) Log2 fold change/adj *P* value and *z*-score distribution of DAM-related DEPs identified in 9 MO mouse hippocampus by label-free proteomic analysis.

Our results indicate that SORLA up-regulation can attenuate pathological effects in aged PS19 brain. To explore whether these restorative effects correlate with cell-specific transcriptomic changes, we subjected hippocampal tissue from 9 MO WT, PS19, and SORLA TG/PS19 (“TG_PS19”) to snRNA-seq analysis (Fig. 5A). We sequenced a total of 32,048 single nuclei, clustered into 18 main clusters of varying cell identity (Fig. 5B and fig. S7, A and B), retaining 28,348 high-quality single-nuclei data after doublet removal and quality control verification. We determined cell cluster identities by expression of cell-type markers, as well as reference mapping to the Allen Brain Cell Atlas. As expected, we did not detect human SORLA (*SORL1*) expression in WT and PS19 samples, and we observed up-regulation of human SORLA (*SORL1*) in most cell types in SORLA TG/PS19 hippocampus (fig. S7, C and E). Murine *Sorl1* was also detected in most central nervous system (CNS) cell types (fig. S7, D and E) especially in neurons.

We then mapped proteomic changes in PS19 versus WT hippocampus to mRNA expression profiles in glial and neuronal cell types (Fig. 5, C to E). A majority of PS19 versus WT DEPs mapped to glial DEGs (Fig. 5, C to E); only three PS19 versus WT DEPs (Slc4a4, Dgkb, and Tenm2) matched snRNA-seq profiles in neurons (fig. S8, A and B). We identified a total of 46 glial PS19 versus WT DEPs to glial DEGs in various subclusters (Fig. 5E and fig. S8C). Given that PS19 versus WT proteomic DEP profiles were attenuated with SORLA up-regulation, we also determined whether PS19 versus WT DEGs trends were also reversed/attenuated in the 46 DEPs/DEGs including ApoE and C1qa in SORLA TG/PS19 hippocampus (Fig. 5F and fig. S8C). We largely observed attenuated/reversed PS19 versus WT pseudobulk mRNA expression trends in SORLA TG/PS19 hippocampus with the exception of Hexb in microglia (Fig. 5F and fig. S8C). Gsn DEP expression profiles potentially mapped to both microglia (cluster 3) and astrocytes (cluster 1); however, Gsn DEP expression profiles appeared to match astrocyte pseudobulk transcripts rather than microglia mRNA (Fig. 5F). Similar to reduced attenuation of PS19 DAA DEPs in SORLA TG/PS19 hippocampus (fig. S4F), we also observed induction of disease-associated microglia (DAM) (*62*, *63*) DEPs in PS19 hippocampus, which were attenuated in SORLA TG/PS19 animals (Fig. 5G). We also confirmed induction of PS19 *Ctsd*, *Ctsz*, *Apoe*, and *C1qa* DAM transcripts and reduction of these DEGs in microglia (Fig. 5F).

Given the importance of neurons in tau pathology, together with a likely role for SORLA in influencing neuronal tau pathology and seeding phenotypes described so far, we explored effects of SORLA up-regulation on neuronal transcriptomic profiles in PS19 hippocampus. To this end, we characterized snRNA-seq profiles from reclustered neuronal nuclei (clusters 2, 6, 10, 11, and 15, Fig. 5, A and B) in WT, PS19, and SORLA TG/PS19 hippocampus. Neuronal snRNA-seq profiles segregated into 13 defined clusters (fig. S9, A to C), comprising neurons expressing excitatory cell markers such as *Slc17a7* and inhibitory neuronal markers (*Gad1* and *Gad2*) (fig. S9D). Clusters 0 and 1 comprised the majority of neuronal DEGs, with markedly fewer DEGs observed in clusters 2 through 6 (table S3); interestingly, a large proportion of PS19 DEGs in both excitatory and inhibitory neurons were reversed in TG_PS19 neurons (fig. S9, E to H). We observed a similar number of DEGs in PS19 versus WT and TG_PS19 versus PS19 comparisons in excitatory and inhibitory neurons (fig. S9E), and a large number of DEGs were shared between excitatory and inhibitory neurons (fig. S9, E and F). SORLA up-regulation largely reversed neuronal GO CC components related to “glutamatergic synapse” DEGs (fig. S10, A and B), as well as other components related to neurons, including DEGs related to GO CC “neuron projection,” “dendrite,” and “axons” (fig. S10A). GO analysis of all DEGs detected in all neuronal subclusters revealed enrichment of DEGs in KEGG pathways such as “Long-term potentiation,” “Glutamatergic synapse” (fig. S10, C and D), and GO MF (Molecular Function) pathways such as “amyloid binding” (fig. S10E). DEG profiles in these categories differed between PS19 versus WT, and TG_PS19 versus PS19 hippocampus, such as up-regulation of AMPAR subunits (*Gria1* and *Gria2*) in cluster 0 PS19 versus WT neurons (fig. S10, D and E). Together, these results indicate that SORLA up-regulation can reverse pathological signatures, including synapse-related genes in PS19 neurons.

### SORLA up-regulation attenuates microglia activation and pathological transcriptomic profiles in PS19 hippocampus

**Fig. 6.**
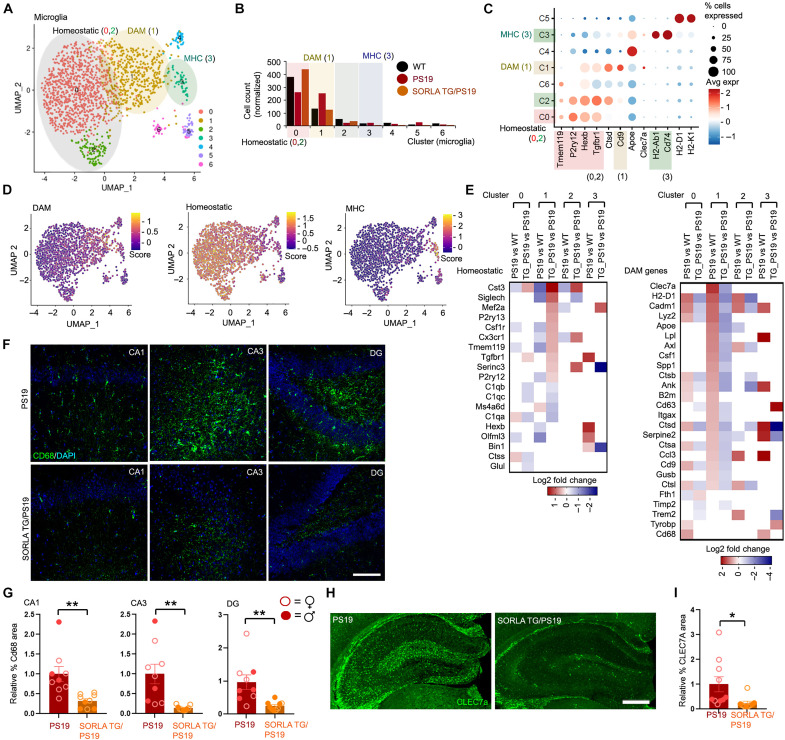
SORLA up-regulation suppresses microglia activation and pathological microglia signatures in PS19 hippocampus. (**A**) UMAP distribution of reclustered microglia nuclei. Homeostatic (0, 2), DAM (1), and MHC (3) clusters are indicated. (**B**) Graph depicting normalized cell numbers in WT, PS19, and SORLA TG/PS19 microglia clusters. (**C**) Dot plot depicting expression of microglia gene signatures in microglia clusters 0 through 6. (**D**) Heat-scaled UMAP depicting expression of homeostatic, DAM, and MHC genes within reclustered microglia; color scale represents enrichment of genes within each category using scores generated. (**E**) Microglia homeostatic/DAM DEGs and their expression profiles. Log2 fold change (left heatmaps) of DEGs (*P* < 0.05, Log2 fold change >0.3, <−0.3) associated with homeostatic (left heatmap) and DAM microglia (right heatmap) in PS19 versus WT and TG_PS19 versus PS19 comparisons are shown. (**F**) Representative images of 9 MO brain slices stained with CD68 (green) or nuclei (DAPI, blue); scale bar, 100 μm. Images were obtained from CA1, CA3, and DG regions as indicated. (**G**) Graphs depicting quantified percentage CD68 staining area from PS19 and SORLA TG/PS19 animals is shown from imaged regions in (F). (**H**) Representative image of 9 MO PS19 and SORLA TG/PS19 hippocampus stained for CLEC7A (green); scale bar, 500 μm. (**I**) Graph depicts % CLEC7A staining area in hippocampus quantified from representative images. All graphs depict mean ± SE. Statistical significance was determined by unpaired Student’s *t* test in (G) and (I), *P < 0.05, **P < 0.01. Empty and filled plots represent female and male animals as indicated.

To characterize effects of SORLA up-regulation on microglia snRNA-seq profiles, we reclustered nuclei from microglia (cluster 3) (Fig. 5A) and compared expression profiles between WT, PS19, and TG_PS19 groups. Transcriptomic profiles from reclustered microglia nuclei segregated into seven clusters (Fig. 6, A to C, and fig. S11A), with distinct separation of homeostatic (clusters 0 and 2), DAM (cluster 1), and MHC (cluster 3) clusters (Fig. 6, A to D). Homeostatic DEGs that were largely down-regulated in PS19 DEGs (PS19 versus WT) such as *Cst3*, *Siglech*, *Mef2a*, *Csf1r*, *Cx3cr1*, *Tmem119*, and *P2ry12* were up-regulated in SORLA TG/PS19 animals (TG_PS19 versus PS19) (Fig. 6E and fig. S11, B and C). Trends with homeostatic DEGs were most evident in DAM cluster 1, while DAM-associated genes such as *Clec7a*, *H2-D1*, *Cadm1*, *Lyz2*, *Apoe*, *Lpl*, *Axl*, and *Csf1* up-regulated in PS19 microglia were down-regulated in SORLA TG/PS19 animals most abundantly in cluster 1 (Fig. 6E and fig. S12C). In agreement with our proteomics analysis, we also observed up-regulation of *C1qa* in PS19, which was reduced in SORLA TG/PS19 hippocampus (cluster 0, Fig. 6E). We also observed reversed homeostatic and DAM expression gene trends in PS19 and SORLA TG/PS19 microglia in homeostatic clusters 0 and 2 (Fig. 6E), and up-regulation of MHC-associated genes in PS19 microglia were also reversed in SORLA TG/PS19 microglia in clusters 0 to 3 (fig. S11D).

In addition to DEGs associated with homeostatic, DAM, and MHC signatures, we observed that SORLA up-regulation can reverse differentially expressed genes (DEGs) in clusters 0 through 3 (fig. S11, E and F). GO analysis of pathological PS19 DEGs restored in SORLA TG/PS19 microglia include DEGs associated with KEGG pathways related to “Lysosome” and “Phagosome” (fig. S11, G and H). KEGG lysosome-associated PS19 microglia DEGs were largely up-regulated and consequently down-regulated in SORLA TG/PS19 microglia (fig. S11, G and H), indicating that SORLA up-regulation can potentially normalize pathological elevation of lysosome components in PS19 microglia. Given that lysosomal genes were up-regulated in PS19 microglia and down-regulated in SORLA TG/PS19 animals in vivo, we tested whether SORLA up-regulation could affect tau uptake and endolysosomal trafficking in cultured microglia. We found that 2 hours postexposure to tau oligomers, SORLA TG microglia exhibited enhanced tau uptake compared to WT (fig. S12, A and B). We also observed a trending enhancement of tau colocalization with early endosomes (EEA1), and significantly enhanced tau colocalization with late endosomes (Rab7) and lysosomes (LAMP1) in SORLA TG microglia (fig. S12, A and B). This suggests that SORLA up-regulation can enhance uptake of tau oligomers in vitro and promote tau trafficking to microglial endolysosomal compartments.

Given that multi-omic mapping of proteomic and snRNA-seq profiles suggested SORLA overexpression mainly reversed overlapping protein/mRNA expression in glia (Fig. 5F), we determined whether SORLA up-regulation suppresses markers of microglia activation such as CD68. CD68, IBA1 (Fig. 6, F and G, and fig. S12, C to E), and CLEC7A (Fig. 6, H and I, and fig. S12F) levels in 9 MO SORLA TG/PS19 hippocampus were significantly reduced compared to PS19. In addition, we observed down-regulation of the microglia homeostatic marker P2Y12 in PS19 hippocampus, which was restored in SORLA TG/PS19 animals (fig. S12, G and H). Thus, SORLA up-regulation can suppress induction of markers associated with microglia activation in PS19 hippocampus.

### SORLA up-regulation attenuates pathological transcriptomic profiles in PS19 astrocytes and oligodendrocytes

**Fig. 7.**
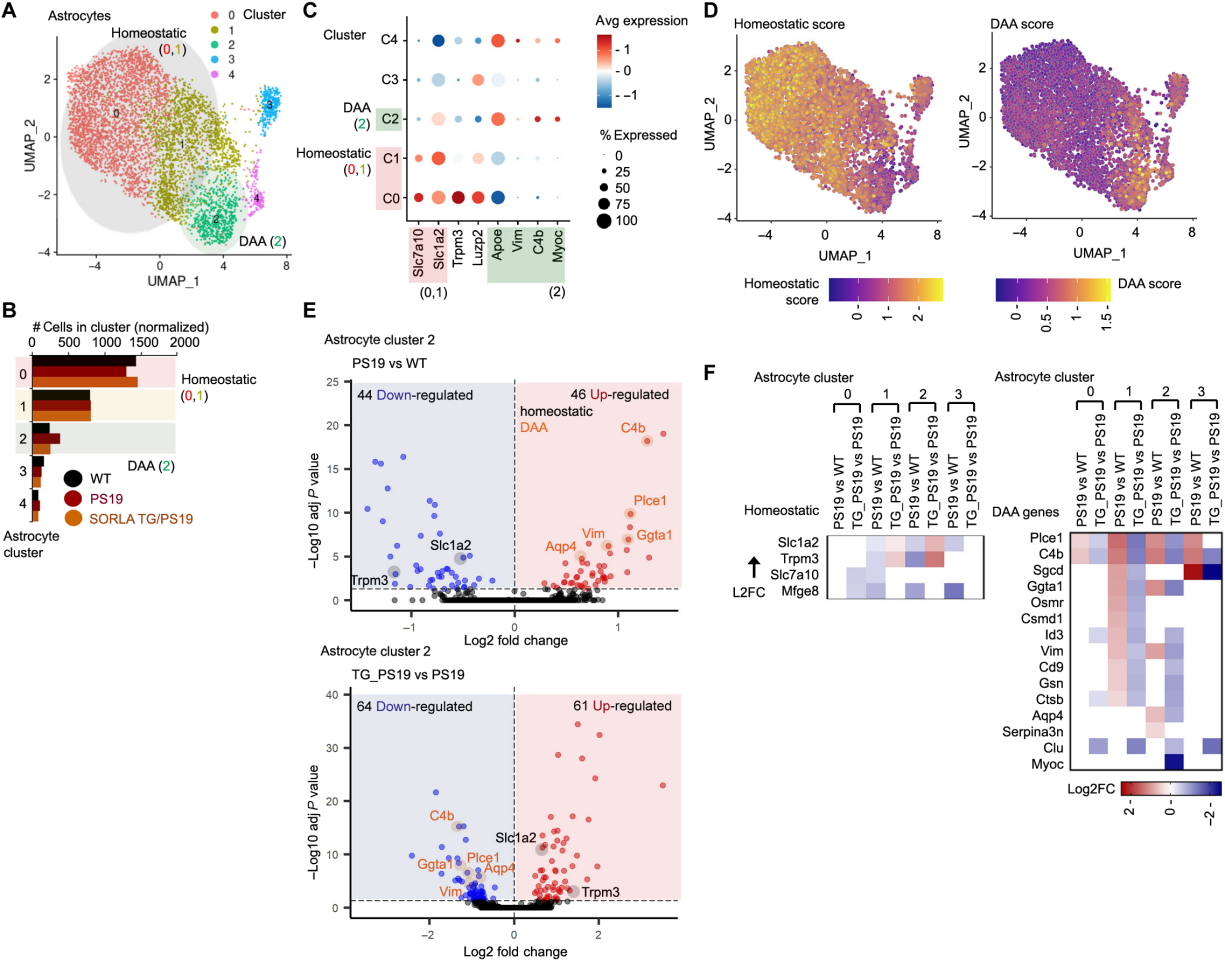
SORLA up-regulation suppresses pathological astrocyte signatures in PS19 hippocampus. (**A**) UMAP distribution of 5579 reclustered astrocyte nuclei. (**B**) Number of DEGs identified in each astrocyte cluster in PS19 versus WT and TG_PS19 versus PS19 comparisons. (**C**) Dot plot depicting expression profiles of key astrocyte signatures from each cluster from (A), heat scales for expression levels, as well as % expression (dot size) are shown. (**D**) Expression of homeostatic and DAA astrocyte genes within reclustered astrocyte transcription profiles. Heat-scaled UMAP depicts homeostatic and DAA gene expression scores for reclustered astrocyte nuclei. (**E**) Volcano plots depicting up- and down-regulated DEGs in PS19 versus WT and TG_PS19 versus PS19 comparisons in astrocyte cluster 2 (adj *P* value <0.05). Homeostatic (black) and DAA (orange) DEGs featuring opposing expression profiles in PS19 and TG_PS19 astrocytes are indicated. (**F**) Expression of homeostatic (left heatmaps) and DAA (right heatmaps) in PS19 versus WT and TG_PS19 versus PS19 comparisons; Log2 fold change (red/blue) expression changes (adj *P* < 0.05) are shown for comparisons in the clusters indicated.

Reclustered astrocyte snRNA-seq profiles revealed five clusters (clusters 0 through 4) with the highest number of DEGs in cluster 1 (Fig. 7, A to C, and fig. S13, A and B), and DAAs residing primarily in cluster 2 (Fig. 7, B to D, and fig. S13, B to D). PS19 astrocytes featured increased numbers of DAA cluster 2 astrocytes, which were reduced in TG_PS19 hippocampus (Fig. 7B and fig. S13, C, F, and G). Although expression of DAA genes such as *C4b*, *Vim*, *Plce1*, *Aqp4*, *Ggta1*, and *Serpina3n* was primarily enriched in cluster 2 (Fig. 7, E and F), up-regulated DAA signatures in PS19 astrocytes were largely reversed in TG_PS19 hippocampus in clusters 0 through 3 (Fig. 7, E and F, and fig. S13, E to G). Similar to our observations in microglia, SORLA up-regulation largely reversed pathological DEG profiles in PS19 astrocytes (fig. S13, F and G). We also characterized bulk changes from the main oligodendrocyte UMAP cluster (cluster 0) (Fig. 5A) and observed that a large proportion of PS19 oligodendrocyte DEGs were reversed in SORLA TG/PS19 hippocampus (fig. S13, H and I). Pathological changes in disease-associated (*64*) DEGs in PS19 oligodendrocytes such as *Cd63*, *Cd9*, *H2-D1*, *Sgk1*, and *C4b* were reversed in SORLA TG/PS19 hippocampus (fig. S13J). Together, these results indicate that global SORLA up-regulation can suppress pathological glial activation in PS19 hippocampus.

### Pathological alterations in cell-cell communication/signaling networks are attenuated with SORLA up-regulation

**Fig. 8.**
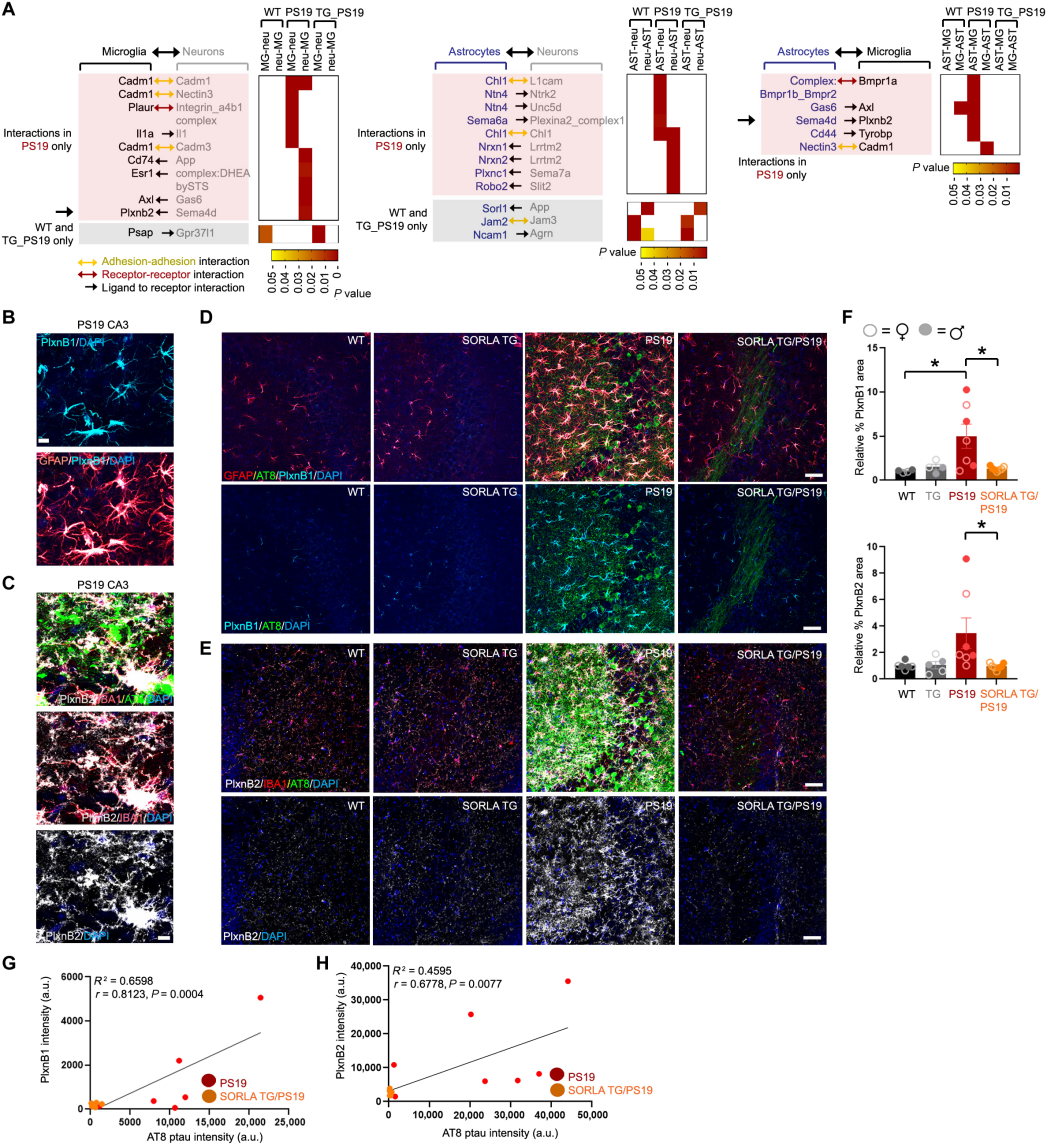
Characterizing changes in receptor/ligand pairs in PS19 hippocampus with SORLA up-regulation. (**A**) Identification of changes in ligand/receptor pairs in microglia/neurons (left), astrocytes/neurons (middle), and astrocytes/microglia (right) using CellPhoneDB analysis of snRNA-seq profiles in WT, PS19, and SORLA TG/PS19 (TG_PS19) hippocampus. Changes in matched receptor/ligand pairs specific to PS19 are highlighted in red; paired interactions specific to WT and TG_PS19 hippocampus are highlighted in gray. Adjacent heatmaps depict *P* values calculated for each receptor/ligand interaction (replicate *P* values are averaged) in paired cell types. (**B**) Magnified images for PlxnB1(cyan) and GFAP (red) staining; scale bar, 10 μm. Colocalization of PlxnB1 (cyan) and GFAP (red) in PS19 hippocampus (CA3); PlxnB1/GFAP overlap is indicated in white in the bottom panel; scale bar, 10 μm. (**C**) Magnified images for PlxnB2 (white) and IBA1 (red) staining; scale bar, 10 μm. Colocalization of PlxnB2 (white), IBA1 (red), and AT8 p-tau (green) (top panel) in PS19 hippocampus (CA3). Merged PlxnB2/IBA1 (middle) and PlxnB2 alone (bottom panel) are shown; scale bar, 10 μm. (**D**) Representative images of PlxnB1 (cyan), AT8 ptau (green), GFAP (red), and DAPI (blue) in 9 MO CA3 hippocampus; scale bar, 50 μm. (**E**) Representative images of PlxnB2 (white), IBA1 (red), AT8 ptau (green), and DAPI (blue) in 9 MO CA3 hippocampus; scale bar, 50 μm. (**F**) Quantification of relative PlxnB1 and PlxnB2 staining area from 9 MO WT, SORLA TG, PS19, and SORLA TG/PS19 hippocampus (CA3). (**G** and **H**) Correlation between PlxnB1 (G) or PlxnB2 (H) and AT8 ptau staining intensity (arbitrary units, a.u.) in PS19 (red) and SORLA TG/PS19 animals (orange). Coefficient of determination (*R*^2^), Pearson correlation coefficient (*r*), and *P* value are shown. All graphs depict mean ± SE. Statistical analysis was determined by two-way ANOVA then Tukey’s multiple comparisons in (F). *P < 0.05, **P < 0.01. Empty and filled plots represent female and male animals as indicated.

Given that SORLA up-regulation can reverse transcriptomic changes in various cell types in PS19 hippocampus, we explored whether specific cell-cell interaction networks could be potentially restored in SORLA TG/PS19 animals. Using the CellPhoneDB database (v5.0.0) to infer cell-cell communication networks from our snRNA-seq analyses (*65*), we identified ligand/receptor complexes enriched in PS19 (pathogenic) or WT/TG_PS19 (nonpathogenic) genotypes (Fig. 8A and fig. S14A). As expected, we also observed enrichment of SORLA (*Sorl1*)/APP cell-cell interaction pairs in WT and TG_PS19 astrocyte/neurons (Fig. 8A). Interactions featured ligand/receptor pairs associated with cell adhesion (*Jam3*, *Integrin aMb2*, *Cadm1*, *Chl1*, *L1cam*, *Nrxn1*, and *Nrxn2*), phagocytosis (*Axl* and *Gas6*), antigen presentation (*Cd74*and *Cd44*), and inflammation (*Sema4d*, *Sema6a*, and *Plxnb2*).

We observed enrichment of semaphorin signaling ligands (*Sema6a*—astrocytes, *Sema4d*—neurons, astrocytes) and Semaphorin receptors (*Plexina2*—neurons, *Plxnb2*—microglia) specifically in PS19 hippocampus (Fig. 8A). The Semaphorin protein family comprises an extracellular Sema domain as well as a Plexin-Semaphorin-Integrin domain required for mediating homotypic and heterotypic interactions (*66*, *67*). Both PlxnB1 and PlxnB2 are receptors for class 4 semaphorin ligands such as Sema4D (*68*); PlxnB1/Sema4D have been previously shown to mediate growth cone collapse in neuronal axonal guidance during early brain development (*69*), as well as suppression of bone mass and osteoblast differentiation (*70*), while PlxnB2/Sema4D interactions are important in epithelial repair response (*71*). Sema4D levels are up-regulated in human Huntington’s disease (HD) and AD brain (*72*), while expression of the Sema4D receptor, PlxnB1, correlates with Aβ pathology and cognitive impairment in AD (*73*). Because antibody-based Sema4D targeting is shown to reverse cognitive impairment and astrogliosis in an Aβ AD (APPSwDI/NOS2^−/−^) mouse model (*72*), we explored whether Sema4D and cognate PlxnB1 and PlxnB2 receptors were altered in PS19 hippocampus.

*Sema4d* expression is enriched in mouse oligodendrocytes, microglia, astrocyte, and neurons compared to other brain cell types (*74*, *75*). We observed a variable, but slight increase in Sema4D levels in PS19 hippocampus by immunoblot, and comparatively reduced Sema4D levels in SORLA TG/PS19 animals (fig. S14, B and C). PlxnB1 binds Sema4D with high affinity (*76*) and is highly expressed in mouse astrocytes (*74*), whereas PlxnB2 is expressed primarily in microglia (*74*). We observed high expression of PlxnB1 in astrocytes (Fig. 8B) and PlxnB2 in microglia (Fig. 8C). Basal PlxnB1 and PlxnB2 levels were quite low in WT and SORLA TG hippocampus (CA3), and markedly increased in PS19 animals (Fig. 8, D to F). SORLA up-regulation significantly suppressed PlxnB1 and PlxnB2 levels in PS19 hippocampus (Fig. 8, D to F). SORLA TG/PS19 animals featured significantly less AT8 ptau staining consistent with our results above, and although PlxnB1 or B2 and AT8 ptau intensity varied between PS19 animals (Fig. 8, F to H), higher PlxnB1 or B2 staining intensity generally corresponded to enhanced AT8 ptau levels (Fig. 8, G and H). These results indicate that PlxnB1 and B2 levels correlate with tau pathology in PS19 mouse brain.

To further characterize whether PlxnB1/B2 and Sema4D interactions correlate with disease pathology in AD brain, we determined whether cell-specific expression of *PLXNB1/B2* and *SEMA4D* interaction pairs was enriched in human AD brain. We searched specifically for expression of *SEM4D/PLXN* interaction pairs in astrocytes, neurons, and microglia in snRNA-seq datasets from dorsal lateral prefrontal cortex in individuals with little or no amyloid and tau tangle pathology, or individuals with amyloid and varying levels of tau pathology (*77*). We observed enrichment of *SEMA4D/PLXNB2* in astrocyte/neuron pairs, as well as microglia/neuron pairs in human AD brain characterized using CellPhoneDB; no *SEMA4D/PLXNB2* ligand/receptor pairs were identified from these cell types in non-AD controls (fig. S14D). We identified *SEMA4D/PLXNB1* and *B2* expression in astrocyte/microglia cell-cell interaction pairs in control brain, and these interactions were more abundant in AD brain (fig. S14D). Together, these results indicate that *SEMA4D/PLXNB* interactions are enhanced in specific neuron/glia cell-cell interaction pairs in human AD, and SORLA up-regulation can alter pathological Sema4D/PlxnB interaction in PS19 hippocampus.

### Characterizing effects of SORLA deletion on neurons and glia in PS19 hippocampus

Having characterized contrasting effects of SORLA up-regulation and deletion on tau pathology, we then investigated whether SORLA deletion altered neuronal and glial transcripts in PS19 hippocampus. To this end, we first sampled regions of interest (ROIs) in CA1, CA3, and DG subregions within the hippocampus in 9 MO WT, PS19, KO and KO_PS19 animals (fig. S15A), and characterized transcriptomic changes associated with NeuN-stained neurons (fig. S15, A to E). We observed a higher number of neuronal DEGs in KO_PS19 versus WT (9627 DEGs) compared to PS19 versus WT (3682 DEGs) (fig. S15, B and C), suggesting that SORLA deletion may potentially further alter changes in PS19 neurons. GO KEGG pathways enriched in the neuronal DEGs revealed “pathways of neurodegeneration” as the top KEGG pathway (fig. S15D). Log2 fold change and *z*-score distribution of 315 DEGs within the KEGG “pathways of neurodegeneration” category revealed notable changes in PS19 over WT and KO neurons; changes in PS19 neurons were further enhanced in KO_PS19 hippocampus (fig. S15E). This suggests that SORLA deletion may enhance pathological changes in PS19 neurons.

**Fig. 9.**
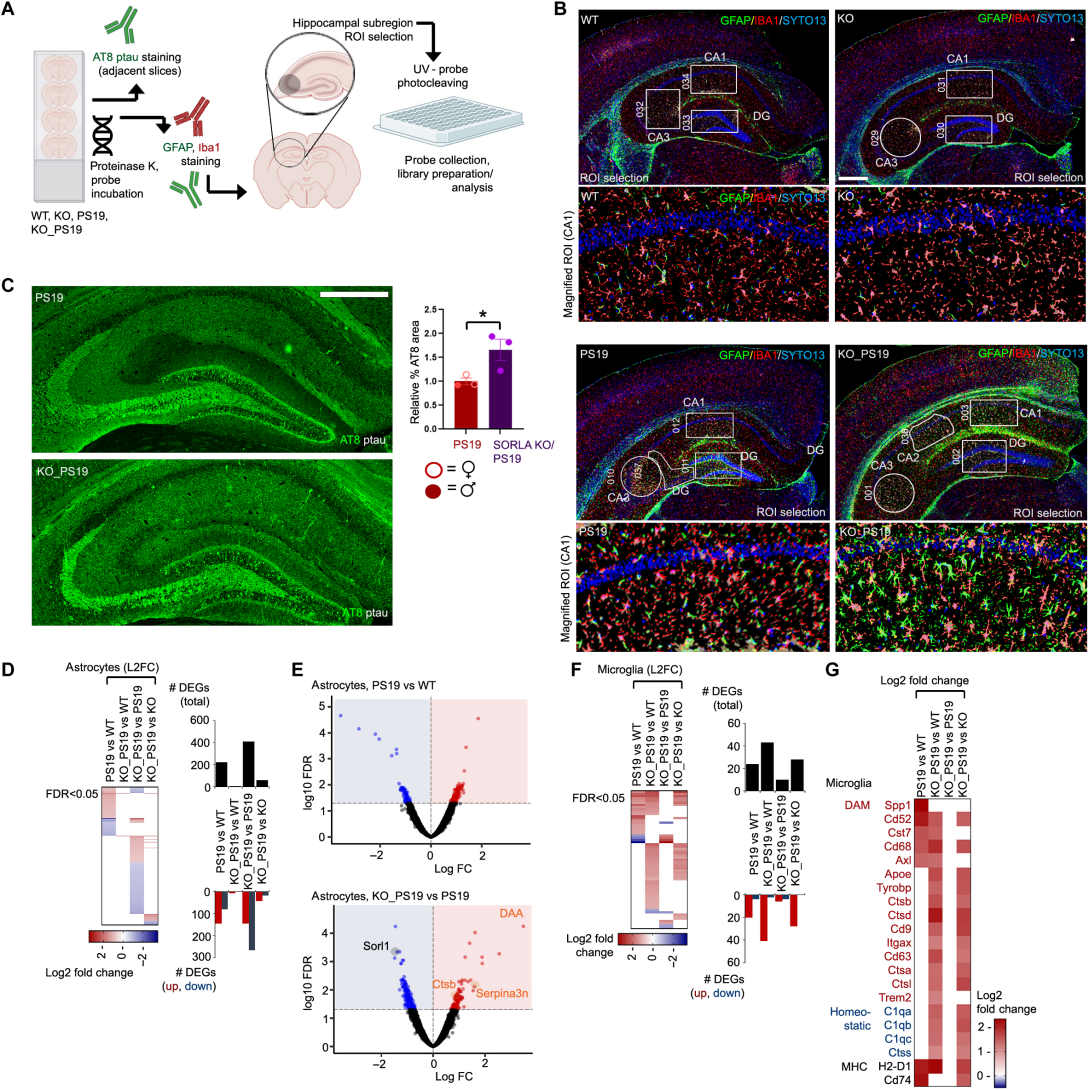
SORLA deletion exacerbates astrocyte and microglia activation in PS19 hippocampus. (**A**) Workflow schematic for hippocampal processing and GeoMx analysis. Image objects (slides, brain sections, antibodies, and plate) were created using Biorender [“GeoMx” by Huang, T. (2026), https://BioRender.com/ojq7jew]. (**B**) Representative ROI images of 9 MO WT and SORLA KO (“KO”), PS19, and SORLA KO/PS19 (“KO_PS19”) hippocampus stained with GFAP (green), IBA1 (red), and nuclei (SYTO13, blue) for GeoMx analysis. Magnified representative images of GeoMx UV-cleaved ROIs are shown (CA1 region); GFAP (green) and Iba1 (red) segmentation are shown based on thresholding for GeoMx UV-cleavage, and numerical GeoMx ROI annotations for subhippocampal regions are indicated. Aggregate ROIs were used for DEG analysis, and individual expression trends within subregions for annotated cell types are included in table S4. Scale bar, 500 μm. (**C**) Representative AT8 ptau staining (green) for PS19 and KO_PS19 hippocampus on adjacent brain slices. Graph depicts relative percentage of AT8 ptau staining area for PS19 and SORLA KO/PS19 brain sections. Scale bar, 500 μm. (**D**) Heatmap showing Log2 fold change (L2FC) of all DEGs identified in astrocytes in the genotype comparisons indicated (FDR < 0.05). Graphs (right) indicate total number of DEGs (black bars) and number of up-regulated/down-regulated DEGs (red and blue bars) for each genotype comparison. (**E**) Volcano plot of PS19 versus WT (top) or KO_PS19 versus PS19 DEGs (FDR < 0.05). DAA genes are shown in orange, and murine *Sorl1* is indicated in black. (**F**) Heatmap showing Log2 fold change of all DEGs identified in microglia in the genotype comparisons indicated (FDR < 0.05). Graphs (right) indicate total number of DEGs (black bars) and number of up-regulated/down-regulated DEGs (red and blue bars) for each genotype comparison. (**G**) Heatmap depicting Log2 fold change in DAM, homeostatic, and MHC transcripts in the genotype comparisons indicated. Empty and filled plots represent female and male animals as indicated.

We then sampled ROIs in CA1, CA3, and DG in WT, PS19, KO, and KO_PS19 hippocampus in ROIs stained for astrocytes (GFAP) and microglia (IBA1) (Fig. 9, A to C). We observed more astrocyte DEGs in KO_PS19 versus WT (411 DEGs) compared to PS19 versus WT (224 DEGs) (Fig. 9D and table S4), indicating that similar to neurons, SORLA deletion can enact transcriptomic changes in PS19 astrocytes. We find that SORLA deletion can increase expression of astrocyte DAA DEGs over PS19 (KO_PS19 versus PS19; *Serpina3n*, *Ctsb*) as well as KO (KO_PS19 versus KO; *Serpina3n*, *C4b*) (Fig. 9E, fig. S15F, and table S4), indicating that SORLA deletion can aggravate astrocyte pathology in aged PS19 brain. In microglia, we also observed more KO_PS19 versus WT DEGs (43 DEGs) compared to PS19 versus WT (24 DEGs) (Fig. 9F and table S4), and DAM signatures showed significant up-regulation in KO_PS19 hippocampus compared to KO (Fig. 9G, fig. S15G, and table S4). These results indicate that SORLA deletion can exacerbate pathological expression profiles in neurons, astrocytes, and microglia in PS19 hippocampus.

**Fig. 10.**
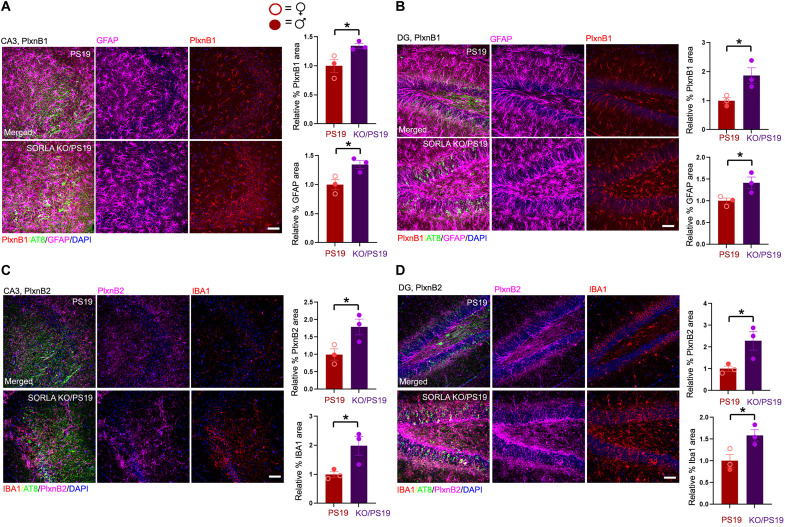
SORLA deletion up-regulates PlxnB1 and PlxnB2 levels in PS19 hippocampus. (**A** to **D**) Representative images and quantification of PlxnB1and PlxnB2 in CA3 [(A) and (C)] and DG [(B) and (D)] from 9 MO PS19 and SORLA KO/PS19 hippocampus as indicated. FFPE slides stained for PlxnB1 (red) were counterstained for AT8 ptau (green), GFAP (purple), and nuclei (DAPI, blue); FFPE slides stained for PlxnB2 (purple) were counterstained for IBA1 (red), AT8 ptau (green), and nuclei (DAPI, blue) (scale bar, 50 μm). Adjacent graphs depict relative PlxnB1, GFAP, PlxnB2, and IBA1 staining area from PS19 and SORLA KO/PS19 (“KO/PS19”) in hippocampal CA3 [(A) and (C)] or DG [(B) and (D)] regions quantified from 9 MO animals; plots represent quantified measurements from individual animals, normalized to PS19 (set to 1.0). Graphs represent mean ± SE; statistical significance was determined by unpaired Student’s *t* test [(A) to (D)], *P < 0.05. Empty and filled plots represent female and male animals as indicated.

As Sema4D/PlxnB1 and B2 interactions in astrocytes, neurons, and microglia are up-regulated in PS19 brain and reduced with SORLA up-regulation, we also characterized potential alterations in Sema4D and PlxnB1/B2 pathology in SORLA KO/PS19 hippocampus. We observed increased Sema4D levels in KO_PS19 (homozygous *Sorl1* deletion/PS19) compared to PS19 hippocampus by immunoblot, with no significant change in het_PS19 (heterozygous *Sorl1* deletion/PS19) compared to PS19 in 9 MO hippocampus (fig. S16A). In addition, we observed general up-regulation of both Sema4D and PlxnB2 in microglia GeoMx transcriptomic profiles in KO_PS19 hippocampus compared to PS19 and control (fig. S16B). PlxnB1/B2 and Sema4D levels have been shown to be up-regulated in aged thy-TAU22 mice by bulk RNA-seq analysis (*78*) (fig. S16C). We also characterized effects of SORLA deletion on PS19-dependent PlxnB1/B2 pathology by immunohistological analysis and observed a significant increase in PlxnB1 and PlxnB2 levels in CA3 and DG regions in SORLA KO/PS19 as well as GFAP and IBA1 staining, respectively, compared to PS19 alone (Fig. 10, A to D). Correlation analysis comparing PlxnB1 or B2 staining intensity in comparison with AT8 ptau levels showed a trending positive, yet nonsignificant correlation for PlxnB and AT8 ptau in PS19 and SORLA KO/PS19 hippocampus (fig. S16D).

Given that our results indicate that SORLA suppresses PlxnB1/B2 levels in tauopathy mouse brain, we determined whether *PLXNB* levels correlate with SORLA expression in human AD brain (*77*). We observed a strong inverse correlation between *PLXNB1* and SORLA expression across both human control and AD brain, indicating that higher SORLA expression correlates with reduced *PLNXB1* levels in human brain (fig. S16E). In summary, our results demonstrate a protective role for SORLA in suppressing pathological effects associated with tauopathy and tau proteotoxicity in PS19 mouse brain and implicate a conserved inverse relationship between SORLA and *SEMA4D/PLXNB* signaling in AD brain.

## DISCUSSION

We demonstrate that SORLA up-regulation in PS19 mice expressing human P301S tau attenuates pathological phenotypes in PS19 brain, including ventricular enlargement, tau phosphorylation and seeding, synaptic loss, impaired plasticity, and glial hyperactivation (fig. S17). Given that SORLA up-regulation has little effect on AT8 ptau levels in early stages of pathology where astrogliosis is not yet seen, it seems likely that SORLA-dependent suppression of tau phosphorylation and aggregation in neurons can, at least in part, suppress glial activation phenotypes, including the induction of pathogenic drivers such as ApoE and C1q previously described in PS19 brain, as well as aggravators of gliosis such as PlxnB1 and B2 not yet described in primary tauopathy. SORLA up-regulation can also enhance tau uptake and trafficking through the endolysosomal pathway in neuron and microglia, which can potentially limit tau cytosolic escape or tau release, pathological aggregation, and spreading. Together, this implicates a model where SORLA can suppress early tau pathology to suppress feed-forward enhancers of glial activation and tau pathogenesis (fig. S17). We discuss the implications of genetic interactions between SORLA and suppression of tau pathology and glial-based drivers as characterized here.

### SORLA-associated attenuation of tau pathogenesis

Recent evidence suggests that SORLA can bind tau directly through interactions with the N-terminal SORLA VPS10 domain, and SORLA can promote tau aggregation and seeding from tau-enriched fractions in AD brain samples in an HEK293 tau-RD FRET biosensor model (*79*), suggesting that SORLA can affect cytosolic seeding of tau internalized from an extracellular source. However, interactome studies characterizing tau complexes in iPSC-derived neurons (*61*) and tau-seed interactomes from PS19 mice (*80*) failed to detect SORLA in tau complexes. Our results here indicate that SORLA up-regulation can attenuate, while SORLA deletion can aggravate pathological tau phosphorylation and seeding in aged PS19 mouse brain, where tau pathology resides mostly within neurons. This suggests that short-term modulation of SORLA in a human cell line may have differing effects on exogenously internalized tau (*79*) compared to global SORLA modulation in aged PS19 mouse brain. Given that tau pathology originates in neurons, effects of SORLA in suppressing tau phosphorylation and aggregation in neurons may be key to attenuating pathogenic effects related to tau. This may trigger other events related to tau pathogenesis and toxicity; for example, SORLA up-regulation can suppress induction of key drivers of tau pathogenesis such as C1q (*52*, *53*) and ApoE (*38*). In support of the notion that SORLA may be in fact protective with respect to tau pathology/pathogenesis, recent work indicates that SORLA deletion in iPSC-derived neurons enhances tau phosphorylation (*31*). Because SORLA expression is thought to be reduced (*25*, *26*) or down-regulated by AD-associated stop-gain variants (*7*, *12*, *13*), it is likely that SORLA dysfunction is deleterious to tau pathogenesis.

Given that tau pathology predominantly resides within neurons, we consider that SORLA up-regulation has a particularly strong effect in limiting tau pathology in neurons. Previous groups have reported that SORLA deletion results in endosomal (*45*, *47*) and lysosomal defects (*47*) in iPSC-derived neurons. Our results indicate that SORLA up-regulation enhances tau uptake in both neurons and microglia, and increases tau trafficking to late endosomes/lysosomes in microglia. Thus, SORLA promotes lysosomal tau trafficking and potentially limits endolysosomal escape in neurons, while enhancing tau trafficking into degradative compartments such as lysosomes in microglia. While it is not possible to specifically determine how cell-specific modulation of SORLA can affect tau using the global transgenic overexpression/deletion models used here, we are interested in further characterizing specific effects of SORLA on tau in neurons, and the extent of SORLA modulation on glia in influencing overall tau pathology.

### SORLA, ApoE, C1q, and the synapse in models of AD and tauopathy

Our results indicate that SORLA up-regulation could affect synaptic integrity and function through mechanisms associated with synaptic stability and/or elimination. In addition to being key drivers of tau pathogenesis in PS19 brain, ApoE and C1q have been shown to be important mediators of synaptic function. Although numerous genetic risk factors have been identified in sporadic AD, commonalities between various risk factors such as *APOE* and various *SORL1* variants remain somewhat unclear. In the case of APOE, dysregulation of these AD risk factors affects both Aβ and tau pathogenesis in various mouse models. *Apoe* deletion in mice results in synaptic loss and cognitive impairment, which could be restored by peripheral *Apoe* expression (*81*). Murine *Apoe* deletion reduced Aβ deposition in various transgenic APP mouse models (*82*–*86*), and expression of human ApoE4 is seen to exacerbate pathological features including tau pathology, ventricle dilation, and glial activation (*42*). Removal of the neuronal APOE4 transgene in APOE4/PS19 mice attenuates tau pathology, ventricle dilation, gliosis, and neuronal hyperexcitability (*39*), indicating that APOE is a mediator of both Aβ and tau pathogenesis. Retromer deficiency or dysregulation, which has been observed in AD brain (*22*), can also perturb ApoE levels; for example, neuronal *Vps35* deletion can elevate ApoE in mouse CSF (*87*) as well as hippocampus, likely through glial activation observed with neuronal *Vps35* deletion (*88*). SORLA up-regulation decreased bulk ApoE protein levels in PS19 mice, which was consistent with microglia *Apoe* mRNA expression profiles in SORLA TG/PS19 hippocampus. Although *Apoe* is expressed at very low levels in neurons, our results show that SORLA overexpression up-regulates *Apoe* expression in PS19 neurons. This finding aligns with previous studies showing that SORLA positively regulates ApoE expression in iPSC-derived neurons (*31*). TGF-β/SMAD signaling represses *APOE* expression, and TGF-β inhibition can elevate *APOE* levels in a SORLA-dependent manner in iPSC-derived neurons (*31*). In contrast, microglial *Apoe* mRNA is elevated in PS19 mice, coinciding with increased DAM signatures. Both DAM markers and *Apoe* mRNA levels are significantly reduced in SORLA TG/PS19 microglia compared to PS19, suggesting that SORLA overexpression mitigates tau pathology, which in turn suppresses DAM activation and consequent *Apoe* induction in microglia. These findings provide previously unidentified mechanistic insight into the dual roles of SORLA as a cell-autonomous regulator of neuronal ApoE, by potentially promoting lipid recycling in neurons, and as a modulator of microglial *Apoe* expression and activation. It would be valuable to investigate how SORLA potentially modulates tau pathology in the context of varying *APOE* variants in future studies.

Previous studies have shown that expression of human APOE4 can enhance C1q accumulation and increase astrocyte-mediated synaptic pruning in mouse brain (*89*); similarly, iPSC-derived APOE4/E4 organoids feature enhanced apoptosis and impaired synaptic integrity compared to APOE3/E3 organoids (*90*). In addition to effects observed with APOE4 in enhancing C1q accumulation in mouse brain (*89*), ApoE can bind to activated C1q and inhibit activation of the classical complement cascade (*91*). ApoE/C1q complexes also correlated with cognitive impairment and atherosclerosis in the choroid plexus in mouse and human AD brain, implicating that disease pathogenesis may involve in direct interactions between ApoE and C1q (*91*). C1q is a component of the classical complement pathway and has been shown to be important for tagging synapses for microglial-mediated uptake and elimination in the retinogeniculate early during development (*56*). In AD, C1q is involved in pathologically labeling synapses for microglial elimination in response to Aβ proteotoxicity in J20 (hAPP) (*57*), as well as PS19 mice (*92*). C1q also contributes to astrocyte-mediated synapse elimination in PS19 mouse brain (*53*), and is an important component of microglia-dependent astrocyte activation in AD (*93*). Given that SORLA can reduce C1q expression and restore LTP in PS19 hippocampus, together with observations that SORLA can suppress microglia activation, SORLA may potentially suppress pathological C1q production or uptake at synapses in glia. On the basis of these results together, SORLA can attenuate a spectrum of pathogenic effects associated with these components ranging from synaptic uptake to neuroinflammatory glial activation.

### Alterations in cell-cell interactions at the synapse and in glia

We observed changes in pathological ligand/receptor pairs in PS19 hippocampus that were attenuated with SORLA up-regulation. We were particularly interested in potential changes in the semaphorin family of extracellular ligands and the plexin family of semaphorin receptors. PlxnB1 correlates with Aβ pathology and cognitive impairment in human AD brain (*73*) and Sema4D is a potential driver of astrogliosis in AD mouse brain (*72*). PlxnB1 is implicated as a driver of astrocyte signatures associated with phenotypic cognitive decline in AD, and *PLXNB1* deletion in human iPSC-derived or murine astrocytes can reverse pathogenic signatures associated with cognitive impairment (*94*). PlxnB1 levels are also associated with increased Aβ load and clinical degenerative features by targeted proteomic analysis in human AD brain (*95*) and can regulate microglia/astrocyte distancing and activation, where *Plxnb1* deletion can enhance plaque compaction and reduce neuroinflammation in AD (APP/PS1) mouse brain (*96*). Sema4D/PlxnB2 interactions were also implicated in glial activation in response to experimental autoimmune encephalomyelitis (EAE) demyelination models of MS (*97*). Our observations that PlxnB1 and PlxnB2 are both highly up-regulated in astrocytes and microglia in PS19, respectively, indicate that semaphorin/plexin signaling is potentially a tau-dependent driver of glial activation normalized with SORLA up-regulation. As our results strongly indicate that SORLA up-regulation can attenuate both microglia and astrocyte activation, it may be interesting to determine in future studies whether attenuation of ApoE/C1q and semaphorin/plexin individually or coordinately contribute to pathogenic phenotypes in PS19 mouse brain, and explore relationships between SORLA expression and ApoE/C1q as well as semaphorin/plexin in human AD and tauopathy brain.

### Limitations

Here, we have established pleiotropic effects in reversing features associated with tau toxicity in PS19 mouse brain with global SORLA up-regulation and deletion. Although we describe potential expression-based mechanistic ties related to SORLA and expression of C1q, APOE, and other factors related to gliosis (Sema4d and PlxnB), a molecular mechanism related to SORLA trafficking and/or tau binding remains unclear (fig. S17). One possibility is that SORLA can potentially affect endolysosomal trafficking and turnover of other factors known to modulate tau aggregation and seeding in neurons (*61*). Another possibility is that SORLA may affect both neuronal tau aggregation and glial response to tau concurrently; use of cell-specific SORLA overexpression or deletion models will further clarify potential cell-specific effects for SORLA in suppressing tau pathology and toxicity.

Although we are unable to clearly distinguish functional cell-specific effects associated with transgenic SORLA expression and deletion using the models described in this study, SORLA is expressed in a variety of CNS cell types, and pathological effects due to AD stop-gain variants are likely derived from combined effects from glia, neurons, and functional cross-talk between various CNS cell types. Although the P301S tauopathy model used here features varied, yet strong pathological effects and is widely characterized in the AD/tauopathy field, humanized tau models such as the hTau model comprising a P1-derived artificial chromosome copy of the human *MAPT* gene locus in a murine *Mapt* KO background (*98*–*100*) also feature age-dependent tau pathology, neuronal loss, and memory deficits (*100*–*102*). The hTau-KI human *MAPT* KI mouse model lacks apparent tau pathology, but has been shown to enhance tau propagation with exposure to AD-derived tau aggregates (*103*, *104*). Given that SORLA is an AD risk variant, and AD onset largely occurs independently of *MAPT* tau variants, whether our observations here can be also reflected in other humanized tauopathy mouse models would be interesting. We also note that use of the PS19 tauopathy model only features tau pathology in the absence of Aβ pathology; transgenic or humanized APP models may be needed to better recapitulate neurodegenerative effects associated with SORLA modulation in AD. We anticipate that future studies using humanized *MAPT*, and/or Aβ/tau models in combination, as well as cell-specific SORLA KO models will allow us to further distinguish and characterize cell-specific effects associated with SORLA dysregulation in AD and related tauopathies.

## MATERIALS AND METHODS

### Mouse lines

SORLA-Rosa26 (SORLA TG or *Rosa26 ^CAG-Sorl1/+^*) mouse lines, previously established on a Balb/c background, overexpress SORLA through a CMV/β-actin promoter element (*105*). SORLA KO (*Sorl1^−/−^*) mice were obtained from T. Willnow (Max Delbruck Center for Molecular Medicine, Berlin) with hybrid (129SvEmcTer C57BL6N) or genetic (129SvEmcTer Balbc) backgrounds (*26*). PS19 mouse lines [B6;C3-Tg(Prnp-MAPT*P301S)PS19Vle/J; 1N4R tau] (the Jackson Laboratory, catalog no. 008169) were crossed into SORLA TG lines and SORLA KO lines; littermates from SORLA TG × PS19 and SORLA KO × PS19 were characterized at the indicated time points. Mouse lines were housed with littermates with free access to food and water with a 12-hour light/day cycle. All procedures involving animals were performed under the guidelines of the Sanford-Burnham Medical Research Institute Institutional Animal Care and Use Committee; the related approved animal use forms are 25-041, 23-051, and 23-042.

### Immunoblot

For immunoblot analysis, 9 MO WT, SORLA TG, PS19, and SORLA TG/PS19 mice or 7 MO WT, SORLA KO, PS19, and SORLA KO/PS19 mice were anesthetized and subjected to intracardial perfusion with 1× phosphate-buffered saline (PBS). Hippocampal tissues from each hemisphere were isolated, snap frozen on dry ice, and stored at −80°C. Lysates from hippocampal mouse tissue were extracted by homogenizer beads and resuspended in radioimmunoprecipitation assay (RIPA) buffer in the presence of protease and phosphatase inhibitor cocktail. Protein concentration was measured using a Pierce BCA Protein Assay Kit. Proteins were separated by 4 to 20% Bio-Rad Criterion TGX Precast Gels, transferred onto PVDF membranes, and blocked in 5% nonfat milk in 1× PBS. Blots were then probed with primary antibodies (mouse anti-SORLA, 1:1000, BD no. 611861; mouse anti-AT8, 1:1000, Invitrogen no. MN1020B; mouse anti-T13, 1:2000, BioLegend no. 835201; mouse anti-HT7, 1:2000, Invitrogen no. MN1000; mouse anti-Vinculin, 1:5000, Millipore Sigma no. V9264; Rabbit anti-APOE, 1:1000, Abcam no. ab183597; Rabbit anti–β-actin, 1:5000, Cell Signaling Technology no. 4967; rabbit anti-Iba1, 1:500, Wako no. 016-20001; mouse anti-GFAP, 1:1000, Cell Signaling Technology no. 3670; goat anti-VPS35, 1:1000, Abcam no. ab10099; rabbit anti-VPS26a, 1:1000, Cell Signaling Technology no. 75357; rabbit anti-VPS26b, 1:1000, Bio-Techne no. NBP1-92575; and rabbit anti-VPS29, 1:500, Cell Signaling Technology no. 73540) in 5% bovine serum albumin (BSA)/1× PBS overnight at 4°C, washed in 1× PBS with 0.1% Tween-20, and probed with horseradish peroxidase–conjugated secondary antibodies (Cell Signaling Technology, 1:5000) for 2 hours at room temperature (RT). After washing in 1× PBS, blots were then incubated with ECL Chemiluminescent Substrate and immunoblot signals were acquired using a Chemidoc imaging system. For blot analysis, bands were quantified by Fiji ImageJ (NIH), and targeted proteins were normalized to actin or vinculin and plotted relative to WT (set to 1).

### Immunohistochemistry

The following were anesthetized and subjected to intracardial perfusion with 1× PBS: 9 MO WT, SORLA TG, PS19, and SORLA TG/PS19 mice or 9 MO WT, SORLA KO, PS19, and SORLA KO/PS19 mice. Half hemi-brains were postfixed with 4% PFA, dehydrated in 30% sucrose, embedded in O.C.T., and subjected to cryostat sectioning. Free-floating tissue sections (30 μm) were rinsed in 1× PBS, incubated with blocking solution (0.5% Triton X-100 and 5% BSA in PBS) for 2 hours at RT, immunostained with primary antibodies (mouse anti-AT8, 1:300, Invitrogen no. MN1020B; goat anti-Iba1, 1:200, Abcam no. ab5076; rabbit anti-GFAP, 1:300, Invitrogen no. PA5-16291; guinea pig anti-NeuN, 1:500, Millipore Sigma no. ABN90; rat anti-CD68, 1:200, Bio-Rad no. MCA1957; rabbit anti-C1q, 1:300, Abcam no. ab182451; rat anti-Clec7a, 1:200, Bio-Rad no. MCA2289T; rabbit anti-P2y12, 1:200, Cell Signaling Technology no. 69766; guinea pig anti-PSD95, 1:300, Synaptic Systems no. 124014; rabbit anti-Vglut1, 1:400, Abcam no. ab227805; rabbit anti-Plexin B1, 1:200, Bioss no. BS-2693R; sheep anti-Plexin B2, 1:100, Bio-Techne no. AF6836, goat anti-GFAP, 1:300, Abcam no. ab53554; and rabbit anti-Iba1, 1:200, Wako no. 019-19741) overnight at 4°C, then washed in PBS (3×) and subsequently incubated with Alexa Fluor Plus Secondary Antibodies (Invitrogen, 1:500) for 2 hours at RT. After washing in 1× PBS, brain sections were mounted with DAPI (4′,6-diamidino-2-phenylindole) Fluoromount-G and overlaid with glass coverslips. Images were acquired using a Zeiss confocal microscope (LSM 710) or APERIO ScanScope FL (Leica) system at 10×, 20×, or 60× magnification. For each comparison, all groups within a replicate experiment were stained simultaneously to avoid batch variation and imaged at the same fluorescent intensity. To quantify percent area covered, an optimal threshold was established for each stain in Fiji ImageJ, and all the samples were quantified using matched threshold settings.

### Volumetric analysis

Every 10th coronal brain section (30 μm) starting from the appearance of hippocampus to the dorsal end of the hippocampus was mounted for each mouse. The brain slides were stained with 0.1% Sudan black (Sigma-Aldrich) in 70% ethanol at RT for 20 min, washed in 70% ethanol for 1 min (3×), washed in distilled water (3×) and mounted with Fluoromount-G, and overlaid with a glass coverslip. Slides were scanned using an Aperio AT2 scanner (Leica) at 10× magnification, and the ventricle and hippocampus area were measured using Aperio ImageScope Software (Leica).

### Three-dimensional reconstructions

For microglia engulfment and synapse analysis, brain sections were imaged on a Zeiss LSM 880 microscope at 63× magnification with 0.39-μm *z* sections. Image stacks with Iba1, PSD95, and Vglut1 or C1q-stained sections were used in three-dimensional (3D) reconstructions rendered using Imaris software 9.9. Vglut1 and PSD95 puncta within a size range of ~0.5 to 1.1 μm were detected using the Imaris spot function to create discrete spheres with coordinates indicating center of mass; putative synapses were detected by quantification of pre- and postsynaptic spheres opposed to each other with a maximum distance of 1 μm (sum of the maximum puncta radius) according to published protocols (*106*–*108*). Iba1-positive microglia were 3D reconstructed using the surface rendering function and Vglut1, PSD-95, and C1q puncta inside the microglia were quantified. Six to nine microglia within the hippocampal CA1 region were analyzed per mouse.

### Sample preparation for proteomics analysis and LC-MS/MS

Proteomics analysis and liquid chromatography–tandem mass spectrometry (LC-MS/MS) was performed in the Sanford Burnham Prebys Bioinformatics Core. Frozen 9 MO hippocampus tissue from WT, SORLA TG, PS19, and SORLA TG/PS19 mouse brain were homogenized and resuspended in RIPA buffer in the presence of protease and phosphatase inhibitor cocktail. Protein concentration was quantified using a Pierce BCA Protein Assay Kit. Proteins were then reduced by the addition of 5 mM tris(2-carboxyethyl) phosphine at 30°C for 60 min, followed by alkylation of cysteines with 15 mM iodoacetamide for 30 min in the dark at RT. Urea concentration was reduced to 1 M by adding 50 mM ammonium bicarbonate. Samples were digested overnight with Lys-C/trypsin at RT with constant agitation at a 1:25 enzyme:protein ratio. Following digestion, samples were acidified using 0.1% FA and desalted using AssayMap C18 cartridges mounted on an Agilent AssayMap BRAVO liquid handling system. Cartridges were sequentially conditioned with 100% acetonitrile (ACN) and 0.1% FA; samples were then loaded, washed with 0.1% FA, and eluted with 60% ACN and 0.1% FA. Peptide concentration was determined using a NanoDrop spectrophotometer. Samples were subjected to mass spectrometry analysis using an EASY nanoLC system. Buffer A consisted of H_2_O/0.1% FA; buffer B consisted of 80% ACN/0.1% FA. Samples were separated over a 90-min gradient of increasing buffer B on an analytical C18 Aurora column (75 μm by 250 mm, 1.6 μm particles; IonOpticks) at a flow rate of 300 nl/min. The mass spectrometer was operated in positive data-dependent acquisition mode, and the Thermo FAIMS Pro device was set to standard resolution with the temperature of FAIMS inner and outer electrodes set to 100°C. A three-experiment method was set up where each experiment used a different FAIMS Pro compensation voltage: −50, −70, and −80 V, and each of the three experiments had a 1-sd cycle time. A high-resolution MS1 scan in the Orbitrap [mass/charge ratio (*m*/z) range 350 to 1500, 60 k resolution at *m*/*z* 200, AGC 4 × 10^5^ with a maximum injection time of 50 ms, RF lens 30%] was collected in top speed mode with 1-sd cycles for survey and MS/MS scans. For MS2 spectra, ions with charge states between +2 and +7 were isolated with quadruple mass filter using a 0.7 *m*/*z* isolation window, fragmented with higher-energy collisional dissociation with a normalized collision energy of 30%, and the resulting fragments were detected in the ion trap as rapid scan mode with an AGC of 5 × 10^4^ and a maximum injection time of 35 ms. The dynamic exclusion was set to 20 s with a 10 parts per million mass tolerance around the precursor.

### Proteomic data analysis

Raw files were searched with SpectroMine software using default settings. The search criteria were set as follows: Full tryptic specificity was identified where two missed cleavages were allowed; carbamidomethylation (C) was set as a fixed modification and oxidation (M) was set as a variable modification. The false identification rate was set to 1%. Spectra were searched against the curated UniProt *Mus musculus* database including human Tau protein sequence and common contaminants from the GPM cRAP sequences. Data were further processed using the MSstats package (version 4.2) in R (version 4.1.2). We avoided imputation of missing values before statistical testing using MSstats; instead, we calculated pseudo Log2 fold change (L2FC), adj *P* value, and *P* value of proteins completely missing in one condition in proteins with missing values. The imputed (pseudo) L2FC was calculated as the sum of intensities of the protein (i.e., sum of feature intensities of a given protein within a given sample) across all replicates of the same group that it was detected, divided by 3.3. For these proteins, the imputed *P* value and adj *P* value was calculated by dividing 0.05 or 0.1, respectively, by the number of replicates that the given protein was confidently identified, and multiplied by the number of peptide features used for quantification in the given protein. Thus, the imputed L2FC provides an estimate of the protein abundance in the experimental conditions detected, while the imputed *P* value or adj *P* value reports the confidence of the imputation and reflects the consistency of protein detection across replicates in the experimental group or conditions it was detected. Custom searches were performed for human SORLA (expressed in SORLA TG animals) and MAPT (human P301S tau).

PCA was carried out in R version 4.1.2 with the PCATools package (version 2.6.0) using Log2 protein intensity for all significantly selected proteins summarized by the dataProcess function from MSstats (version 4.2); multiple testing correction was performed with the Benjamini and Hochberg approach using MSstats. To calculate *z*-score values within each replicate, row (protein-wise) *z*-scores were computed in R version 4.0.2 using the scale function by subtracting the mean intensity of each protein from the corresponding intensities of the biological replicates, and dividing the resulting values by the SD of the intensities. GO analysis of grouped DEP gene lists was performed using GO DAVID (https://davidbioinformatics.nih.gov/).

### Electrophysiology

For analysis of long-term potentiation (LTP), ex vivo hippocampal slices were prepared from 6 MO WT, SORLA TG, PS19, and SORLA TG/PS19 mice as described previously (*109*, *110*). Under terminal isoflurane anesthesia, mice were transcardially perfused with ice-cold, sucrose-based artificial cerebrospinal fluid (aCSF) of the following composition: 190 mM sucrose, 25 mM d-glucose; 25 mM NaHCO_3_, 3 mM KCl, 1.25 mM NaH_2_PO_4_, 5 mM MgSO_4_, 10 NaCl mM, and 0.5 mM CaCl_2_ saturated with carbogen (95% O_2_/5% CO_2_) and adjusted to pH 7.4 and 300 mOsmol. After perfusion, mice were decapitated, and brains were dissected and cut into a block of live brain tissue containing the hippocampus and cortex. A vibrating-blade microtome (Leica VT1200S) was used to cut 400-μm-thick horizontal slices containing both cortex and hippocampus. Slices were transferred to a holding chamber containing warm (34°C) aCSF of the following composition: 125 mM NaCl, 25 mM NaHCO_3_, 3.0 mM KCl, 1.25 mM NaH_2_PO_4_, 2.0 mM CaCl_2_, 1.0 mM MgSO_4_, and 10 mM d-glucose saturated with carbogen (95% O_2_/5% CO_2_) adjusted to pH 7.4 and 300 mOsmol. Slices were left to recover at RT in oxygenated aCSF for 2 hours, transferred to a recording chamber, and perfused with warm (32°C) oxygenated aCSF containing 100 μM picrotoxin (*111*) at a rate of 2 ml/min. For recording, both stimulating and recording electrodes were positioned within the dentate gyrus respectively on an upright microscope (Olympus). An electrical stimulation protocol was used to evoke field excitatory postsynaptic potentials (fEPSP) in the middle of the molecular layer of the dentate gyrus. LTP was induced by two trains of high-frequency stimulation (100 Hz for 1 s at 20-s intervals) using a concentric bipolar stimulating electrode. fEPSPs were recorded using glass electrodes filled with aCSF and placed in the molecular layer of the dentate gyrus (dendritic region). All recordings of synaptic activity were carried out using a Multiclamp 700B, and signals were filtered at 3 kHz, digitized, and sampled using pClamp10 software. The magnitude of potentiation was calculated as percentage (%) change in fEPSP slope normalized to baseline values. The average change in synaptic potentiation at 1 hour following induction of LTP was used to compare different experimental groups.

### Nucleus isolation and snRNA-seq library preparation

Three frozen 9 MO hippocampus tissue from WT, PS19, and SORLA TG/PS19 groups were pooled and lysed in Miltenyi nuclei extraction buffer containing RNase inhibitor (0.2 U/μl) using a gentleMACS Octo Dissociator (Program 4C_nuclei_1) according to the manufacturer’s protocol. Miltenyi Debris removal solution was applied to the nuclei suspension, and NeuN-AF488 and Olig2-AF647 were added to the samples and incubated on ice for 30 min. Sort buffer [RNase inhibitor (1 U/μl) and 1% BSA in PBS] was added to 4 ml, and DAPI was added at a 1:1000 dilution; the mixture was centrifuged at 1500*g* for 5 min and pelleted nuclei were resuspended in 500 μl of sort buffer. Nuclei were then vortexed before subjecting samples to FACS sorting. To obtain a sufficient number of microglial nuclei for analysis, 150,000 nuclei (50% NeuN-/Oligo2- and 50% non-NeuN-/Olig2-) were sorted into 90 μl of collection buffer [RNase inhibitor (5 U/μl) and 10% BSA in PBS]. A total of 10,000 nuclei were used to generate Gel Beads-in-emulsions and barcoded using the 10X Genomics Chromium Next GEM Single Cell 3′ Reagent Kit v3.1 (Dual Index) following the manufacturer’s instructions. Libraries were sequenced to a median depth of approximately 50,000 reads per nucleus using an Illumina NovaSeq 6000 instrument at the UC San Diego IGM Genomics Center.

### snRNA-seq data processing, doublet detection, and removal

Cell Ranger count was used to align samples to the reference genome (mm10), quantify reads, and filter reads with a quality score below 30. Aggregated and sequencing depth normalized count matrix from Cellranger aggregate (*cellranger aggr*) output was used for downstream analysis using Seurat version 4.0.5 (*112*) and R version 4.0.2. Lower-quality cells with >10% mitochondrial reads and top 2% quantile of nFeature_RNA (number of detected genes) were removed. We used a two-step strategy to identify and remove potential doublets and multiplets. The Scrublet29 method over a random subset of 30,000 barcoded nuclei was used to identify clusters of doublets in the resulting snRNA-seq library. Identification of heterotypic doublets comprising two different cell types was performed using Scrublet by simulating doublets, and through subsequent generation of nearest-neighbor classifiers. During the first step, we applied the scrublet method version 0.2.3 (*113*) to identify potential doublets for each sample, using the raw count matrix with default settings. During the second step, we identified doublets/multiplets using scds package version 1.12.0 (*114*) in R version 4.2.1 as follows. The output of Cellranger aggregate was split into individual samples. Each sample was processed independently using Seurat pipeline to cluster cells. Briefly, raw data were normalized using *NormalizeData*. The top 2000 highly variable genes were identified using *FindVariableFeatures*. Data were scaled using *ScaleData*. Sequencing depth (nCount_RNA) and percent mitochondrial reads were regressed out during the *ScaleData* step. PCA on the scaled data was run using *RunPCA*. Cells were clustered using *RunUMAP*, *FindNeighbors*, and *FindClusters* with the top 25 PCA dimensions and *resolution* = 0.5. Seurat objects were converted to SingleCellExperiment objects using *as.SingleCellExperiment. cxds*, *bcds*, and *cxds_bcds_hybrid* functions were applied to the datasets to determine doublet scores. Cells with scds doublet score > 0.75 were labeled as doublets and removed from downstream analysis. Data integration was performed using Seurat and Harmony version 0.1.0 (*115*). Aggregated data with the doublets and lower-quality cells removed were first normalized using *NormalizeData*. The top 2000 highly variable genes were identified using *FindVariableFeatures*. Any mitochondrial or ribosomal genes and high expression genes (Malat1 and Xist) were removed from the list of highly variable genes. Data were scaled using *ScaleData*, and the effects of sequencing depth (nCount_RNA) and mitochondrial percentage were regressed out. *RunPCA* was used to perform PCA on the scaled data and subsequently the samples were integrated using Harmony’s *RunHarmony* function with samples as the variable to remove. Cell clustering was performed using *RunUMAP*, *FindNeighbors*, and *FindClusters* with the top 30 PCA dimensions and *resolution* = 0.2. Cluster markers were determined using *FindAllMarkers* in WT sample only. Differential expression was performed using *FindMarkers* and MAST test. *P* value correction for multiple testing using the Bonferroni method was performed using Seurat under default settings. GO analysis of grouped DEG gene lists was performed using GO DAVID (https://davidbioinformatics.nih.gov/). To identify GO CC (Cellular Component) DEGs with little or no expression in microglia, transcripts (*32**,* *33*) that were expressed in less than 1% with an expression *z*-score < −1.33 in microglia were selected as transcripts of low abundance.

### Soluble/insoluble tau extraction

Sarkosyl extraction was performed as previously described (*116*). Tissue was homogenized using a Dounce homogenizer in 10× (v/w) cold resuspension buffer (1× PBS containing 5 mM EDTA, protease, and phosphatase inhibitor cocktail). Tissue debris was then removed by centrifugation at 2000*g* for 5 min at 4°C. For immunoblot analysis, a portion of the homogenate was diluted 1:1 with RIPA buffer (1× PBS containing 5 mM EDTA, protease, and phosphatase inhibitor cocktails; 1% deoxycholate; 1% Triton X-100; and 0.5% SDS), and supernatant was collected after centrifugation at 15,500*g* for 20 min at 4°C. For detergent-insoluble proteins, the remaining homogenate was mixed 1:1 with 2× RIPA buffer lacking SDS and centrifuged at 100,000*g* for 30 min at 4°C. Supernatant (S1) was collected, and the pellet was resuspended in 1× RIPA buffer containing 1% Sarkosyl (without SDS) and incubated for 1 hour at RT on an orbital shaker. The Sarkosyl-soluble fraction (S2) was obtained by centrifugation at 200,000*g* for 30 min at 4°C. The final pellet was resuspended in 1× PBS by sonication (probe sonicator, 30% amplitude, 10 cycles of 2 min on/1 min off; P2). All fractions were stored at −80°C until use for Western blotting or enzyme-linked immunosorbent assay (ELISA). Phospho-tau (pT181) and total tau were determined by the Phospho-Tau ELISA Kit (catalog no. KHO0631, Thermo Fisher Scientific) and the Human Tau (Total) ELISA Kit (catalog no. KHB0041, Thermo Fisher Scientific) according to the manufacturer’s instruction.

### Tau biosensor assay

Tau seeding was performed using a FRET biosensor HEK293T cell line [American Type Culture Collection (ATCC) no. CRL-3275] stably expressing tau-RD P301S-CFP and P301S-YFP (tau-RD cells) as described previously (*117*). A total of 10^5^ cells were plated on poly-d-lysine (PDL)–coated coverslips in a 24-well plate and cultured in Dulbecco’s modified Eagle’s Medium (DMEM) with 10% fetal bovine serum (FBS) and 1% penicillin/streptomycin (Pen/Strep). The following day, the medium was changed, and cells were seeded with brain lysate. Brain lysate was prepared by homogenizing tissue in 10 times the volume of PBS with 0.02% NaN_3_, 1% protease, and phosphatase inhibitor cocktail. After homogenization, lysates were centrifuged at 21,000*g* for 15 min at 4°C. The supernatant was collected, and protein concentration was measured using the BCA assay. Cells were seeded with 7 μg of protein using Lipofectamine 3000 according to the manufacturer’s instructions. After 72 hours of incubation, cells were washed and fixed in 4% PFA, washed and stained with DAPI, and imaged by confocal microscopy (Zeiss 880). Twelve images per condition from biological triplicate were taken for quantification using Image J.

### Size exclusion chromatography

Fresh whole mouse brain was homogenized using a mechanical homogenizer in 300 μl of lysis buffer (20 mM tris-HCl, pH7.5, 5 mM EDTA, 150 mM NaCl, and 1% DDM) containing a protease and phosphatase inhibitor cocktail and centrifuged at 28,000*g* for 30 min at 4°C. Supernatants comprising 1 ml of enriched aggregates were injected onto a Superose 6 Increase 5/150 GL column (Cytiva) with a running buffer composed of 110 mM NaCl, 20 mM Hepes, and 0.1% DDM and ran at 4°C. Absorbance was measured at 280 nm. Following sample application to the column, samples were eluted with 1.5× column volume (CV) running buffer. Fractions were collected after 0.2 CV at a fixed fractionation volume of 150 μl, resulting in a total of 25 fractions collected. Thirty microliters from each fraction was separated on 4 to 20% SDS–polyacrylamide gel electrophoresis gradient gels for immunoblot analysis.

### Primary neuron culture

Pups at postnatal day 0 (P0) were euthanized for neuronal culture; forebrain was dissected in pre-cold Hibernate-A media (1% Pen/Strep) and was digested in 2.5% trypsin at 37°C for 15 min, followed by the addition of FBS and DNase. Digested tissue was filtered using 70-μm strainers and centrifuged at 300*g* for 5 min. Cell pellets were resuspended in DMEM with 10% FBS and 1% Pen/Strep, and seeded on PDL-coated plates for 3 hours, and subsequently changed to neuronal culture medium (neurobasal media with B-27 supplement, 1× GlutaMAX supplement, and 1% Pen/Strep). Half of the media volume was changed every 3 days, and neurons at DIV14 (days in vitro) were used for experiments. For tau uptake assay, recombinant 2N4R human tau 1-441 oligomerized in 30 μM heparin at 37°C for 24 hours. Tau oligomers were subsequently conjugated to Alex555 (“tau-555”) using an Alexa Fluor 555 Microscale Protein Labeling kit according to the manufacturer’s instructions. Tau oligomers were added to cells for 2 hours. For immunostaining, cells were fixed with 4% PFA for 20 min at RT after two washes with PBS. Cells were then permeabilized with 0.5% Triton X-100 in PBS for 5 min, blocked with 5% BSA in PBS for 1 hour, and incubated with primary antibodies (chicken anti-MAP2, 1:1000; mouse anti-T13, 1:500; rabbit anti-EEA1, 1:300; rabbit anti-RAB7, 1:300; and rabbit anti-LAMP1, 1:300) overnight at 4°C. Cells were washed and incubated with Alexa Fluor–conjugated secondary antibodies for 1 hour. Cells were washed and stained with DAPI and imaged by confocal microscopy (Zeiss 880). Images were analyzed by Zeiss arivis software.

### Primary microglia culture

Primary microglia culture was performed as previously described (*110*). Pups at P3 were used for microglia culture. Forebrain was dissected in pre-cold DMEM with 1% Pen/Strep and was treated with Papain at 37°C for 30 min, followed by adding FBS and DNase. Digested tissue was filtered with 70-μm strainers and centrifuged at 300*g* for 5 min. The cell pellets were resuspended in DMEM with 10% FBS and 1% Pen/Strep, and cells were seeded in PDL-coated flasks. Granulocyte-macrophage colony-stimulating factor (25 ng/ml) was added into the cultures after 5 days and removed before harvesting. Microglial cells were harvested twice by shaking (200 rpm, 60 min) 10 to 14 days after plating and subjected to various treatments within 24 hours of harvest.

### Cell-cell interaction analysis

Cell-to-cell interactions (CCIs) were inferred by Cellphone DB (*65*) with the human CCI database (v5.0.0) mapped to mouse orthologs using biomaRt (*118*). Specifically, the “statistical_analysis_method” function was called using default parameters to infer CCIs with mean expression of interacting partners (ligand or receptor) being significantly enriched (*P* value <0.05), compared to random permutation. CCI inference was performed for each sample between pairwise cell-type interactions comprising astrocytes, microglia, and neurons. Significant CCIs between cell pairs with a *P* value <0.05 were used in visualization and selected as candidates for further validation.

### GeoMx digital spatial profiling and analysis

Coronal sections from three 9 MO SORLA KO, PS19, and SORLA KO/PS19 animals were mounted on slides. Tissue sections (5 μm) were prepared by sequential deparaffinization, antigen retrieval (pH 9.0), proteinase K (0.1 μg/ml) digestion (15 min at 37°C), and postfixation, followed by hybridization of tissue with ultraviolet photocleavable probes (Mouse Whole Transcriptome Atlas, NanoString Technologies, Inc) (overnight at 37°C) according to the manufacturer’s standard instructions. The tissue was then labeled with an anti-GFAP antibody conjugated to Alexa-532 (1:50 dilution), an Alexa-594–conjugated anti-Iba1 antibody (1: 50 dilution), and Syto13 (1:10 dilution). A separate slide was labeled with an anti-NeuN antibody conjugated to Alexa-647 (1:50 dilution) and Syto13 (1:10 dilution), and labeled slides were scanned on a GeoMx DSP instrument. Adjacent sections were stained to detect AT8 ptau. ROI selection was performed as follows: for GFAP or Iba1 ROIs, geometric selections were drawn in the CA1 or CA3 region comprising the stratum pyramidale, stratum radiatum, and stratum lacunosum, and within the granule cell layer (GCL)/hilus within the DG region. For NeuN ROIs, tight geometric ROI selections were drawn within the CA1, CA3, or DG GCL. Using the GeoMx segmentation tool, each ROI was then illuminated with an ultraviolet (UV) light to photocleave oligonucleotide probe barcodes from either GFAP+, Iba1+, or NeuN+ cells, which were then collected individually into single wells in a 96-well plate. Library preparation and sequencing were conducted at the SBP Genomics Core on an Element Biosciences AVITI sequencer. GeoMx raw sequencing data were processed using command-line GeoMx NGS Pipeline version 2.3.3.10. Processed data QC, filtering, and normalization were performed using NanoStringNCTools version 1.4.0, GeoMxTools version 3.0.1, and GeoMxWorkflows version 1.2.0 in R version 4.2.1(according to the pipeline provided at https://bioconductor.org/packages/devel/workflows/vignettes/GeoMxWorkflows/inst/doc/GeomxTools_RNA-NGS_Analysis.html). Segment QC parameters were set as “minSegmentReads = 1000, percentTrimmed = 80, percentStitched = 80, percentAligned = 80, percentSaturation = 50, minNegativeCount = 1, maxNTCCount = 1000, minNuclei = 10, minArea = 1000.” Segments that failed the QC metrics were removed from downstream analysis. Segment gene detection limit was set at 5%, and data were normalized using the Q3 method. Normalized expression data for astrocytes (GFAP), neurons (NeuN), and microglia (Iba1) were batch corrected separately using removeBatchEffect from Limma version 3.52.4 (*119*) using brain regions collected as batches. Differential expression analyses were performed using limFit and eBayes functions from Limma package.

### Statistical analysis

All statistical analysis in this study was performed using GraphPad Prism software and RStudio. Differences were assessed by paired or unpaired *t* tests, or one-way or two-way analysis of variance (ANOVA) where appropriate; a minimum of *P* < 0.05 was statistically significant. Results from two-way ANOVA, as well as details pertaining to animal sex in the main figures and supplementary figures, are included in table S5. All experiments in figures (including supplementary figures) were repeated at least three times (independent experiments) unless specified otherwise in the figure legends.
