# Supplementary material for: SORLA up-regulation suppresses pathological effects in aged tauopathy mouse brain
Source: Sci Adv. 2026 Jul 17;12(29):eaed6825. doi: 10.1126/sciadv.aed6825 (PMC13378584; doi:10.1126/sciadv.aed6825)
Supplement: Supplementary file 1 — Figs. S1 to S17 Legends for tables S1 to S5 [file sciadv.aed6825_sm.pdf]

Science Advances
AAAS

Supplementary Materials for

SORLA up-regulation suppresses pathological effects in aged tauopathy mouse brain

Huijie Huang *et al.*

Corresponding author: Timothy Y. Huang, thuang@sbpdiscovery.org

Sci. Adv. **12**, eaed6825 (2026)
DOI: 10.1126/sciadv.aed6825

The PDF file includes:

Figs. S1 to S17
Legends for tables S1 to S5

Other Supplementary Material for this manuscript includes the following:

Tables S1 to S5

Supplementary figures and figure legends

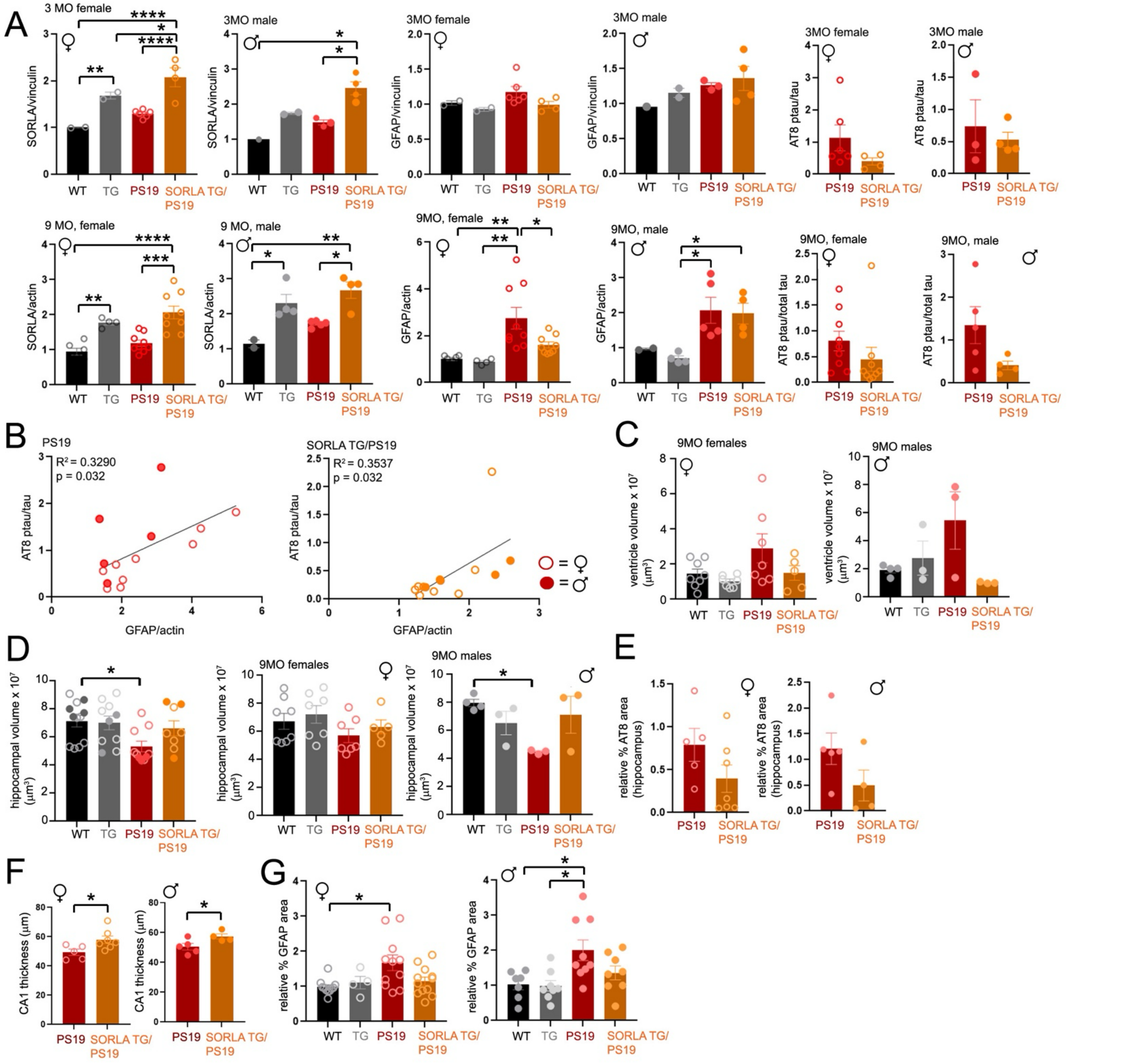

figure S1. Characterizing effects of SORLA upregulation on PS19 mouse hippocampus. (A) Quantification of band intensities in male and female 3- and 9-month-old wildtype (WT), SORLA TG, PS19, and SORLA TG/PS19 mice (as shown in Fig. 1C). (B) Correlation between AT8 ptau and GFAP levels in 9 MO PS19 and SORLA TG/PS19 hippocampus from relative band intensity values quantified in Fig. 1C. (C, D) Ventricle and hippocampus volume quantified in 9MO female and male mice for the genotypes are indicated. (E, F) Quantification of relative AT8 ptau area and CA1 NeuN+ granule cell layer thickness in 9MO male and female mice. (G) Quantification of relative % GFAP area in male and female 9 MO mice. All graphs represent mean±SE. Statistical analysis was determined by Two-way ANOVA then Tukey's multiple comparisons test in (A, C, D, G). Unpaired Student's t-test was conducted in (A, E, F) or simple linear regression (B), *p<0.05, **p<0.01, ***p<0.001, ****p<0.0001. Empty and filled plots represent female and male animals as indicated.

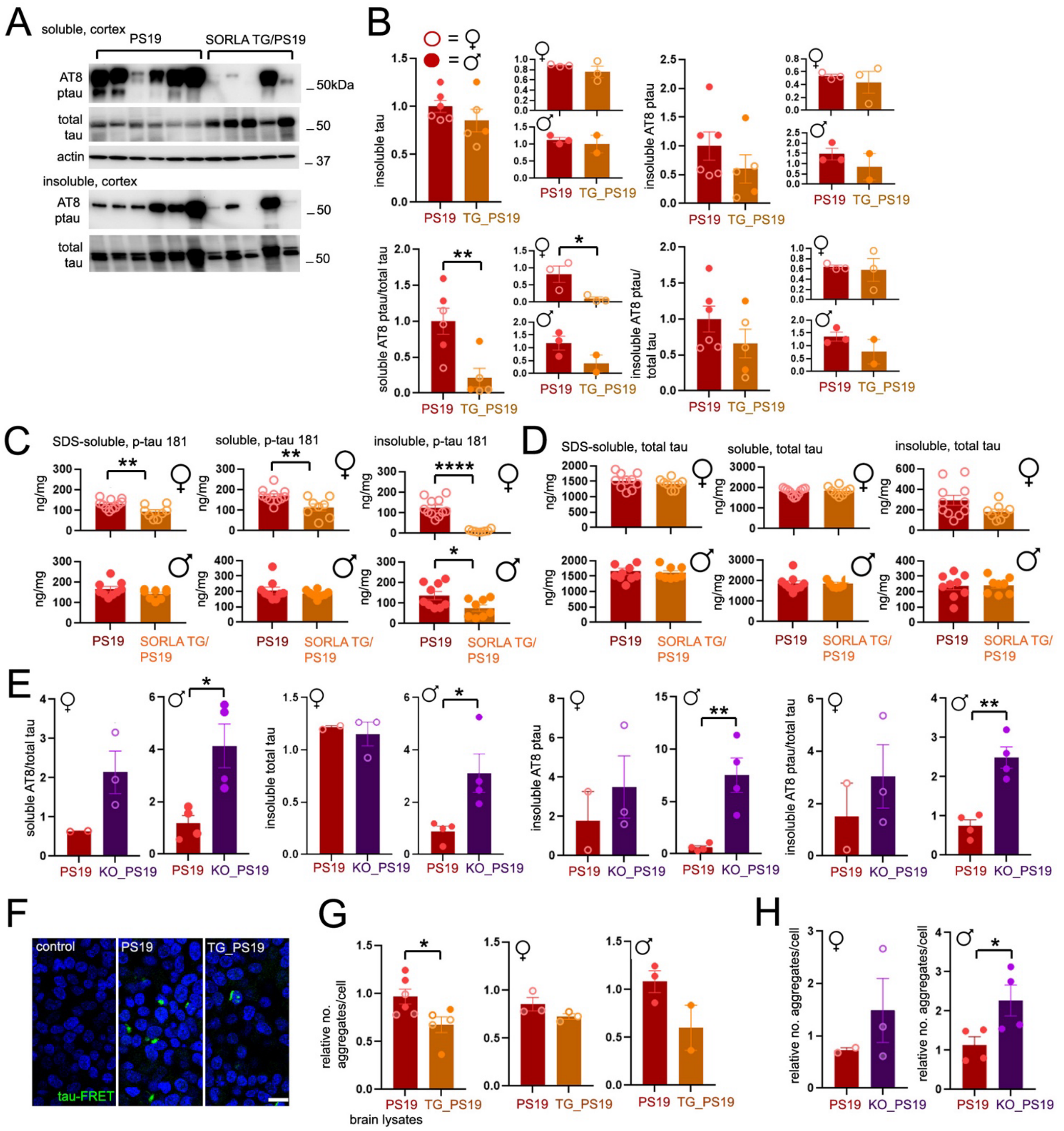

figure S2. Characterizing effects of SORLA modulation on tau solubility and seeding in PS19 brain. (A) Representative AT8 ptau and total tau immunoblots from soluble and insoluble fractions in 11 MO PS19 and SORLA TG/PS19 (TG_PS19) cortex. (B) Graphs depict insoluble tau or AT8 ptau or soluble/insoluble AT8 ptau/total tau ratios normalized to PS19 (set to 1.0) (PS19: 3M, 3F; TG_PS19: 2M, 3F). (C, D) ELISA quantification of p-Tau181 and total tau in RIPA–SDS–soluble, RIPA-soluble, and sarkosyl-insoluble brain fractions from female and male 9MO PS19 mice with or without SORLA overexpression. (E) Graphs depict insoluble tau or AT8 ptau or soluble/insoluble AT8 ptau/total tau ratios normalized to PS19 (set to 1.0) in cortical extracts from female and male 7MO PS19 and SORLA KO/PS19 (KO_PS19) mice. (F) Representative tau-FRET images (green) or DAPI (blue) images from HEK293 tau-RD biosensor cell lines exposed to lysates from 11MO PS19 or SORLA TG/PS19 cortex tissues, bar=20um. (G) Graphs depicting relative number of tau-FRET aggregates from 11MO PS19 and SORLA TG/PS19 cortex from (F) normalized to PS19 (set to 1.0). (H) Relative number of tau-FRET aggregates in HEK293 tau-RD biosensor cells exposed to cortical extracts from female and

male 7MO PS19 and SORLA KO/PS19 cortical extracts, normalized to PS19 (set to 1.0). Graphs depict mean±SE, statistical significance was determined by unpaired Student's t-test, *p<0.05, **p<0.01, ****p<0.0001.

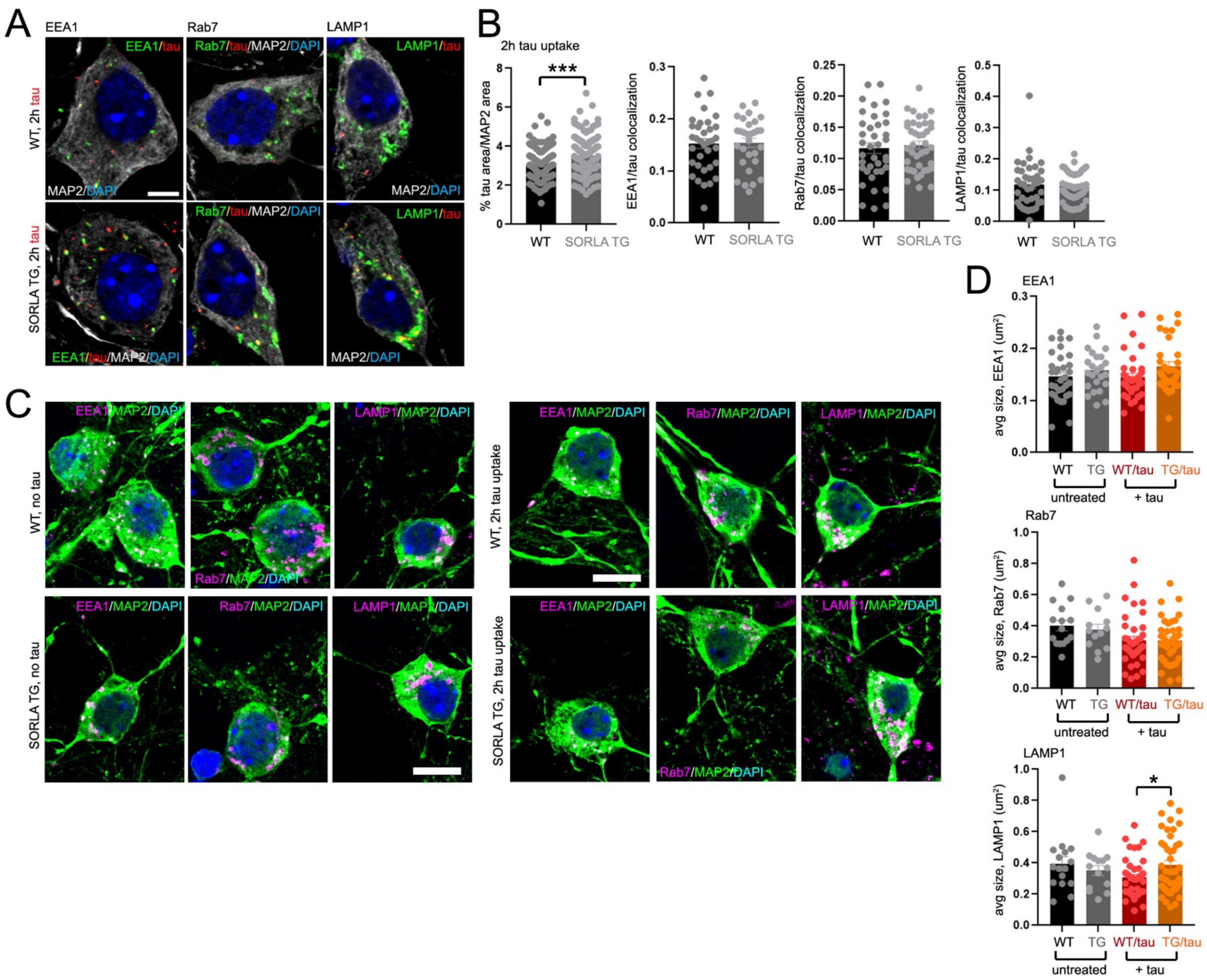

figure S3. Characterizing effects of SORLA overexpression on tau uptake in primary neuron. (A) Representative images of WT or SORLA TG neurons at DIV14 exposed to tau oligomers (15 nM) for 2h, and fixed/stained to visualize EEA1, Rab7 or LAMP1 (green), T13 tau (red) or nuclei (DAPI, blue) colocalization. Neuron area was marked by MAP2 staining (white), bar=5um. (B) Quantification of internalized tau (% tau area within MAP2 boundaries), or Pearson's correlation values for tau colocalization with endolysosomal compartments indicated in WT or SORLA TG neurons from images in (A). Plots represent values from one imaged field, and all values were derived from three independent experiments. (C) Representative images of untreated DIV14 WT or SORLA TG neurons (left panels), or neurons treated 2h with tau oligomers (right panels) and stained for endolysosomal markers (EEA1, Rab7, LAMP1; purple), MAP2 (green) or nuclei (DAPI, blue) as indicated, bar=10um. (D) Quantification of EEA1, Rab7 or LAMP1 size in WT and SORLA TG (TG) neurons under untreated or tau-treated conditions. Graphs depict mean±SE, statistical significance was determined by unpaired Student's t-test (B), or one-way ANOVA (D), *p<0.05, ***p<0.001.

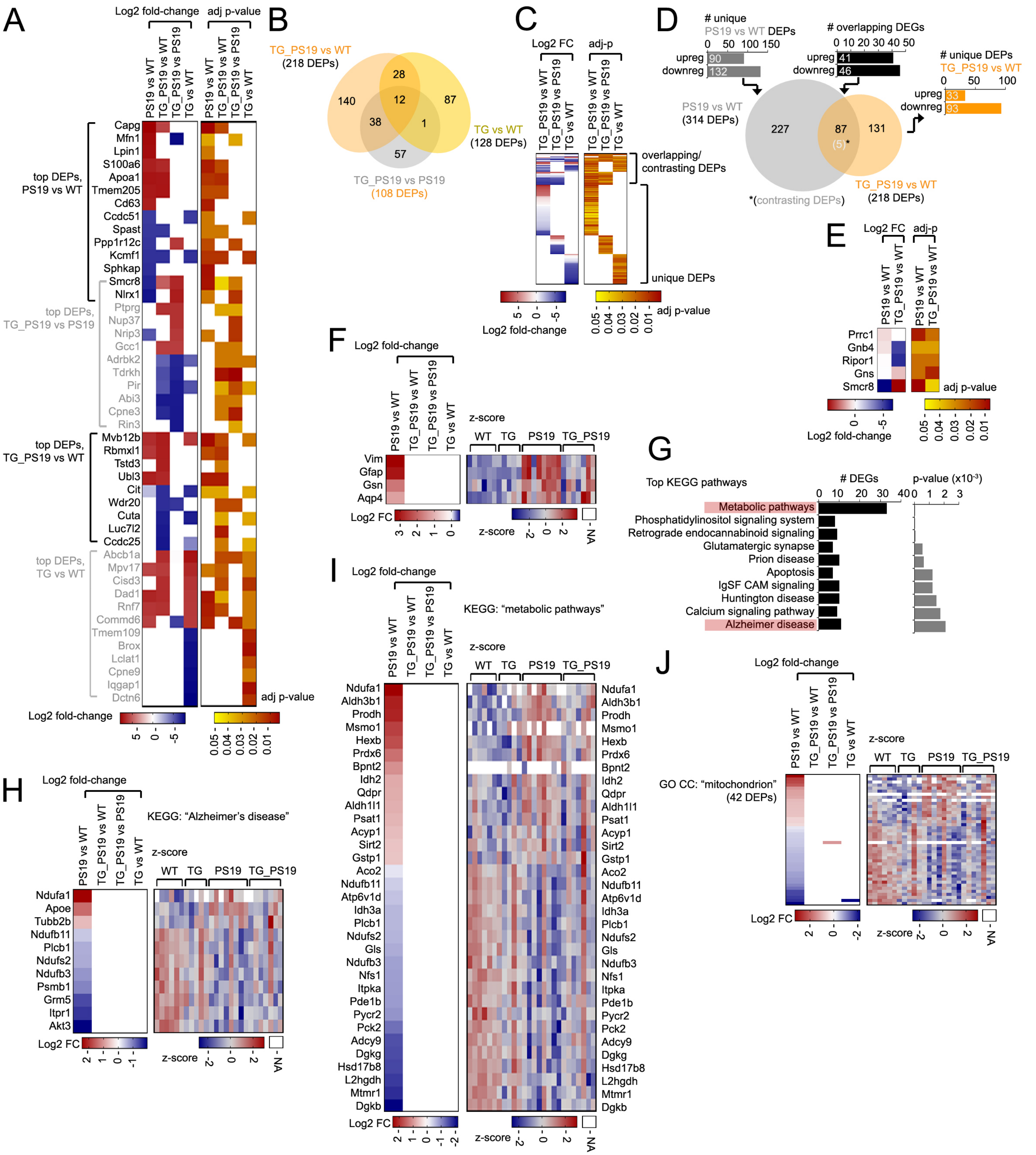

figure S4. Top DEPs identified and DEP overlap from proteomic analysis in PS19 and SORLA TG/PS19 mouse hippocampus. (A) Heatmaps depicting Log2 fold-change (left) and adj-p-value of top upregulated and

downregulated DEPs in PS19 vs WT, SORLA TG/PS19 (“TG_PS19”) vs PS19, TG_PS19 vs WT and SORLA TG (“TG”) vs WT comparisons. Some overlap and contrasting DEPs are observed between comparison groups. (B) Venn diagram depicting overlap between top DEPs from comparisons in (A). (C) Heatmaps indicating Log2 fold-change (left) and adj-p value (right) for the DEP comparisons shown. Grouped overlapping/contrasting and unique DEPs are indicated. (D) Venn diagram and graphs indicating the number of upregulated/downregulated unique and overlapping, as well as contrasting DEPs (indicated by an asterisk) in PS19 vs WT and TG_PS19 vs WT comparisons. (E) Heatmap indicating Log2 fold-change (left) and adj-p value (right) for contrasting DEPs in PS19 vs WT and TG_PS19 vs WT comparisons in (D). (F) Heatmaps showing Log2 fold-change for comparisons indicated (left) and z-score expression profiles (right) for the mouse genotypes shown for disease-associated astrocyte (DAA) genes shown. (G) Top 10 GO KEGG pathways enriched in the PS19 vs WT DEPs identified. (H, I) Log2 fold-change (left) and z-score (right) heatmaps for GO KEGG DEPs associated with “metabolic pathways” and “Alzheimer’s disease”. (J) Log2 fold-change (left) and z-score (right) heatmaps for 42 GO CC “mitochondrion” DEPs.

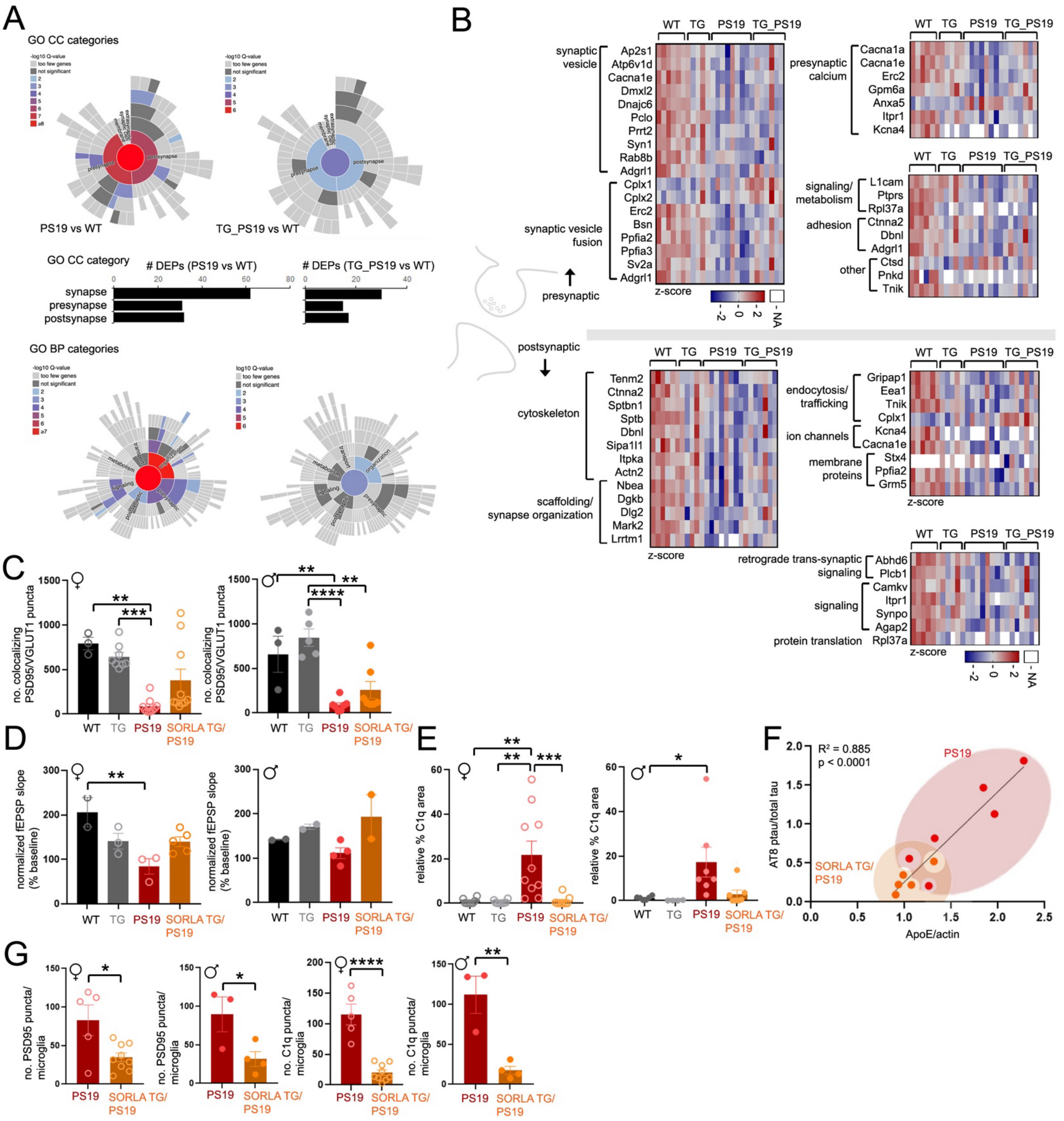

figure S5. Synaptic components changes in PS19 and SORLA TG/PS19 hippocampus identified by proteomic analysis. (A) Distribution of synaptic DEPs in PS19 vs WT, and TG_PS19 (SORLA TG/PS19) vs WT comparisons in GO cellular component (CC) (upper panels) or biological process (BP) (lower panels) synapse-related categories, identified using the synGO analysis tool (syngoportal.org). Colored heat maps indicate significance (-Log10 q-value) for DEPs in various GO CC and BP synapse-related categories shown. The number of total synapse, pre-synaptic or post-synaptic GO CC DEPs in PS19 vs WT or TG_PS19 vs WT comparisons are indicated in bar graphs. (B) Z-score distribution of presynaptic (upper panels) and postsynaptic (lower panels) DEPs from WT, TG (SORLA TG), PS19, and TG_PS19 hippocampus annotated by synGO analysis. Presynaptic and postsynaptic DEPs are clustered into various GO categories indicated. (C) Number of co-localizing PSD95/

VGLUT1 puncta in hippocampus region quantified by IMARIS from 9 MO female and male mice. (D) Cumulative fEPSP slopes during the last 10 mins. of recording (50-60 mins. after induction) averaged from individual female and male animals of each genotype. (E) Quantified relative percentage of C1q staining area in hippocampus region from 9 MO female and male mice. (F) Correlation between hippocampal AT8 ptau and Apoe levels from 9 MO mice based on relative band intensity values quantified in Fig. 4C, D. (G) Quantification of PSD95 and C1q puncta internalized per microglia in hippocampus from 9 MO female and male mice. All graphs represent mean±SE. Statistical analysis was determined by Two-way ANOVA with Tukey's multiple comparisons (C, D, E). Statistical significance was determined by unpaired Student's t-test in (G). $^{*}p<0.05$, $^{**}p<0.01$, $^{***}p<0.001$, $^{****}p<0.0001$.

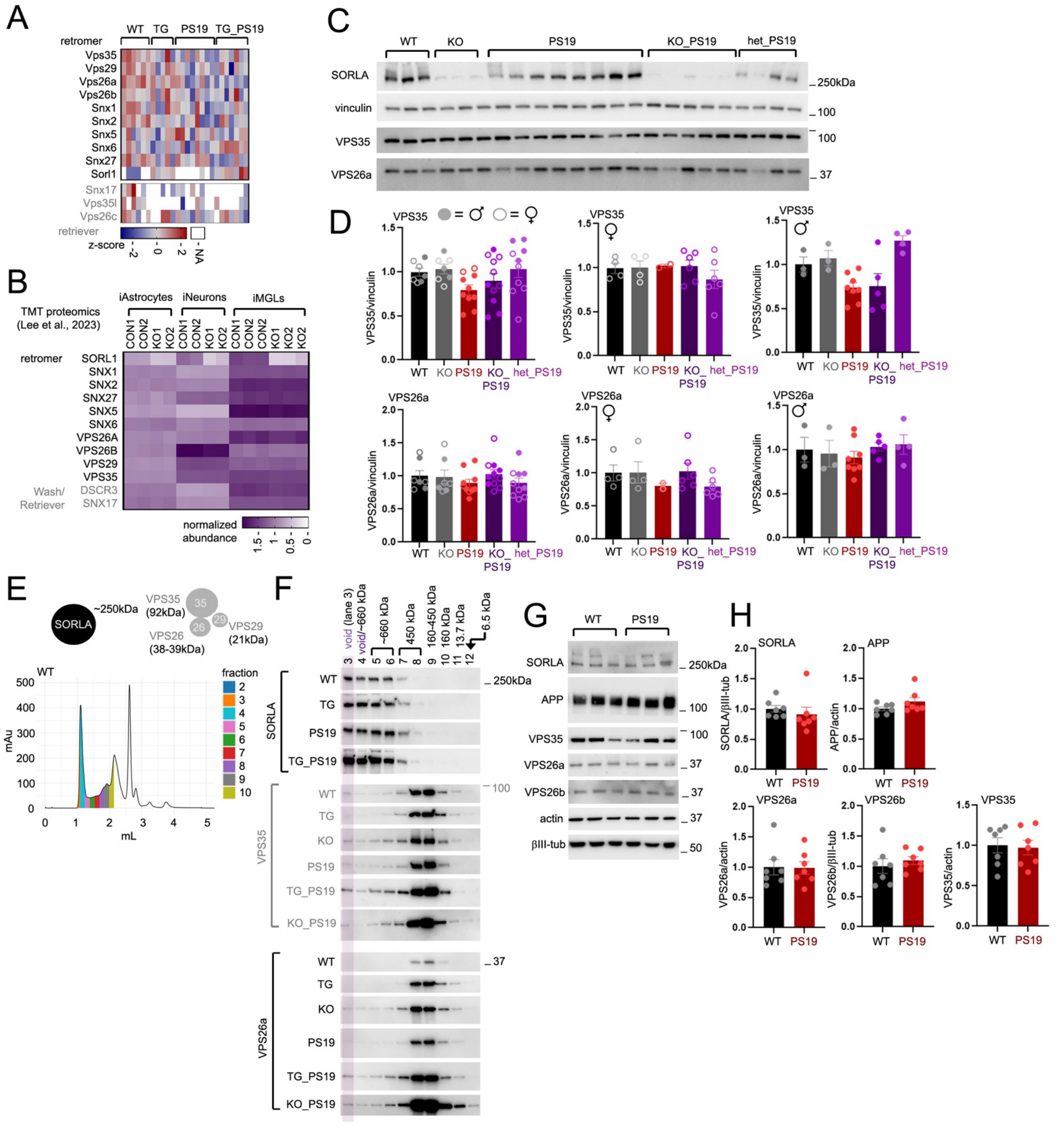

figure S6. Effects of SORLA modulation on the retromer complex. (A) Heatmap depicting z-score profiles of retromer/retriever components identified in 9MO WT, SORLA TG (TG), PS19, and SORLA TG/PS19 (TG_PS19) hippocampus. (B) Relative abundance of retromer and retriever components identified in iPSC-derived control ("CON") and SORLA KO astrocytes, neurons and iMGLs ("KO") as characterized by TMT-proteomics (Lee et al., 2023). (C, D) (C) Immunoblot analysis of retromer levels in 7MO mouse WT, KO, PS19, KO_PS19 and SORLA het KO/PS19 (het_PS19) hippocampus. (D) Quantification of VPS35 and VPS26a levels in mouse hippocampus from (C). (E, F) Characterization of high molecular weight SORLA and retromer complexes in mouse brain. (E) Schematic (upper panel), predicted molecular weight of SORLA and core retromer

components. Lower panel showing SEC elution profiles of WT mouse brain; fractions are color-coded in the elution fractions, milli-Absorbance units (mAu) within the elution profiles are shown. (F) Elution profiles of SORLA, VPS35 and VPS25a in 1.5MO WT, TG, KO, PS19, SORLA TG/PS19 (TG_PS19) and SORLA KO/PS19 (homozygous KO_PS19) mouse brain. Void volume (fraction 3 and a portion of fraction 4) is indicated. (G, H) (G) Representative immunoblot of retromer components in DIV14 primary neurons. (H) Quantification of retromer components and APP from immunoblots. No statistical significance was detected by unpaired Student's t-test (H) or one-way ANOVA (D) between the samples tested.

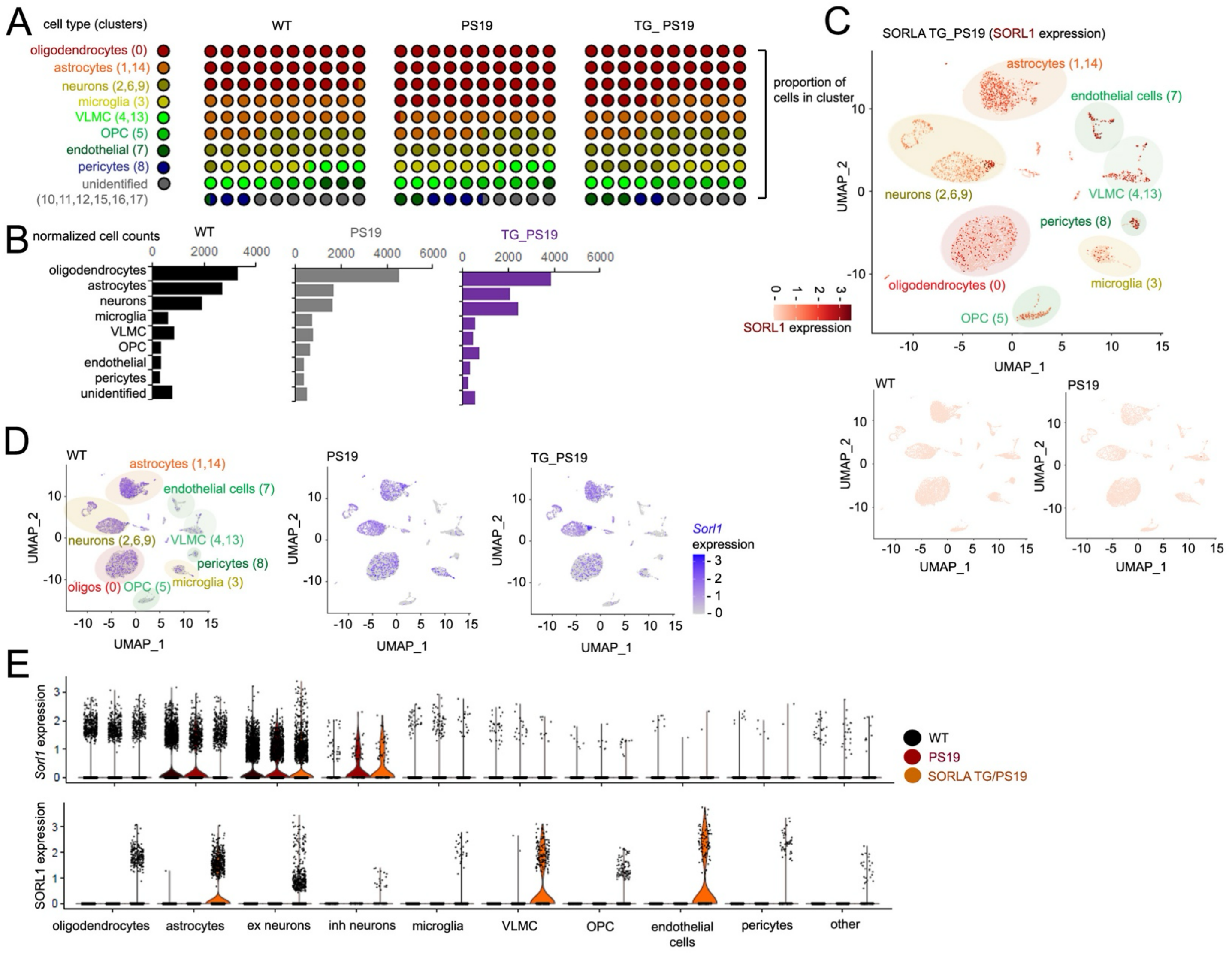

figure S7. Characterization of snRNAseq profiles from PS19 and SORLA TG/PS19 hippocampus. (A, B) 10x10 plot (A) and bar graph (B) showing relative distribution of cell types indicated in nuclei identified from 9MO WT, PS19 and SORLA TG/PS19 hippocampus. (C, D) Umap comprising all clustered nuclei from CNS cell types indicated in WT, PS19 and TG_PS19 hippocampus; heat-scales indicate expression of the human *SORL1* transgene ("expression") (C) or murine *Sorl1* expression (D). (E) Violin plots depicting murine *Sorl1* (upper graph) or human SORLA (SORL1) expression (lower graph) in WT (black), PS19 (red), or SORLA TG/PS19 (orange) hippocampus in the cell types indicated by snRNA-seq analysis. No human *SORL1* expression was detected in WT and PS19 mouse hippocampus in (C, E).

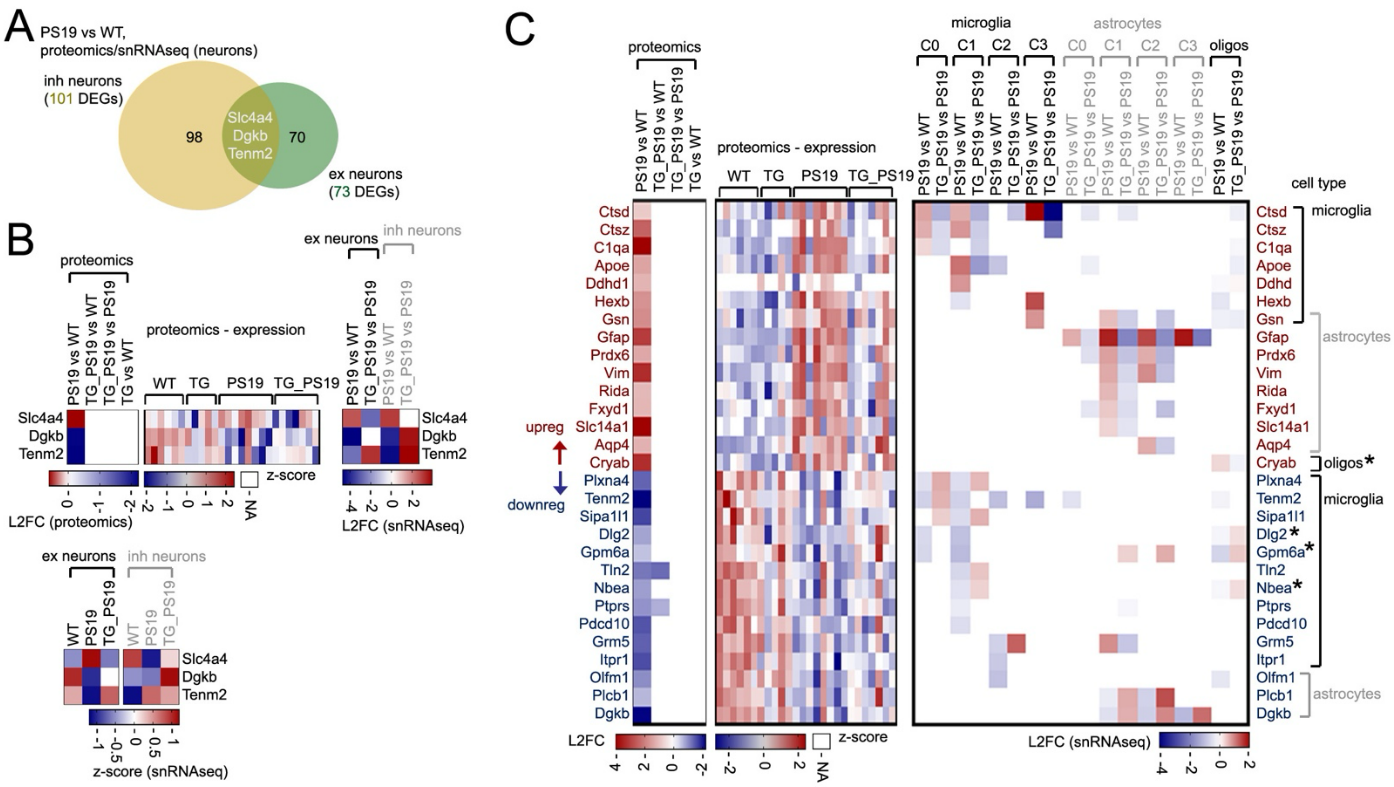

figure S8. Mapping bulk proteomics to cell-type expression profiles derived from snRNA-seq. (A) Venn diagram showing overlap between overlap between PS19 vs WT profiles identified by proteomic and snRNA-seq analysis in inhibitory ("inh", gold) and excitatory ("ex", green) neurons. (B) Heatmaps depicting Log2-fold change (left) and z-score (middle) DEP/protein, and Log2-fold change (right) and z-score (bottom) DEG/transcript values for 3 overlapping DEPs/DEGs in excitatory and inhibitory neurons. (C) Heatmaps showing bulk proteomic PS19 vs WT DEP Log2 fold-change (left) and z-score expression (middle) profiles mapped to DEGs (Log2 fold-change, right) identified in microglia, astrocytes and oligodendrocytes by snRNA-seq analysis; PS19 vs WT DEPs/DEGs that showed reversed profiles in TG_PS19 hippocampus are indicated. Asterisks indicate potential DEPs mapped to oligodendrocyte transcripts.

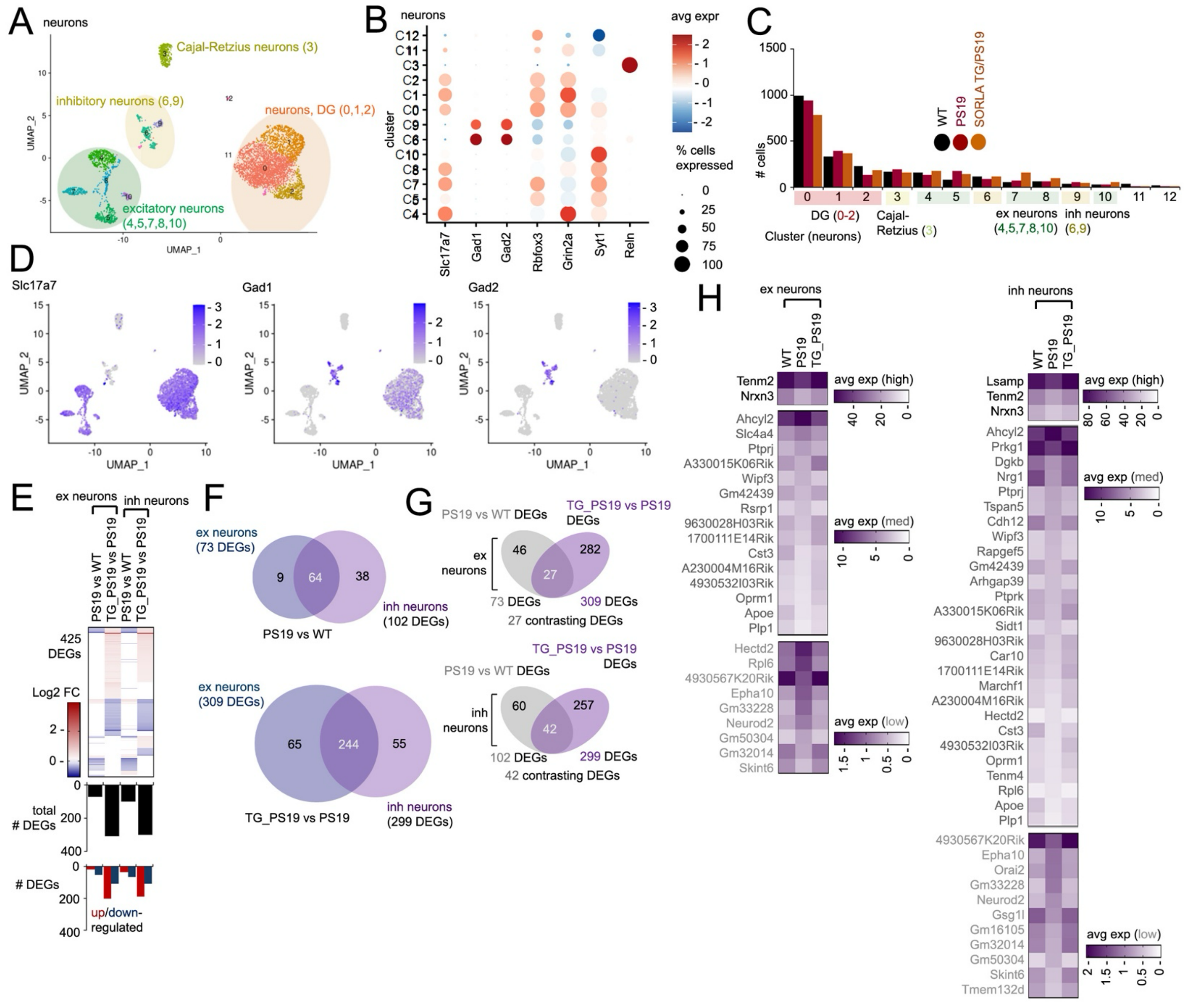

figure S9. Characterizing neuronal snRNA-seq profiles in mouse hippocampus. (A) Umap distribution of 5,425 reclustered neuronal nuclei. (B) Dot plot featuring cluster-specific expression markers in neurons; scales for percentage cell expression (plot size) and expression (red/blue heatmap scale) are shown on the right. (C) Bar graph showing number of cells normalized for total cell number per genotype observed in WT, PS19 and TG_PS19 neurons from each neuronal subcluster. (D) Expression of Slc17a7 and Gad1, 2 within reclustered neuronal nuclei. Heat-scaled Umap depicts excitatory (Slc17a7) and inhibitory (Gad1, 2) gene expression scores for reclustered neuronal nuclei. (E, F) (E) Heatmap depicting all excitatory and inhibitory neuronal DEGs identified in PS19 vs WT and TG_PS19 vs PS19 comparisons. Underlying bar graphs depict total number of DEGs (black bars) and up/downregulated (red/blue bars) in excitatory/inhibitory neurons. (F) Venn diagram showing number of overlapping DEGs in excitatory ("ex") and inhibitory ("inh") neurons in PS19 vs WT (top panel) and TG_PS19 vs PS19 (bottom panel) comparisons. (G) SORLA upregulation attenuates PS19 expression profiles in excitatory and inhibitory neurons. Venn diagram showing opposing DEGs ("opposing features") in PS19 neurons (PS19 vs WT) reversed in SORLA TG hippocampus (TG_PS19 vs PS19) in excitatory or inhibitory neurons as indicated, including ex and inh neurons from the DG region. (H) Average expression values of excitatory/inhibitory neuron DEGs featuring opposing features between PS19 vs WT and TG_PS19 vs PS19

comparisons in (G). Average expression values of DEGs with high, intermediate (med) and low expression values are shown for WT, PS19 and TG_PS19 genotypes in excitatory and inhibitory neurons as indicated.

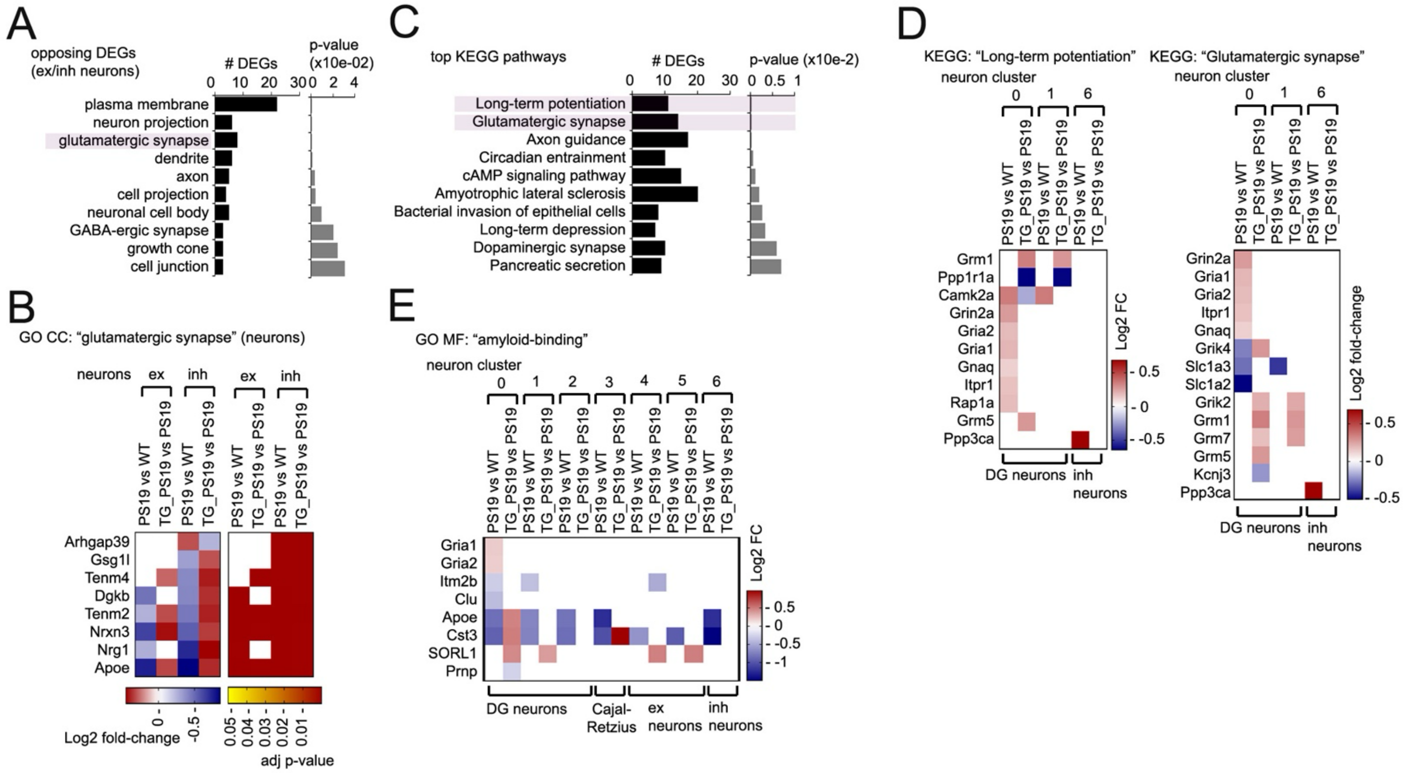

figure S10. Gene ontology analysis, DEGs in excitatory and inhibitory neurons. (A) GO analysis of 69 contrasting DEGs in PS19 and TG_PS19 neurons from excitatory and inhibitory neurons; top 10 GO CC categories are shown. (B) Heatmaps depicting Log2 fold-change (left heatmap) and adj p-value (right heatmap) of GO CC "glutamatergic synapse" DEGs identified in (A). (C) Top 10 KEGG pathways identified for DEGs in all neuronal clusters by GO analysis, graphs depict number of DEGs (black bars) and p-value (gray bars). (D) Heatmaps depicting cluster-specific gene changes in KEGG "Long-term potentiation" and "Glutamatergic synapse" categories. (E) Heatmap depicting cluster-specific gene changes related to GO MF (Molecular Function) "amyloid-binding" in neuron-specific DEGs.

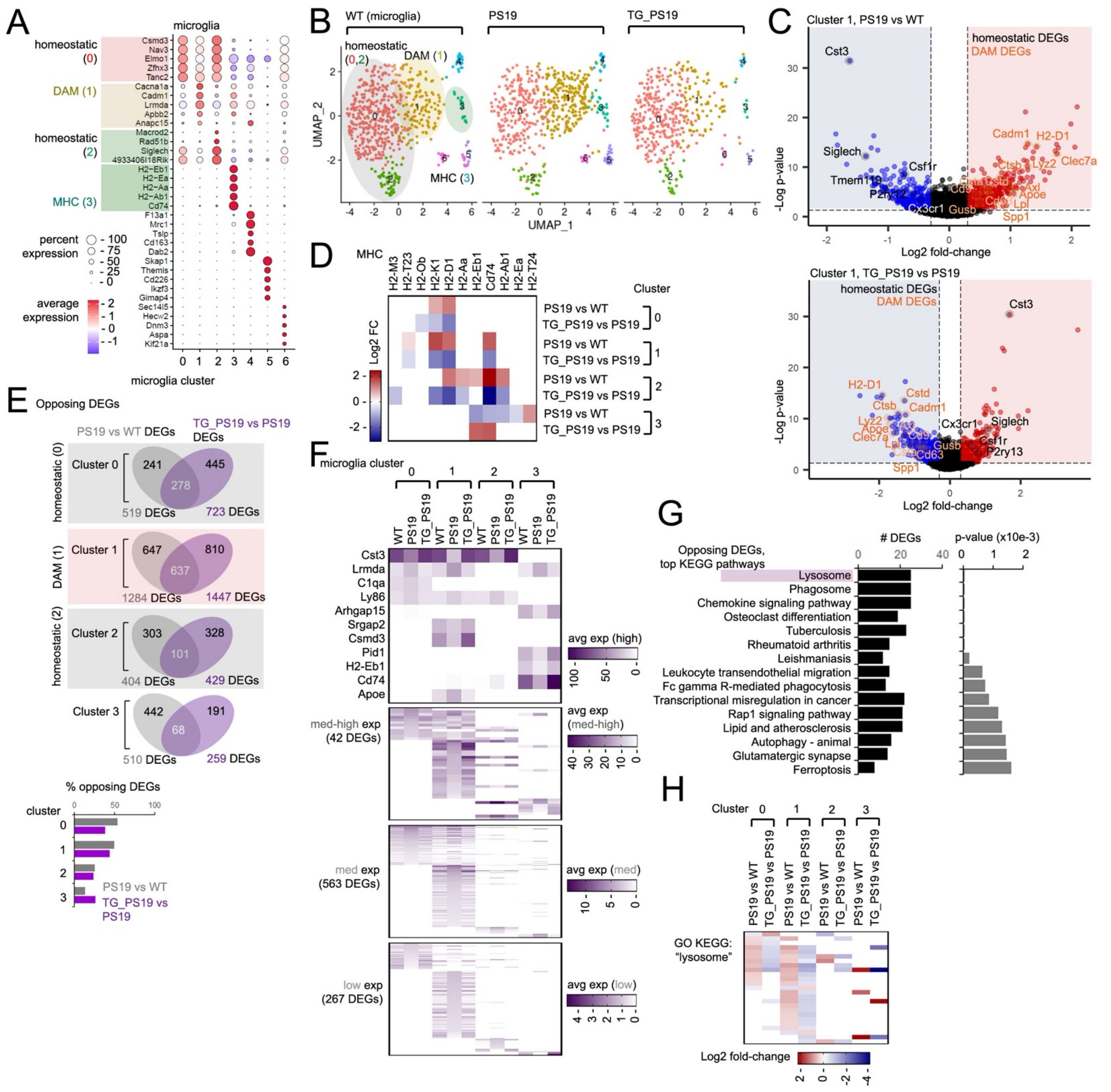

figure S11. Characterization of SORLA upregulation on microglia snRNAseq profiles in PS19 hippocampus. (A) Top 5 microglia genes defining the clusters indicated (clusters 0 to 6). Homeostatic (clusters 0 and 2), DAM (cluster 1) and MHC (cluster 3) subclusters are indicated. (B) Umap feature plots depicting microglia from WT, PS19 and TG_PS19 hippocampus; colored indicator labels mark homeostatic (red, green), DAM (dark yellow) and MHC (light green) subclusters. (C) Volcano plots depicting up and downregulated DEGs in PS19 vs WT and TG_PS19 vs PS19 microglia comparisons in microglia Cluster 1 (p-value<0.05; Log2 fold-change<-0.3, >0.3). Homeostatic (black) and DAM (orange) DEGs featuring opposing expression profiles in PS19 and TG_PS19 microglia are indicated. (D) Heatmap depicting Log2 fold-change (p<0.05) of MHC-related DEGs in reclustered microglia nuclei from Clusters 0 through 3. (E) Venn diagram depicting opposing DEG expression profiles in microglia Clusters 0 through 3 in PS19 (PS19 vs WT) and SORLA TG/PS19 (TG_PS19 vs PS19) hippocampus. Lower bar graph shows percentage of DEGs reversed in PS19 (PS19 vs WT, gray) and

TG_PS19 (TG_PS19 vs PS19, purple) microglia in the clusters indicated. (F) Average expression values of microglial DEGs in clusters indicated featuring opposing features between PS19 vs WT and TG_PS19 vs PS19 comparisons in (E). Average expression values of DEGs with high, medium-high (med-high), intermediate (med) and low expression values are shown for WT, PS19 and TG_PS19 genotypes in microglia subclusters 0 through 3 as indicated. (G) Top 15 GO KEGG pathways observed in PS19 microglia DEGs showing opposing expression profiles in SORLA TG/PS19 hippocampus. Bar graphs depict the number of DEGs and p-values for each KEGG pathway identified. (H) Expression profiles in opposing microglial DEGs associated with the "lysosome" GO KEGG pathway. Heatmaps depict Log2 fold-change (p<0.05) for lysosome DEGs in microglia Clusters 0 through 3.

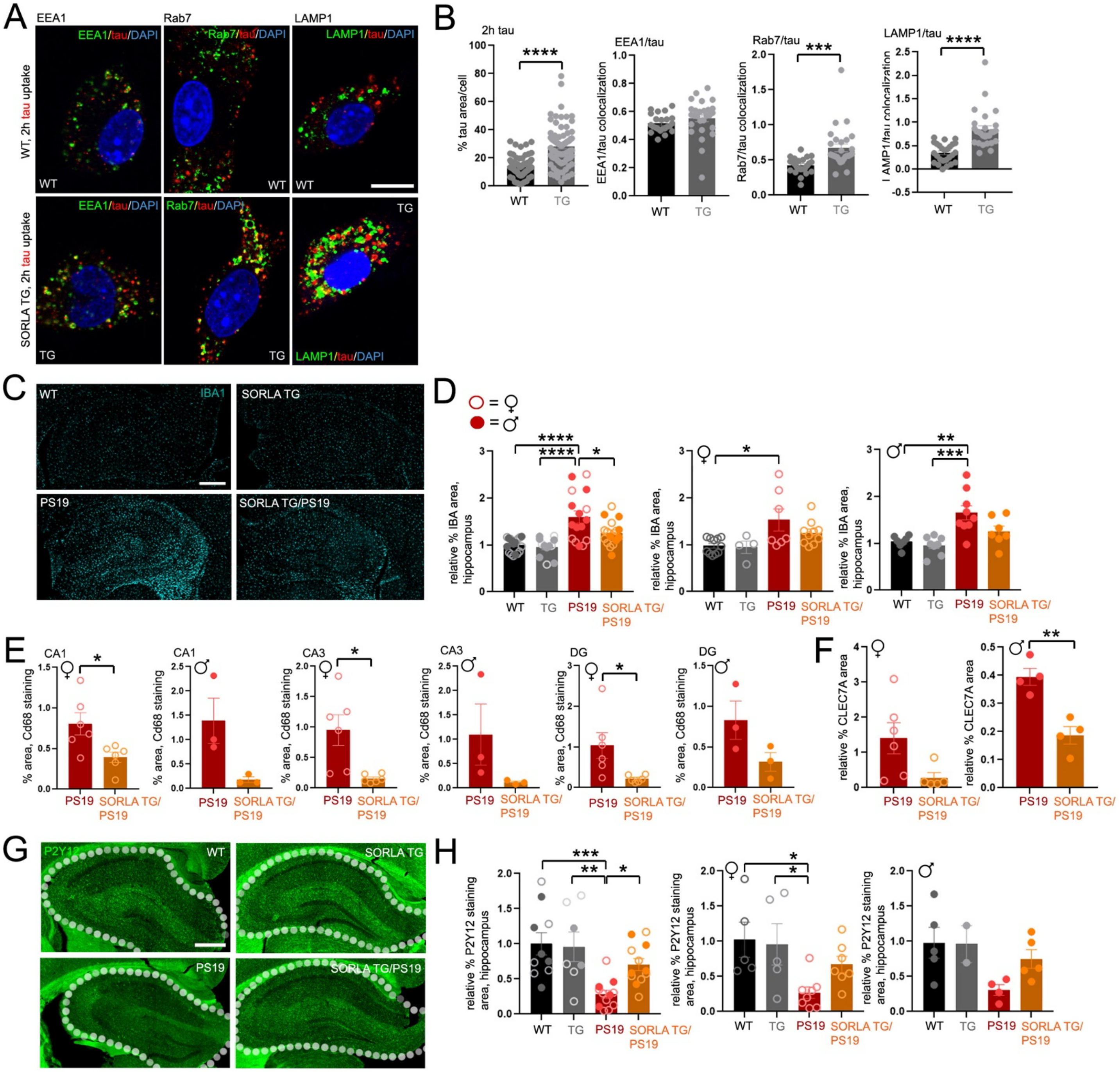

figure S12. Characterization of SORLA upregulation on tau uptake in cultured microglia and microglia activation in the PS19 hippocampus. (A, B) Effects of SORLA modulation on tau uptake in microglia.

Representative images of WT and SORLA TG (TG) microglia following 2h tau oligomer (15 nM) treatment; microglia were fixed and stained for endolysosomal markers (EEA1, Rab7, LAMP1; green), T13 tau (red) or nuclei (DAPI, blue), bar=10um. (B) Graphs depict quantification of tau uptake in WT and TG microglia (left graph), or EEA1, Rab7 and LAMP1 co-localization with tau following exposure to tau oligomers using Pearson correlation values. Plots represent values from one imaged field, and all values were derived from three independent experiments. (C) Representative images of 9MO WT, SORLA TG, PS19 or SORLA TG/PS19 hippocampus stained for IBA1 (cyan), bar = 500um. (D) Graph depicting relative IBA1 staining area per hippocampus from 9 MO mice (normalized to WT, set to 1.0). (E) Quantification of the relative percentage of CD68 staining area from different imaged regions in female and male 9 MO animals. (F) Quantification of the relative percentage of CLEC7A staining area from female and male 9 MO animals. (G) Representative images of P2Y12 (green) staining in 9MO WT, PS19, SORLA TG or SORLA TG/PS19 hippocampus, bar = 500um. (H) Quantification of the relative percentage of P2Y12 staining area from female and male 9 MO animals. All graphs represent mean±SE, where individual plots represent female (empty circles) and male (solid circles) animals. Statistical analysis was determined by Two-way ANOVA with Tukey's multiple comparisons in (D, H). Statistical significance was determined by unpaired Student’s t-test in (B, E). *p<0.05, **p<0.01, ***p<0.001, ****p<0.0001.

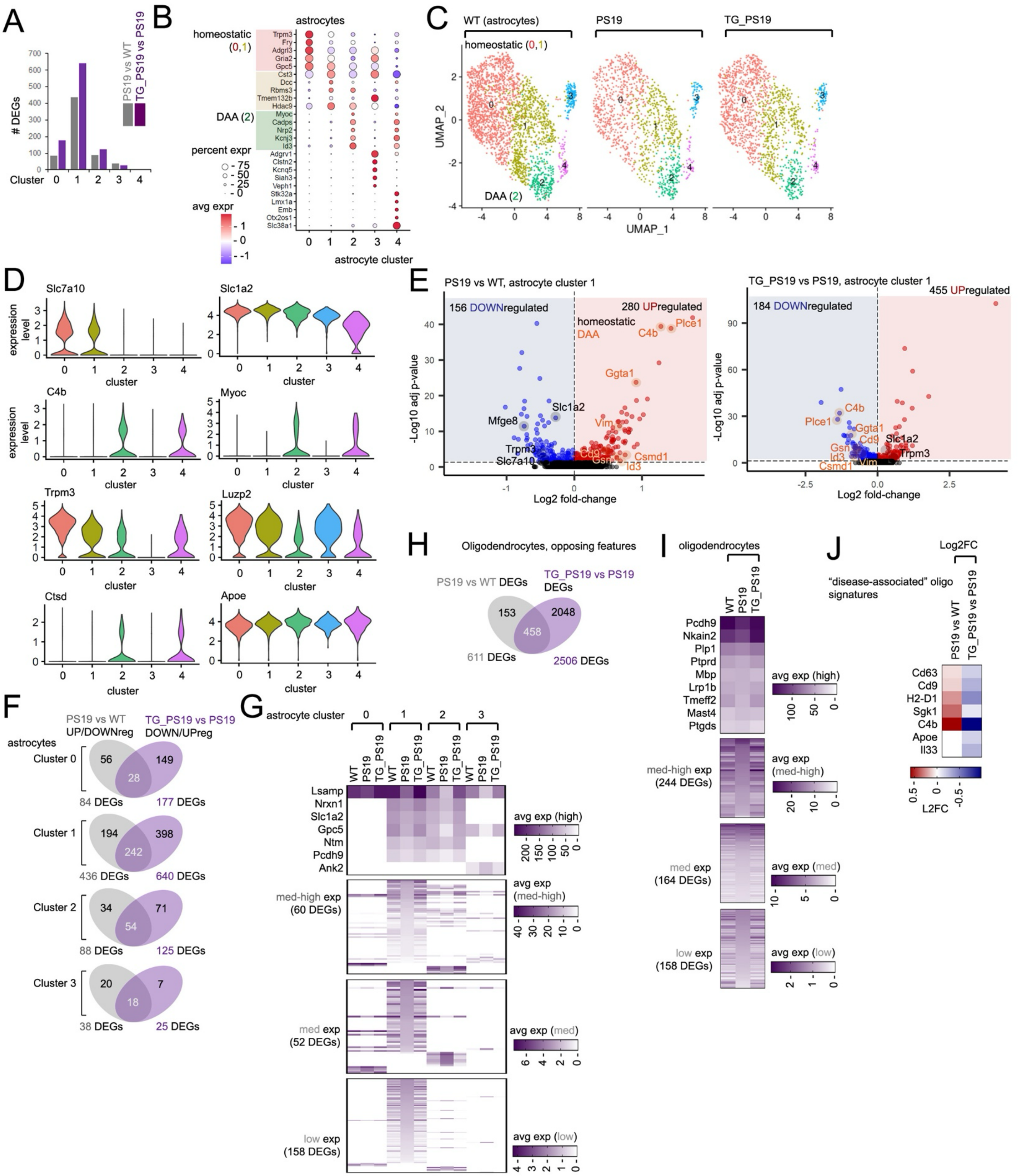

figure S13. SORLA upregulation attenuates astrocyte and oligodendrocyte transcriptomic profiles in PS19 hippocampus. (A) Number of DEGs identified from PS19 vs WT (gray) and TG_PS19 vs PS19 (purple) comparisons in astrocyte Clusters 0 through 4. (B) Top 5 astrocyte genes defining the clusters indicated (clusters 0 to 4). Homeostatic (clusters 0,1) DAA (cluster 2) subclusters are indicated. (C) Umap feature plots depicting microglia from WT, PS19 and TG_PS19 hippocampus; homeostatic (0,1 – red, yellow) and DAA (2 – green)

subclusters are indicated. (D) Violin plots showing expression profiles of genes indicated in reclustered astrocyte nuclei. (E) Volcano plots depicting up and downregulated DEGs in PS19 vs WT and TG_PS19 vs PS19 comparisons in astrocyte Cluster 1 (adj p-value<0.05). Homeostatic (black) and DAM (orange) DEGs featuring opposing expression profiles in PS19 and TG_PS19 microglia are indicated. (F) Venn diagram showing opposing DEGs ("opposing features") in PS19 astrocytes (PS19 vs WT) reversed in SORLA TG hippocampus (TG_PS19 vs PS19) in Clusters 0 through 3. (G) Average expression values of astrocyte DEGs in clusters indicated featuring opposing features between PS19 vs WT and TG_PS19 vs PS19 comparisons in (F). Average expression values of DEGs with high, medium-high (med-high), intermediate (med) and low expression values are shown for WT, PS19 and TG_PS19 genotypes in astrocyte subclusters 0 through 3 as indicated. (H) Venn diagram depicting opposing DEGs in PS19 oligodendrocytes reversed in SORLA TG hippocampus (TG_PS19 vs PS19). (I) Average expression values of oligodendrocyte DEGs featuring opposing features between PS19 vs WT and TG_PS19 vs PS19 comparisons in (H). Average expression values of DEGs with high, medium-high, intermediate and low expression values are shown for WT, PS19 and TG_PS19 genotypes in oligodendrocytes. (J) Heatmap depicting changes in "disease-associated" oligodendrocyte signatures in PS19 vs WT, or TG_PS19 vs PS19 oligodendrocytes; Log2 fold-change (L2FC) (adjp<0.05) is shown.

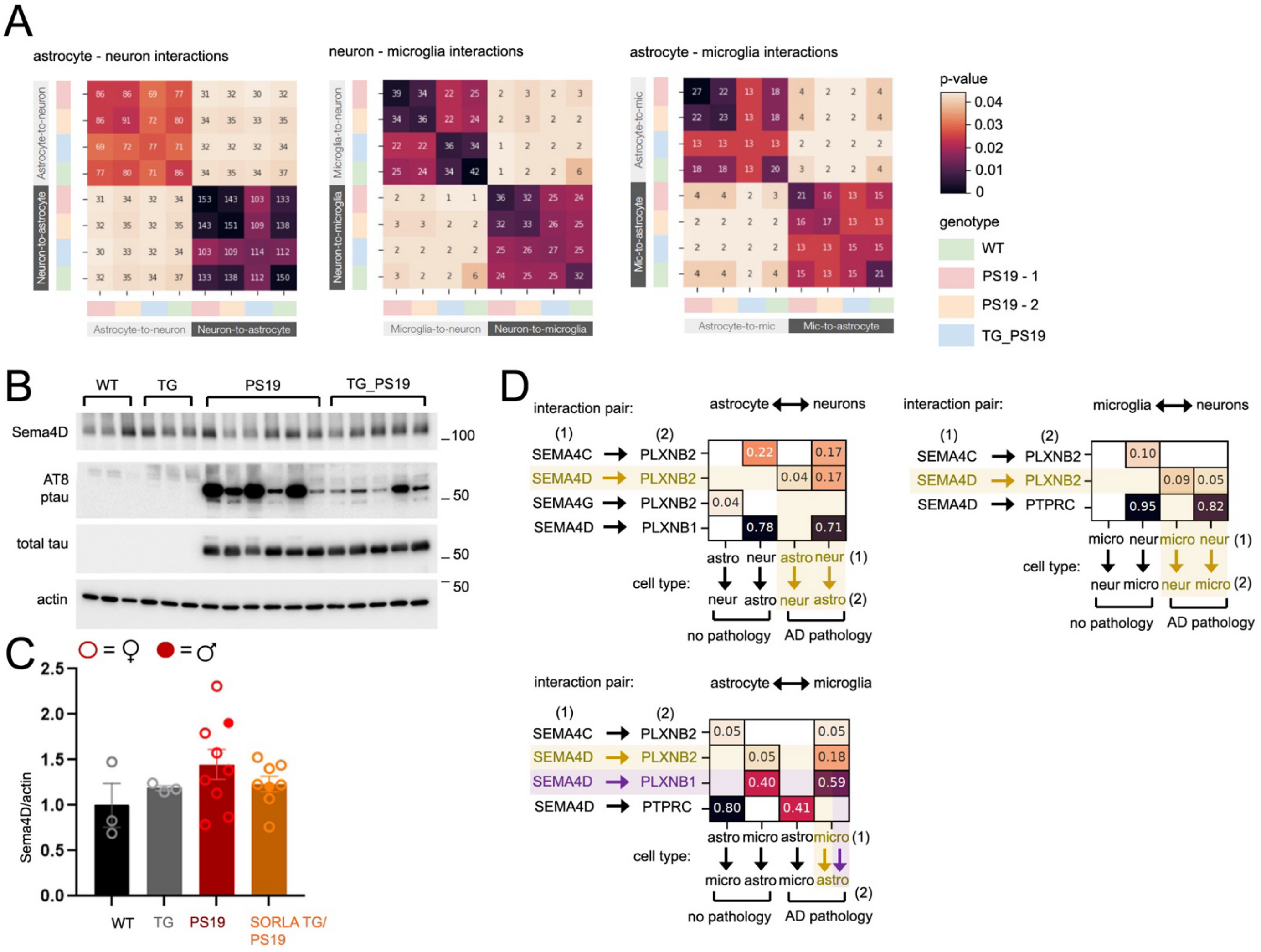

figure S14. Characterizing potential pathogenic PS19 targets modified by SORLA upregulation. (A) Characterizing changes in cell-cell interactions in transcripts derived from PS19 and SORLA TG/PS19 hippocampus. Matrices showing number of shared interactions identified by CellPhoneDB analysis between different cell types between WT (green), PS19 (red and orange each mouse) and TG_PS19 (blue) animals. (B, C)

(B) Immunoblots from 9MO WT, SORLA TG, PS19 or SORLA TG/PS19 (TG_PS19) hippocampal protein lysates to detect Sema4D, AT8 ptau, total human tau, or actin. (C) Quantification of Sema4D band intensity from blots in (B) normalized over actin, compared to WT (set to 1.0). (D) Characterizing *SEMA/PLXN* interactions in snRNA-seq profiles from human AD brain (Mathys et al., 2019). Matrices depicting the proportion of cells expressing *SEMA* (ligand)/*PLXN* (receptor) transcript pairs in astrocyte/neuron, microglia/neuron or astrocyte/microglia combinations. Ligand/receptor pairs are indicated on the side, and cell/cell interaction pairs are indicated on the bottom of the matrices; cells derived from AD samples with no pathology, or with AD pathology are indicated on the bottom. Cell proportion is scaled by color from lower percentages (tan) to higher percentages (orange, red, purple, black). Enrichment of *SEMA4D* and *PLXNB1* or *B2* interactions in AD brain are highlighted in yellow/purple. Graphs represent mean±SE. Statistical analysis was determined by Two-way ANOVA then Tukey's multiple comparisons test in (C).

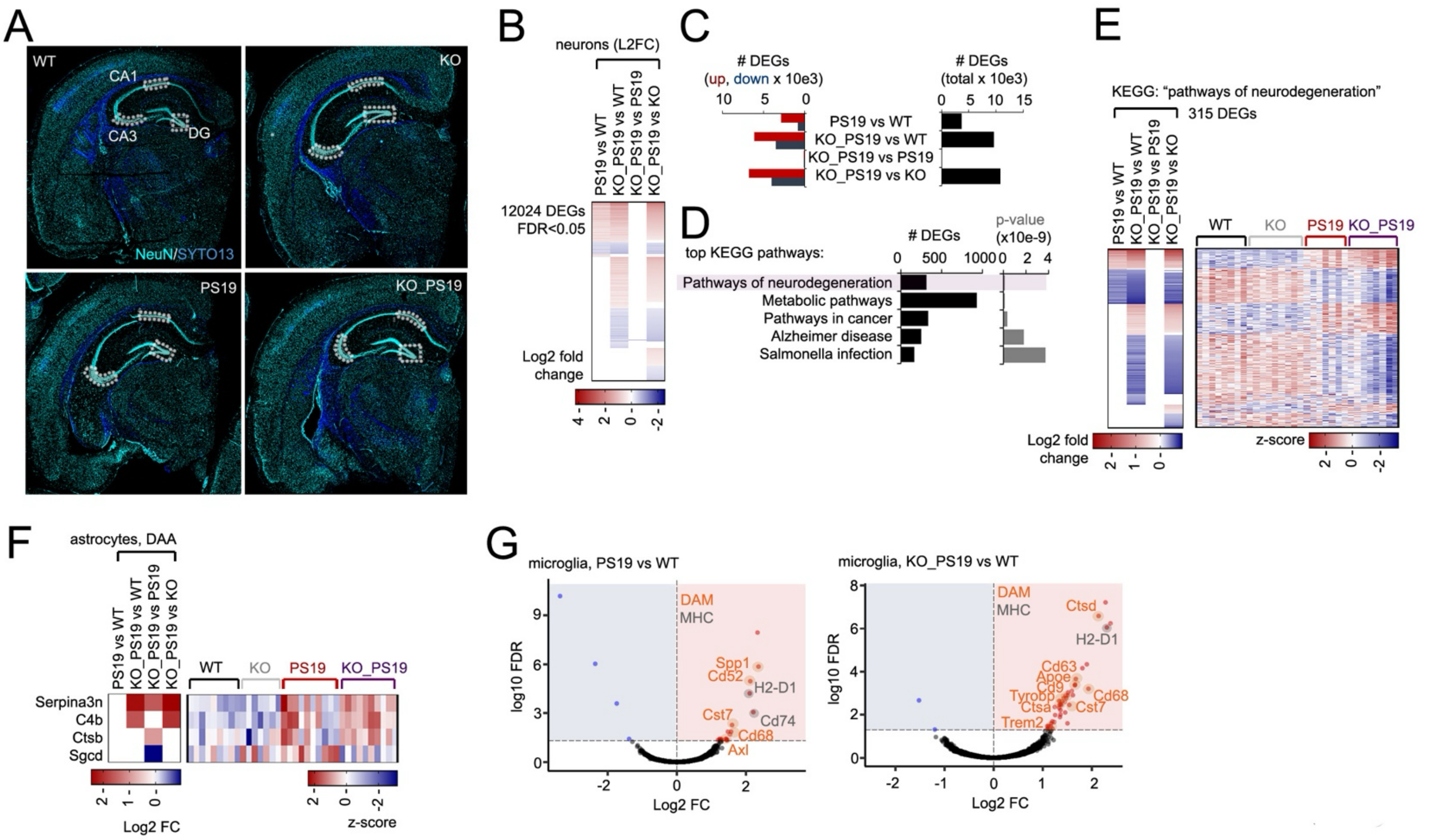

figure S15. Characterizing spatial transcriptomic profiles in neurons and glia in SORLA KO/PS19 hippocampus. (A) Brain hemisections stained for NeuN (turquoise) and nuclei (SYTO13, blue); ROI selections within CA1, CA3 and DG regions in 9MO KO, PS19 and KO_PS19 hippocampus for GeoMx analysis are indicated. (B) Heatmap showing Log2 fold-change (L2FC) of all DEGs identified in neurons in the genotype comparisons indicated (FDR<0.05). (C) Graphs indicate total number of DEGs (black bars, right graph) and number of upregulated/downregulated DEGs (red and blue bars, left graph) for each genotype comparison. (D) Top 5 GO KEGG pathways identified in neuronal DEGs by GeoMx analysis. (E) Log2 fold-change (left heatmap) of the genotype comparisons and z-score distribution (right heatmap) of WT, SORLA KO ("KO"), PS19 or SORLA KO/PS19 ("KO_PS19") neuronal DEGs enriched in the KEGG "pathways of neurodegeneration" category in neurons. (F) Log2 fold-change (left heatmap) and z-score distribution (right heatmap) of astrocyte DAA genes identified by GeoMX analysis. (G) Volcano plots depicting changes in microglial DAM (orange) or MHC (black) genes identified by GeoMX analysis (FDR<0.05).

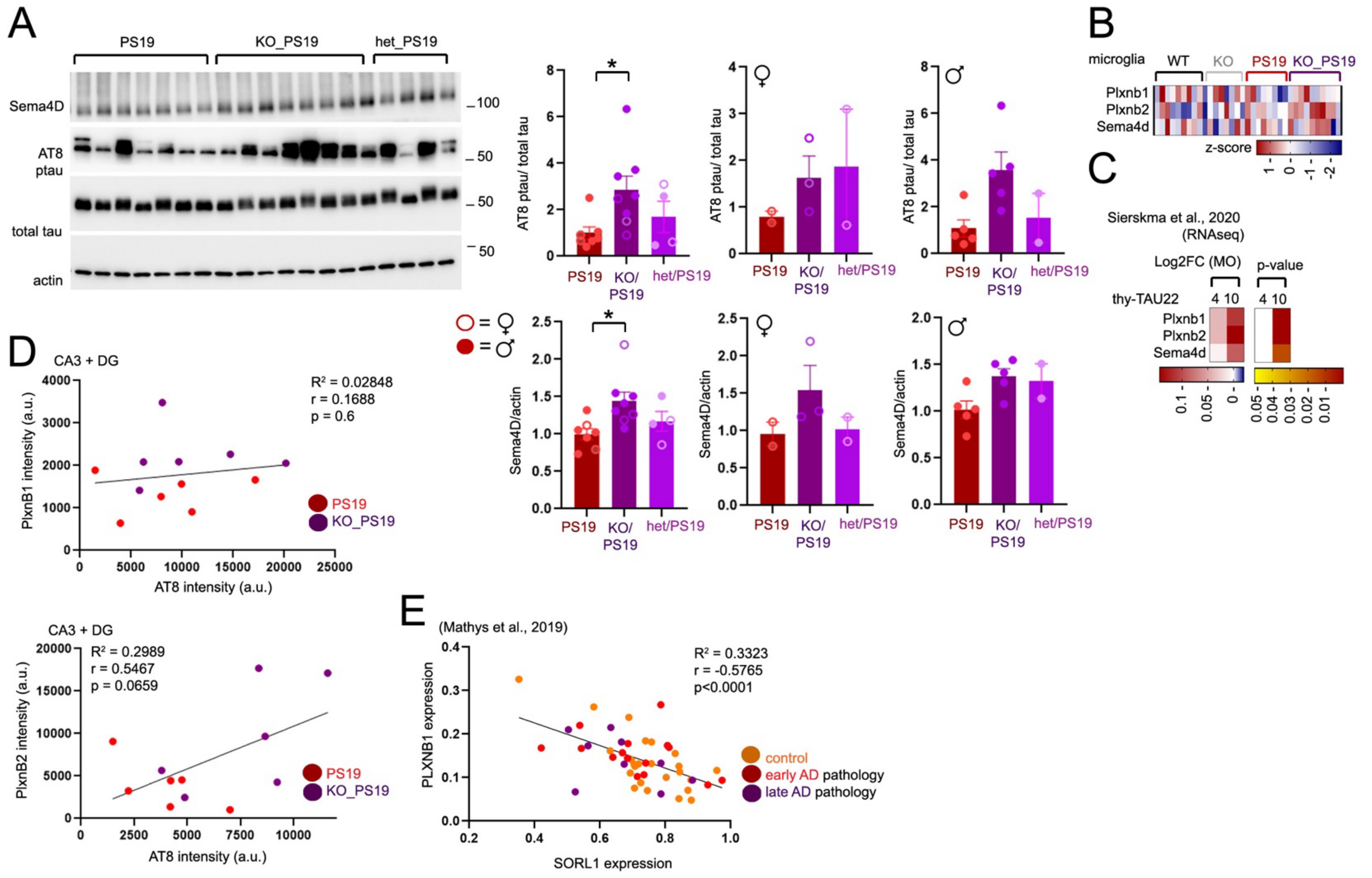

figure S16. Characterizing effects of SORLA modulation on neuronal/glial profiles and the Sema4d/PlxnB pathway in PS19 mouse hippocampus. (A) Immunoblots from 7MO WT, homozygous SORLA KO ("KO"), PS19, homozygous SORLA KO/PS19 ("KO_PS19") or heterozygous SORLA/PS19 ("het_PS19") hippocampal protein lysates to detect Sema4D, AT8 ptau, total human tau, or actin. Graphs: Quantification of AT8 ptau/total tau (upper graphs) or Sema4D band intensity normalized over actin (lower graphs) from blots shown, compared to PS19 (set to 1.0). (B) Heatmap depicting z-score distribution of Plxnb1, Plxnb2 and Sema4d in microglia in WT, KO, PS19 and KO_PS19 hippocampus by GeoMx analysis. (C) *Plxnb1/Plxnb2* and *Sema4d* expression levels in 4 or 10 month-old thy-TAU22 mouse brain (vs control) by bulk RNAseq analysis (Sierskma et al., 2020). Heat-scaled changes are indicated for Log2 fold-change (left), or p-value (right heatmap). (D, E) (D) Scatter plots showing correlation between PlxnB1 (upper panel) or PlxnB2 (lower panel) with AT8 ptau staining intensity in PS19 (red) and KO_PS19 (purple) CA3 and DG regions combined. (E) Scatterplot and linear regression analysis correlating *PLXNB1* expression and SORLA (*SORL1*) levels in human control (orange), and AD brain with early (red) and late (purple) AD pathology. Correlation coefficient of determination (R^2) as well as Pearson coefficient (r) and p-value were determined by simple linear regression, and shown for each comparison shown in (D) and (E). Data in (A) represent mean±SE and individual plots represent quantification from male (solid) and female (empty plots) animals. Statistical significance was determined by One-way ANOVA with Tukey's multiple comparison (**p<0.01, ****p<0.0001).

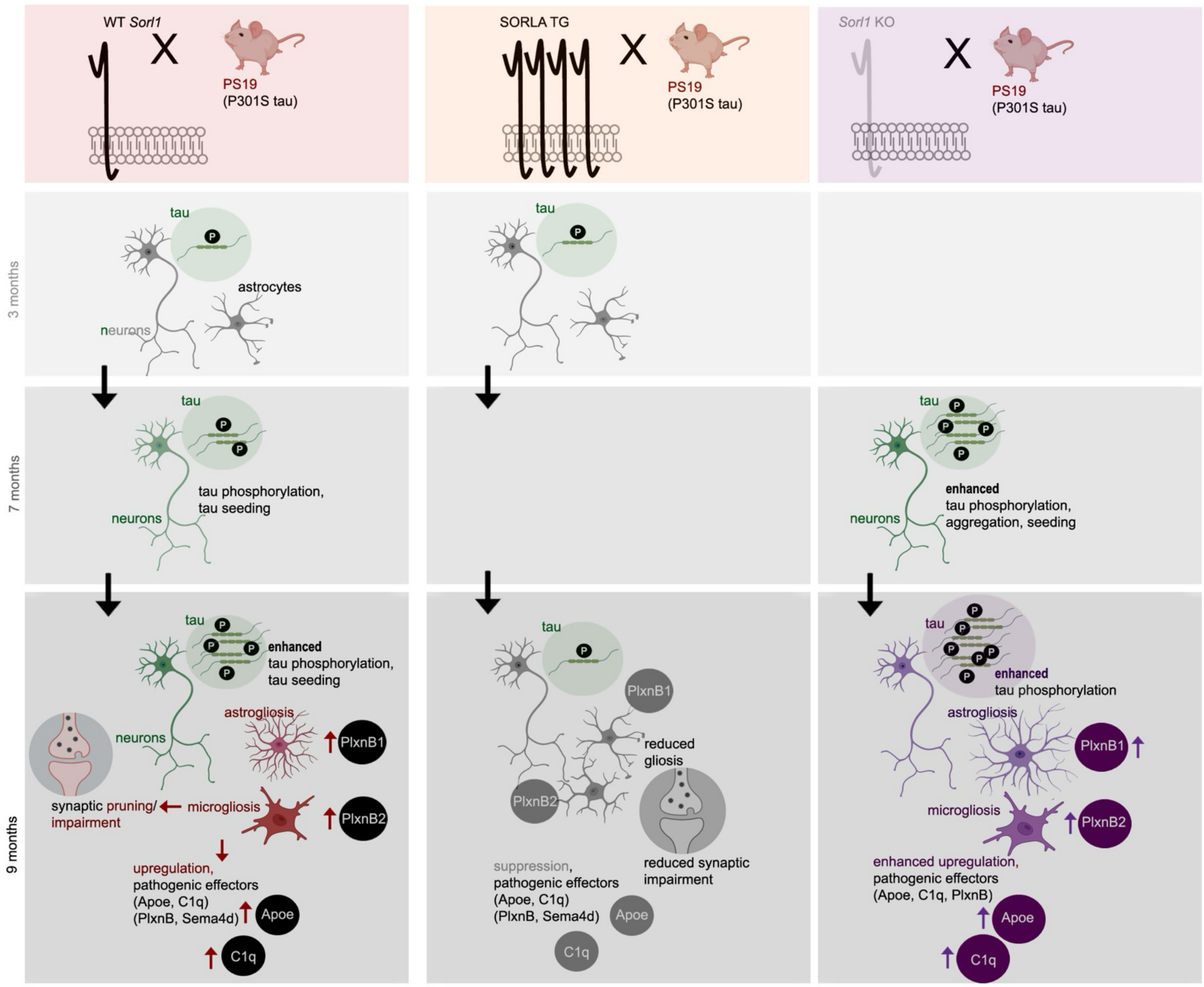

figure S17. Model depicting effects of SORLA modulation on tau pathology and pathogenesis in PS19 mouse brain. Comparison of pathological events in PS19 (red, left), SORLA TG/PS19 (orange, middle) and SORLA KO/PS19 (right, purple) mouse brain with age. Little AT8 tau phosphorylation is detected at 3 months of age in PS19 hippocampus, and little difference is observed in AT8 p-tau and GFAP levels in SORLA TG/PS19 animals at this timepoint. Tau aggregation and seeding is enhanced in SORLA KO/PS19 animals compared to PS19 at 7 months. In aged (9MO) PS19 mice, elevations in AT8-tau pathology and aggregation promote induction of pathogenic effectors such as Apoe and C1q in microglia, as well as PlxnB in astrocytes (PlxnB1) and microglia (PlxnB2). Microglia activation in PS19 brain results in enhanced synaptic pruning and uptake, thereby promoting synaptic impairment, whereas reduced tau pathology in SORLA TG/PS19 animals attenuates microglia activation and induction of pathogenic mediators (Apoe, C1q, PlxnB2) as well as astrocyte PlxnB1 and attenuates microglial synaptic pruning/uptake. Conversely, AT8 p-tau levels are enhanced in SORLA KO/PS19 mouse brain, thereby enhancing expression of glial Apoe, C1q and PlxnB expression and activation. Depictions of cells, mice and tau and synapses were created in BioRender ("fig. S17" by Huang, T. (2026), https://BioRender.com/20gnecx).

Supplemental Tables S1-S5

Table S1:
Label-free proteomic analysis, 9MO WT, SORLA TG, PS19, SORLA TG/PS19 hippocampus
Sheet 1 - "L2FC_significance", DEPs (adjp<0.05)

Sheet 2 - "PS19 vs WT and TG_PS19 vs WT" comparisons: contrasting DEPs indicated in red, overlapping DEPs indicated in green and unique DEPs indicated in black
Sheet 3 - z-score values across individual animals for significant DEPs, "NA" indicates missing peptide values

Table S2:
GO analysis, proteomics
Sheet 1 - "GO_CC_227 PS19 DEPs", GO CC (cellular component) categories, 227 DEPs unique to PS19 vs WT comparisons

Table S3:
snRNA-seq analysis, 9MO WT, PS19 and SORLA TG/PS19 hippocampus
Sheet 1 - Microglia, clusters C0 to C3, L2FC>0.3, <-0.3; p<0.05
Sheet 2 - Astrocytes, clusters C0 to C4, adjp<0.05
Sheet 3 - Neurons, clusters C0 to C12, adjp<0.05
Sheet 4 - Oligodendrocyte DEGs, adjp<0.05

Table S4:
GeoMx analysis (DEGs, z-scores), 9MO WT, SORLA KO, KO/PS19, PS19 hippocampus
DEGs and z-scores for astrocytes, microglia and neurons are shown

Table S5:
Female and male numbers in individual Figure panels
Two-way ANOVA analysis results in individual Figure panels
